# HJURP Promotes Malignant Progression and Mediates Sensitivity to Cisplatin and WEE1-inhibitor in Serous Ovarian Cancer

**DOI:** 10.7150/ijbs.65589

**Published:** 2022-01-01

**Authors:** Zhiyuan Dou, Chunping Qiu, Xun Zhang, Shu Yao, Chen Zhao, Zixiang Wang, Ran Chu, Jingying Chen, Zhongshao Chen, Rongrong Li, Kun Wang, Penglin Liu, Chang Liu, Kun Song, Beihua Kong

**Affiliations:** 1 Department of Obstetrics and Gynecology, Qilu Hospital of Shandong University, Jinan, P.R. China.; 2 Gynecologic Oncology key Laboratory, Qilu Hospital of Shandong University, Jinan, P.R. China.

**Keywords:** HJURP, WEE1, AZD1775, chemoresistance, DNA damage repair

## Abstract

Ovarian cancer is the most lethal gynecological malignancy. Recurrence and chemoresistance are tough challenges leading to poor prognosis. HJURP is a molecular chaperone of CENP-A, which is associated with aggressive progression in multiple tumors. However, the function of HJURP in ovarian cancer has not been elucidated. In our study, we found HJURP was over-expressed in ovarian cancer and high expression of HJURP was correlated to unfavorable prognosis. HJURP knockdown could inhibit proliferation, metastasis and induce G0/G1 stagnation of ovarian cancer cells. Besides, next-generation sequencing (NGS) unveiled that WEE1 was down-regulated by silencing HJURP. Further mechanistic research revealed that HJURP regulated WEE1 through MYC, and luciferase assay indicated that MYC was a transcription factor of WEE1. Additionally, we investigated that silencing HJURP increased sensitivity of ovarian cancer cells to cisplatin via MYC/WEE1 axis, and HJURP participated in DNA repair of cisplatin-induced damage. More interestingly, silencing HJURP could enhance sensitivity of ovarian cancer cells to AZD1775 and improve the synergistic effect of cisplatin plus AZD1775 combined therapy. Collectively, our data displays that HJURP promotes tumor progression and chemoresistance of ovarian cancer, and HJURP has potential to be a novel therapeutic target in the combined treatment with cisplatin and AZD1775 in ovarian cancer.

## Introduction

Ovarian cancer is the most lethal gynecological malignant disease worldwide with approximately 21,750 newly-diagnosed cases and 13,940 tumor-associated deaths in America in 2020^1^. High-grade serous ovarian cancer, which was generally diagnosed at an advanced stage with disseminated abdominal transfer, is the worst histological type with poor prognosis, accounting for 70-80% of ovarian cancer deaths^2^,^3^. Although poly (adenosine diphosphate-ribose) polymerase inhibitor (PARPi) has shown some beneficial effects in the maintenance therapy, surgical-debulking followed by platinum-based chemotherapy is still standard treatment for ovarian cancer patients^4^-^6^. Therapeutic strategies were nonspecific and restricted for the heterogeneous disease. Recurrence and chemoresistance are still tough challenges leading to poor prognosis. Therefore, novel therapeutic targets and prognostic biomarkers were urgently to be explored to overcome malignant progression and chemoresistance.

HJURP, a histone H3 chaperone, was first identified and described by Kato T, et al. in non-small cell lung cancer, which mediated centromeric chromatin assembly, maintenance and deposition of CENP-A nucleosomes^7^-^9^. CENP-A is the marker of centromere, which could mediate chromosome segregation and protect against aneuploidy^10^,^11^. HJURP is possibly involved in the chromosomal stability and immortality of cancer cells, participating in the homologous recombination pathway in the DNA double-strand breaks (DSB) repair^7^. Misregulation of HJURP could induce chromosome instability^12^, which may cause cancer progression^13^. Further, HJURP was over-expressed and correlated with poor prognosis in many malignancies such as lung cancer^7^,^14^, breast cancer^15^, hepatocellular carcinoma^16^,^17^, colorectal cancer^18^ and pancreatic cancer^19^. In breast cancer, HJURP could predict the sensitivity to radiotherapy and served as an independent prognostic marker^15^,^20^. HJURP knockdown could increase radiation-induced cell death of glioblastoma cells^21^. Meanwhile, HJURP protein level would increase in non-small cell lung cancer when cells were exposed to DNA-damaging agents such as γ-irradiation and cisplatin^7^. All of the evidence implied that HJURP probably participated in DNA-damage repair. Abnormal DNA-damage repair was one of possible mechanisms of chemoresistance^22^. According to Filipescu D, et al.'s study, the outcome of HJURP depletion depends on p53 status, and loss of HJURP could induce severe aneuploidy and apoptotic cell death in p53-null transformed cells^23^. Interestingly, HJURP is one of hub genes analyzed from top 30 differentially up-regulated genes in high-grade serous ovarian cancer versus fallopian tube according to our NGS data. HJURP was identified as an independent prognostic biomarker of advanced serous ovarian cancer in Lin L et al.'s study^24^. However, the prognostic value of HJURP in ovarian cancer requires further verification, and the function of HJURP in ovarian cancer still remains unclear.

WEE1 kinase is a G2/M checkpoint which could generate blockade of mitotic entry through phosphorylating cyclin-dependent kinase (CDK) 1 at tyrosine 15 residue^25^,^26^. Dephosphorylated state of CDK1 could activate CDK1/cyclin B complex, facilitating mitotic entrance in G2/M transition^27^,^28^. Cell cycle checkpoints are requisite for genomic integrity and repair of damaged DNA^29^. Dysregulation of G1 checkpoint is widely common in multiple cancers, leading to those more reliant on G2/M checkpoint for DNA repair and tumor cell survival^30^. Inhibition of WEE1 combined with DNA-damaging agents could open the gating of G2/M transition, amplifying DNA damage burden and finally catalyzing mitotic catastrophe^31^,^32^. The synergistic lethality phenomenon indicated WEE1 could be a novel therapeutic target in combinatory strategies containing DNA-damaging agents. Various studies indicated that WEE1 could modulate cisplatin sensitivity by multiple mechanisms and targeting WEE1 was promising for overcoming cisplatin resistance^33^-^35^. Besides, WEE1 could also inactivate CDK2 during S phase, performing maintenance of replication forks and controlling genomic stability^36^-^38^. WEE1 inhibition could result in replication fork stalling and double-strand breaks^39^. AZD1775, a small molecule inhibitor of WEE1, has shown promising antitumor effects in various preclinical studies^36^,^37^. Combined treatment of platinum and AZD1775 has been widely reported to exert synergistic effect against malignancies^40^-^42^. Leijen S, et al. firstly reported that AZD1775 enhanced carboplatin efficacy in TP53-mutated refractory or resistant ovarian cancer in NCT01164995 clinical study^43^. Adavosertib plus gemcitabine strategy had a better progression-free survival(PFS) than single gemcitabine treatment with placebo in refractory or resistant recurrent ovarian cancer according to NCT02151292 clinical study^44^. The present investigation exhibited the capacity of AZD1775 to be a novel targeted agent. Interestingly, we found that WEE1 was affected by HJURP in the present study. However, the detailed mechanism was not fully elucidated, and whether HJURP could modulate AZD1775 sensitivity was worthy of exploration in ovarian cancer.

In the present study, the expression and prognostic value of HJURP was detected in ovarian cancer. Besides, the function of HJURP in proliferation and metastasis was explored in ovarian cancer both in vitro and in vivo. Enrichment analysis based on NGS displayed WEE1 was down-regulated by silencing HJURP. Mechanism research revealed that HJURP could regulate MYC/WEE1 axis and MYC was an upstream transcription factor of WEE1 promoter. Furthermore, we found that silencing HJURP could modulate cisplatin sensitivity of ovarian cancer via MYC/WEE1 axis. The function of HJURP in AZD1775 single agent or combinatory therapy was also detected. Our studies firstly illustrated the function of HJURP in malignant progression and cisplatin/AZD1775 sensitivity of ovarian cancer, indicating HJURP to be a potential target in combinatory therapeutic strategies.

## Materials and methods

### Clinical specimens

All clinical specimens were obtained from specimen bank of Laboratory of Gynecologic Oncology, Qilu Hospital of Shandong University. Ovarian cancer tissues (n=156) for tissue microarrays (TMAs) were from primary surgical operations without prior neoadjuvant therapy during 2007-2013. Fallopian tube tissues were from patients undergoing salpingectomy because of benign diseases. TMAs were manufactured by professional technicians in our laboratory. The research was approved by the Ethics Committee of Shandong University. Written informed consents were received from all participants.

### High-Throughput Differential Gene Expression Analysis

Samples were smashed in TRIzol reagent for total RNA extraction. Sequencing libraries generation and high-throughput RNA-sequence experiments were conducted by Novogene(Beijing, China). Libraries were generated using NEBNext® UltraTM RNA Library Prep Kit for Illumina® (NEB, USA). The clustering of libraries was performed on Illumina sequencing following manufacturer's instructions. Raw data were transformed to reads using CASAVA. Clean reads were aligned to reference genome using Hisat2v2.0.5. Reads' number counting and FPKM calculation was accomplished using featureCounts v1.5.0-p3. Differential expression analysis was performed using the DESeq2 R package (1.16.1). All RNA sequence data had been uploaded to GEO database. Series record was GSE190688 for high-grade serous ovarian cancer and fallopian tube. Series record was GSE190568 for RNA sequencing data of SKOV3 cell line with or without HJURP silencing.

### IHC

Slides with ovarian cancer tissues were sectioned from paraffin-embedded TMAs. All slides received heat mediated Tris/EDTA buffer (pH 8.0) antigen retrieval and were incubated with primary antibody at 4°C overnight. DAB (ZSGB-BIO, China) detection system was conducted, and nucleus was stained by haematoxylin. The staining scores of every case were determined by range and intensity. Detailed procedure of scoring was described in previous study^45^. Scoring work was completed by two professional pathologists independently. Less than 4 was defined low expression, and more than or equal to 5 was high expression.

### Cell culture and cell lines

The SKOV3 and HEK293T cell lines were respectively purchased from the American Type Culture Collection and the Chinese Academy of Sciences. The A2780 and HEY cell lines were friendly presented from Dr. Wei's laboratory(Department of Gynecology and Obstetrics, Northwestern University, Feinberg School of Medicine). All cells were placed in standard cell culture conditions (37°C, 5% CO_2_). SKOV3 and A2780 cell lines were grown in RPMI 1640 medium (Gibco, Thermo Fisher Scientific, USA) containing 10% fetal bovine serum (FBS) (Gemini Bio-Products, USA). HEY and HEK293T cell lines were grown in DMEM medium (Gibco, Thermo Fisher Scientific, USA) containing 10% FBS.

### RNA isolation and qRT-PCR

Total RNA was extracted using TRIzol reagent(Invitrogen, Thermo Fisher Scientific, USA) and then reverse-transcribed to synthesize cDNA using PrimeScript RT Reagent kit(Takara, Japan) according to manufacturers' instructions. The cDNA was amplified on the Real-Time PCR System (QuantStudio3, Thermo Fisher Scientific, USA) with SYBR Premix Ex Taq(Takara, Japan) fluorescence detection for Ct value. ACTB were used as the internal controls for normalization. The relative expression was calculated by 2^-ΔΔCt^ method compared with group control. Synthesis of primers was entrusted to Sangon Biotech (Shanghai, China). All primer sequences are listed in **Supplementary Table S1**.

### WB

Total proteins were extracted using RIPA buffer (Beyotime, China). Protein samples were separated in SDS-PAGE gel and transferred to 0.22µm PVDF membranes (Merck MilliPore, USA). Membranes were incubated with primary antibodies at 4°C overnight after blocking with 5% skim milk. On the next day, membranes were incubated with corresponding secondary antibodies, followed by protein signal visualization using an enhanced chemiluminescence detection kit (PerkinElmer, USA). Protein bands were analyzed using the Image J v1.8.0 software. Detailed information about primary and secondary antibodies was presented in **Supplementary Table S2**.

### siRNA transient transfection

Small interfering RNA (siRNA) and negative control (NC) was synthesized by GenePharma (Shanghai, China). Transfection of siRNA was conducted using Lipofectamine 2000 (Invitrogen, Thermo Fisher Scientific, USA). The oligonucleotide sequences of siRNA used in our study were as follows: siNC sense 5'-UUCUCCGAACGUGUCACGU-3', siNC antisense 5'-ACGUGACACGUUCGGAGAA-3'; siHJURP1 sense 5'-CAGGCUGAGUUUACCUUCCAGCAAA-3', siHJURP1 antisense 5'-UUUGCUGGAAGGUAAACUCAGCCUG-3'; siHJURP2 sense 5'-AGUCGUAUCUCCAGAAAGA-3', siHJURP2 antisense 5'-UCUUUCUGGAGAUACGACU-3'; siMYC sense 5'-ACGGAACUUGUGCGUAA-3', siMYC antisense 5'-UUACGCACAAGUUCCGU-3'; siWEE1 sense 5'-GGGCAUGUAACAAGGAUCU-3', siWEE1 antisense 5'-AGAUCCUUGUUACAUGCCC-3'.

### Plasmid construction

The coding DNA sequence of HJURP was obtained from pENTER plasmid (ViGene Bio, CH826769, China) and ligated into pLenti-C-Myc-DDK-IRES-Puro (pCMV) plasmid (OriGene, USA). The shRNA targeting HJURP (shHJURP) primer was synthesized by Sangon Biotech (Shanghai, China) and cloned to pLKO.1-Puro plasmid (Addgene, USA). Plasmids were amplified in competent escherichia coli and extracted from escherichia coli using Endo-Free Plasmid Midi Kit (Omega Bio-tek, USA). Constructed plasmids were stored at -20°C for lentivirus package preparation. The sequence of shHJURP was as follows: sense 5'-CCTCGAAGTATTCTTCCTTGA-3', antisense 5'-TCAAGGAAGAATACTTCGAGGT-3'.

### Lentivirus package and infection

Lentivirus was packaged in next-generation packaging system using HEK293T cell lines. Constructed plasmid with LTR element, psPAX2 (Addgene, USA) and pMD2.G (Addgene, USA) vectors cotransfected HEK293T cell lines to produce corresponding lentivirus. 2×10^5^ cells/well were seeded on 6-well plates and incubated with lentivirus for 24 hours. Subsequently, 2-4µg/ml puromycin (Merck Millipore, USA) was used for 7-10 days' selection to obtain stable infected cells. Polybrene (Sigma-Aldrich, USA) was applied to enhance efficiency of infection.

### Proliferation and cell viability assay

To describe cell growth curves, 800-1000 cells/well were seeded into six 96-well plates and cultured for 0-5 days. The 3-[4,5-dimethylthiazol-2-yl]-2,5-diphenyl tetrazolium bromide (MTT) assay was conducted to detect the absorbance of every well. Briefly, 20µl MTT reagent (5mg/ml, Sigma-Aldrich, USA) was added to each well for 3 hours incubation at 37°C. Afterwards, the supernatant was replaced by 100µl DMSO (Sigma-Aldrich, USA), and the absorbance value of each well was detected at 490nm by microplate reader(Thermo Fisher Scientific, USA).

### Transwell assays

Matrigel (BD Biosciences, USA) was coated on upper chamber in advance for invasion assay only. A total of 1×10^5^ cells in cell suspension without FBS were seeded into the upper transwell chamber (8µm-pore size, FALCON, USA), and medium containing 20% FBS was added to the lower chamber. After incubation for an appropriate time period, cells in the upper chamber were carefully removed with a cotton swab. Then, chambers containing cells in the lower surface were fixed with 100% methanol and stained with 0.1% crystal violet solution (Sigma-Aldrich, USA). Images were captured using inverted optical-microscope (Olympus, Japan).

### Flow cytometry of cell cycle and apoptosis

Cell cycle phase distribution was detected using Cell Cycle Staining Kit (Multi Sciences, China). Cells were harvested and permeabilized for propidium iodide (PI) staining and analyzed using flow cytometer(BD Biosciences, USA). As for apoptosis assay, APC-Annexin V Apoptosis Detection Kit with 7-AAD (Biolegend, Beijing, China) or FITC-Annexin V Apoptosis Detection Kit with PI(BD Biosciences, USA) was used for early and late apoptotic cells labeling. Labeled samples were detected by flow cytometer (BD Biosciences, USA). All results generated by flow cytometer were analyzed using Flowjo10.4 software.

### Xenograft assay of nude mice

Nude mice (BALB/c; female; 4-week-old) were purchased from Gempharmatech Company (Jiangsu, China), and fed in specific‑pathogen‑free(SPF) condition at Animal Center Laboratory of Shandong University. After one-week acclimatization, cell suspension containing 5×10^6^ tumor cells was injected subcutaneously into both axillas of every nude mouse. Mice were sacrificed 18 days later after injection for measurement and weight of tumor masses. All experiments complied with guidelines and policies of Animal Care and Use Committee, Shandong University.

### Bioinformatics analysis

GEPIA database (https://gepia.cancer-pku.cn/) was used to evaluate the expression of HJURP based on The Cancer Genome Atlas (TCGA) and The Genotype-Tissue Expression (GTEx) data^46^. Protein-protein interaction (PPI) network was established by STRING (Version 11.0)^47^. Afterwards, PPI network was disposed and hub gene was screened by algorithms, which were conducted by Cytohubba in Cytoscape 3.8.2 software. Gene Ontology (GO) and Kyoto Encyclopedia of Genes and Genomes (KEGG) analysis was implemented by the cluster Profiler R package using R version 4.0.3 software. GO annotations included cellular component (CC), molecular function (MF) and biological process (BP).

### Luciferase activity assay

Wild-type WEE1 promoter was synthesized and cloned into pGL3-basic vector (Promega, USA). Binding site was predicted by JASPAR database and site-mutant vector was synthesized by ViGene Bio company. Cells were seeded in 12 well plate and co-transfected with luciferase vector and MYC/Control plasmid. Renilla luciferase pRL-TK reporter vector (Promega, USA) was used as a normalization. Transfected cells were transferred to 96 well plate 24 hours later for luciferase activity detection with Dual-Glo® Luciferase Assay System kit (Promega, USA).

### 5-ethynyl-2'-deoxyuridine(EdU) assay

EdU assay was conducted using BeyoClick^TM^ EdU-488 Cell Proliferation Kit (Beyotime, China). Transfected cells were cultured in medium containing 3μg/ml cisplatin (Sigma-Aldrich, USA). EdU labeling was conducted at the appropriate treated time. Briefly, cells were exposed to 10μM EdU for 2 hours at 37°C. Afterwards, cells were fixed with 4% paraformaldehyde followed by permeabilized with 0.3% Triton X-100 (Solarbio, China, diluted by 1×PBS). Click reaction solution was added to wells for 30 minutes' incubation at room temperature in the dark. Subsequently, cells were stained with Hoechst 33342 to label nucleus. Images were captured using inverted fluorescence microscope (Olympus, Japan). EdU-positive cells' counting and merged photographs' generation was completed using Photoshop CC 2019 software.

### Drug treatment assay

Cisplatin (Sigma-Aldrich, USA) was dissolved with 1×PBS (0.01M, pH 7.2) to get 2mg/ml concentration for storage. AZD1775 (Selleck, S1525, China) was dissolved with DMSO (Sigma-Aldrich, USA) to get 100μM concentration for storage. As for cell viability assay, 4×10^3^ cells/well were seeded into 96-well plate and treated with gradient concentration of cisplatin or AZD1775. Absorbance was detected via MTT assay after drug treatment. Horizontal axis of drug concentration was transformed using log10 algorithm, and half maximal inhibitory concentration (IC50) was calculated using nonlinear regression equation by GraphPad Prism 7.0 software. As for apoptosis assay with drug treatment, 3μg/ml cisplatin or 500nM AZD1775 was incubated for an appropriate period, and then both adherent and suspended cells were wholly harvested for apoptosis assay using flow cytometry.

### Clonogenic formation assay

A total of 4×10^3^ cells/well were seeded in 6 well plates. Indicated concentration of cisplatin or AZD1775 was added to medium once a day 24 hours later. After 7 days' incubation, cells were fixed with methanol and stained with 0.5% crystal violet. Colonies were counted and colony survival rate was calculated by the comparison with control group without any drugs.

### Immunofluorescence

Control and siHJURP cells were treated with cisplatin (2μg/ml) for 8 hours. Resolution of γH2A.X was detected after cisplatin removal for 0, 6, 12, 24 hours. Cells were fixed with 4% paraformaldehyde and permeated with 0.3% Triton-X 100(Solarbio, China). Primary antibody of γH2A.X (Proteintech, 10856-1-AP, China) was incubated in 4°C overnight, and coralite488-conjugated secondary antibody (Proteintech, SA00013-2, China) was incubated in dark for 1 hour. DAPI (Beyotime, China) was used to label nucleus.

### Statistical analysis

All experiments were repeated in triplicates, and the quantitative data are described as mean±SD. Comparison for quantitative data was performed by student t test or one-way ANOVA. Comparison of proportion in fourfold tables was performed with chi-square test. The distributional difference of clinicopathological parameters between HJURP high and low expression group was corrected by 1:1 propensity score matching. Hazard Ratio was calculated by univariate and multivariate Cox regression. Survival analysis was performed using Kaplan-Meier method and log-rank test. Statistical analysis was performed by GraphPad Prism 7.0 and IBM SPSS statistics 23.0 software. All images were generated by GraphPad Prism 7.0 or Photoshop CC 2019 software. *P* value<0.05 was considered to be of statistical significance. ^ns^ represents no significance. ^*^ represents *P*<0.05, ^**^ represents *P*<0.01 and ^***^ represents* P*<0.001.

## Results

### HJURP is over-expressed in serous ovarian cancer and correlates with poor prognosis

**Figure 1:**
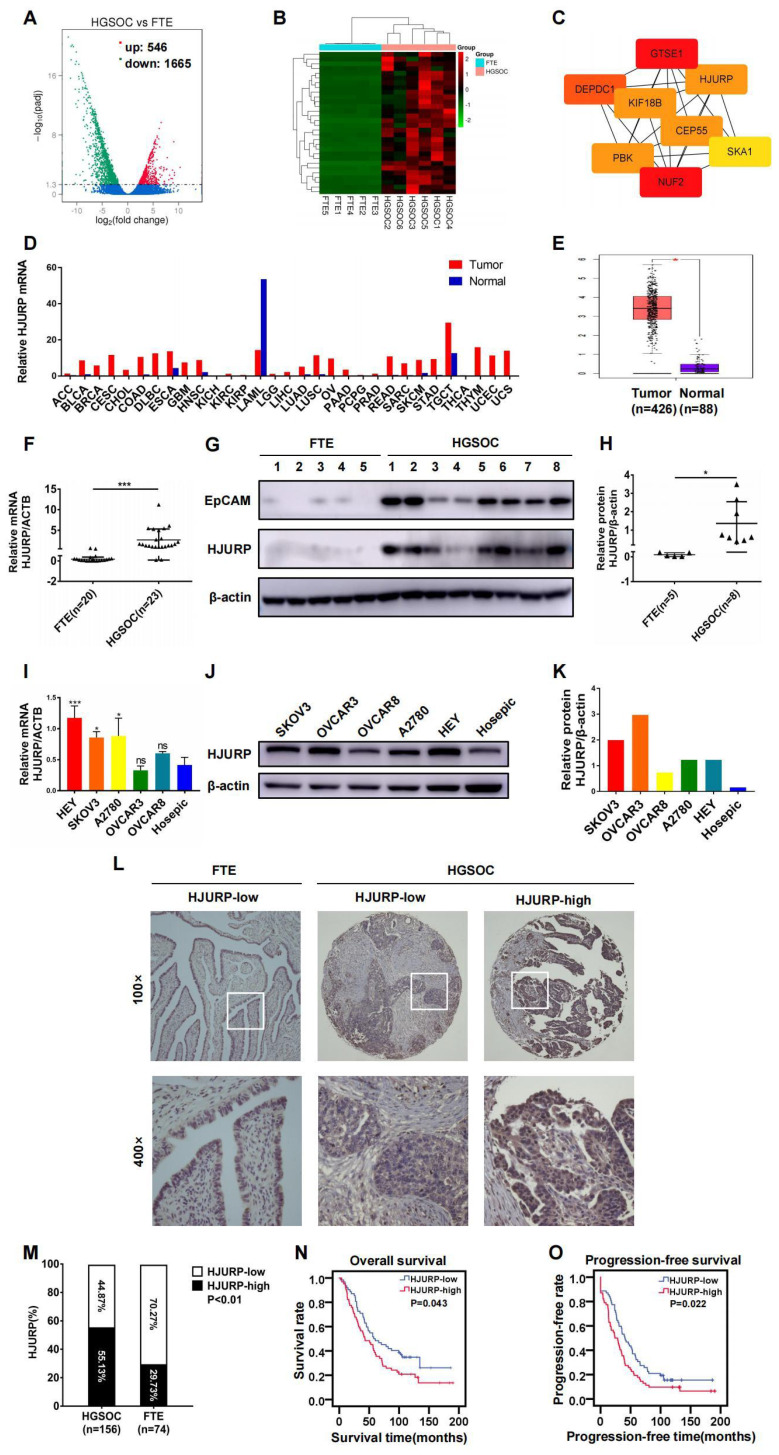
**HJURP was over-expressed and correlated with poor prognosis in ovarian cancer. (A)** Volcano plot displayed DEGs in high-grade serous cancer versus fallopian tube. **(B)** Heatmap illustrated top 30 up-regulated genes of ovarian cancer tissues in DEGs of **Figure 1A**.** (C)** Hub genes were screened from top 30 up-regulated DEGs of **Figure 1B**. **(D)** The mRNA of HJURP expression was shown in multiple tumors compared with normal control in GEPIA database. **(E)** The mRNA level of HJURP was shown in ovarian cancer versus normal ovaries according to GEPIA database. **(F)** Relative mRNA of HJURP expression was shown in ovarian cancer (n=23) and fallopian tube (n=20) tissues. **(G)** Protein level of HJURP was detected by WB. EpCAM was used as a positive marker for epithelial carcinoma, and β-actin was used as loading control. **(H)** Relative quantification of HJURP protein level of **Figure 1G** was plotted in scatter diagram. **(I)** Relative mRNA of HJURP level in different ovarian cancer cell lines was detected by qRT-PCR. **(J)** The HJURP protein level of different ovarian cancer cell lines was detected by WB. **(K)** Relative quantification of HJURP protein level of **Figure 1J** was plotted in histogram. **(L)** Representative images of IHC for low/high HJURP expression were shown in upper row for 100× and in lower row for 400×. **(M)** Percentage of high HJURP expression in both ovarian cancer and fallopian tube was shown in histogram. **(N-O)** OS and PFS curves were plotted in ovarian cancer patients with high/low HJURP expression. (Quantitative data were described as mean±SD, ^ns^ *P*>0.05, ^*^ *P*<0.05, ^**^ *P*<0.01 and ^***^ *P*<0.001).

Based on the viewpoint that some high-grade serous ovarian cancer could originate from fallopian tube^48^, we analyzed NGS data of mRNA between high-grade serous ovarian cancer (n=6) and fallopian tube tissues (n=5) in search of essential genes during tumor progression. Totally, 2211 differentially expressed genes (DEGs) were illustrated in volcano plot, and 546 genes were up-regulated and 1665 genes were down-regulated (**Figure 1A**). Then, top 30 up-regulated genes in ovarian cancer were selected for the next screening (**Figure 1B**). Hub genes were calculated by cytoscape software based on the PPI network constructed by STRING database (**Supplementary Figures S1A, B** and **Supplementary Table S3**). Eight genes (NUF2, GTSE1, DEPDC1, KIF18B, PBK, CEP55, HJURP, SKA1) were potential hub genes correlated to ovarian cancer progression (**Figure 1C**). In view of the probable role of HJURP in chromosomal stability and tumor progression that we have expounded above, HJURP was further focused on in the present study. As shown in **Figures 1D, E**, HJURP was over-expressed in majorities of malignancies including ovarian cancer according to GEPIA database. Then, the expression of HJURP was further evaluated using our own tumor specimens and cell lines. The mRNA and protein levels of HJURP were significantly higher in ovarian cancer than those in fallopian tube, performed by qRT-PCR and WB (**Figures 1F-H**). Besides, HJURP was over-expressed in HEY, SKOV3 and A2780 ovarian cancer cell lines compared with Hosepic control cell line (**Figures 1I-K**).

Subsequently, we manufactured a TMA containing 156 ovarian cancer cases to form a retrospective cohort. IHC was performed in the TMA slides and representative high/low expression fields of HJURP were presented in **Figure 1L**. All cases were divided into two groups based on HJURP expression score. The percentage of over-expressed HJURP in ovarian cancer (55.13%, 86/156) was more than that in fallopian tube (29.73%, 22/74) (**Figure 1M**). As shown in **Table 1**, four clinicopathological parameters including serum carbohydrate antigen 125 (CA125) level (*P*=0.004), ascites volume (*P*=0.000), FIGO stage (*P*=0.009) and omentum/peritoneum metastasis (*P*=0.009) were disproportionately distributed in high/low HJURP expression groups, implying HJURP expression may be associated with these clinical characteristics. All parameters above were symbol of advanced or progressive phenomenon of ovarian cancer, indicating that HJURP may participate in the malignant progression. To alleviate the effects of confounding bias in survival analysis, we performed 1:1 propensity score matching to parallel the baseline level of high/low HJURP expression group for further analysis (**Table 2**). Univariate and multivariate Cox regression revealed that omentum/peritoneum metastasis was an independent prognostic marker for overall survival (OS) (Hazard Ratio [95%CI]: 2.480 [1.477-4.164], *P*=0.001) and progression-free survival (PFS) (Hazard Ratio [95%CI]: 2.589 [1.620-4.139], *P*=0.000) (**Table 3 and Table 4**). High HJURP expression was a risk factor for PFS (Hazard Ratio [95%CI]: 1.453 [0.995-2.123], *P*=0.053) (**Table 4**). Kaplan-Meier analysis indicated that high HJURP expression had a worse prognosis in ovarian cancer patients, evaluated by OS (*P*=0.043) and PFS (*P*=0.022) (**Figures 1N, O**). All findings displayed that HJURP was over-expressed in ovarian cancer and correlated with poor prognosis, implying HJURP may play a key role in ovarian cancer progression.

### HJURP promotes malignant biological behaviors of ovarian cancer

**Figure 2:**
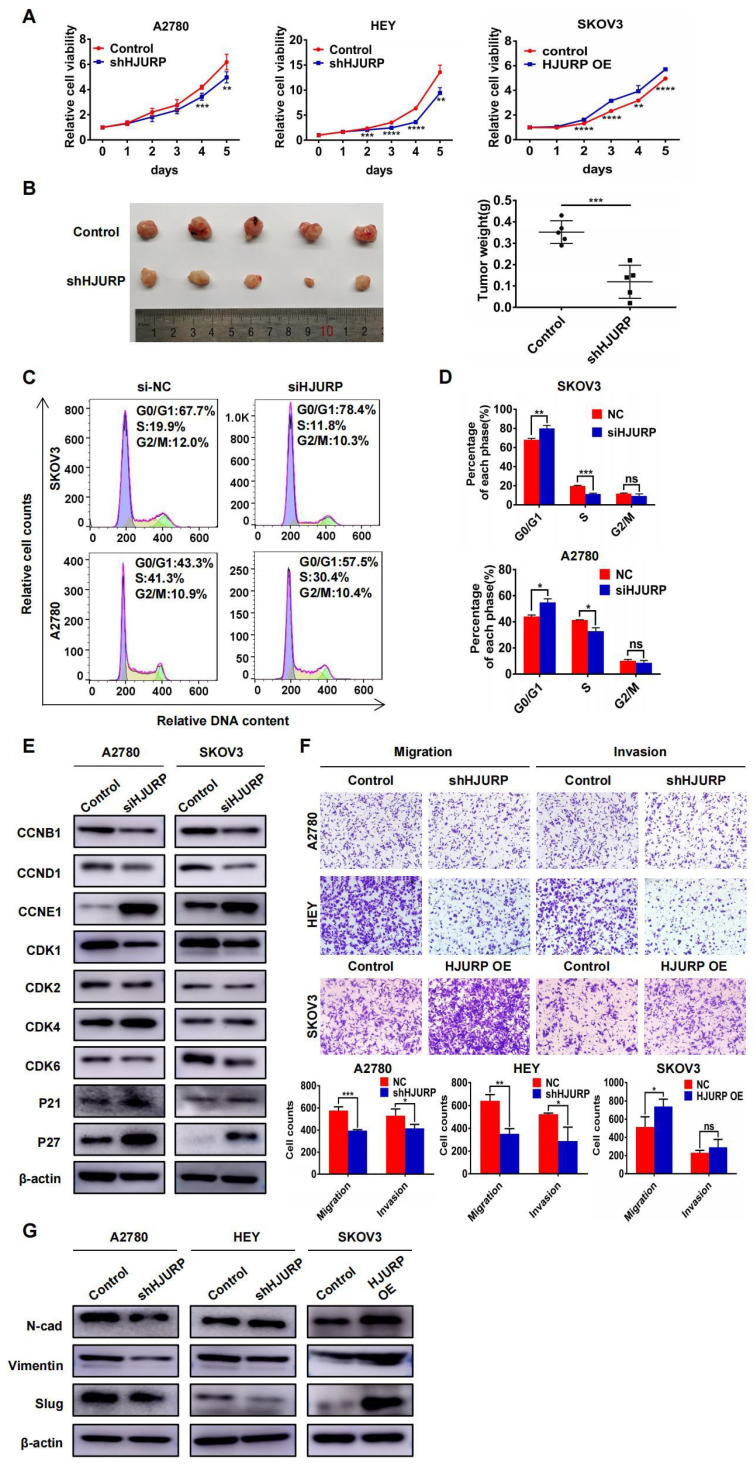
** HJURP promoted proliferation and malignant progression of ovarian cancer in vitro and in vivo. (A)** MTT assay was performed to detect the proliferative effect of down-regulated HJURP in A2780 and HEY and up-regulated HJURP in SKOV3. **(B)** Xenograft assay was performed in nude mice. The tumor images of control and shHJURP group were captured and put in the left, and scatter plot of tumor weight was laid in the right. **(C)** Cell cycle distribution was analyzed by flow cytometry. Purple area represented G0/G1 phase. Yellow area represented S phase, and green area represented G2/M phase. **(D)** The proportion of each cell cycle phase was illustrated in histogram. **(E)** Cell cycle-associated markers were detected by WB in A2780 and SKOV3 with or without siHJURP. **(F)** Images of transwell assays for migration and invasion were captured (200×). Cell counts of transwell assays were illustrated in histogram below transwell images. **(G)** EMT markers were detected by WB. (Quantitative data are described as mean±SD, ^ns^ *P*>0.05, ^*^ *P*<0.05, ^**^ *P*<0.01 and ^***^ *P*<0.001).

To explore the biological function of HJURP in tumor progression, stable cell lines with HJURP knockdown or overexpression were established. As shown in **Supplementary Figure S2A**, we designed two silencing oligos against HJURP. However, siHJURP2 oligo could only cut off less than 40% of HJURP expression compared with control in both A2780 and SKOV3. Consequently, we selected siHJURP1 oligo for following experiments. The siHJURP1 could inhibit HJURP expression efficiently both in mRNA and protein level (**Supplementary Figures S2A, B**). The efficiency of stable transfected cell lines was also verified both in mRNA and protein level (**Supplementary Figures S2C-E**). As shown in **Figure 2A**, HJURP knockdown in A2780 and HEY could inhibit cell proliferation especially in the last 2-3 days, and HJURP overexpression in SKOV3 could increase cell growth. To further investigate the function of HJURP in carcinogenesis and tumor growth, tumor subcutaneous formation assay was performed in nude mice and the results were displayed in **Figure 2B** and** Supplementary Figures S2F, G**. The volume and weight of tumor mass was significantly diminished in shHJURP group compared with negative control, but the effect of HJURP overexpression on tumor growth was not conspicuous in vivo like that of HJURP knockdown. Besides, cell cycle analysis by flow cytometry illustrated that silencing HJURP could increase the cell proportion of G1 phase and induce G0/G1 arrest (**Figures 2C, D**). Subsequently, cell cycle-associated proteins were detected using WB (**Figure 2E**). Consistent with the phenomenon of G0/G1 arrest, cell cycle proteins related to early G1 progressive regulation such as CCND1 and CDK6 were down-regulated, and CDK suppressor P21 and P27 was up-regulated after silencing HJURP. There were no significant alterations of CDK1, CDK2 and CCNB1, which mainly participated in S and G2/M phase regulation. However, CCNE1, mainly functioning in late G1 phase and G1/S transition, was up-regulated upon silencing HJURP. We speculated it a compensatory effect accompanied with early G1 stagnation induced by silencing HJURP. Literally, silencing HJURP inhibited cell growth probably through early G0/G1 phase arrest.

Furthermore, transwell assays were performed to detect whether HJURP could affect metastatic process. As shown in **Figure 2F**, migration and invasion capacity was obviously attenuated by HJURP knockdown in A2780 and HEY. However, HJURP overexpression could only enhance migration capacity in SKOV3. To investigate whether HJURP was associated with epithelial-mesenchymal transition (EMT), some EMT markers related to mesenchymal phenotype were assessed via WB. As shown in **Figure 2G,** Vimentin and Slug were down-regulated after HJURP knockdown, and Slug was up-regulated obviously when HJURP was over-expressed in SKOV3. Collectively, HJURP could promote ovarian cancer progression, and all findings had provided foundation for subsequent studies about HJURP downstream-regulated networks and potential value of silencing HJURP in ovarian cancer therapy.

### High-throughput sequencing and PPI network analysis reveals WEE1 is regulated by HJURP

**Figure 3:**
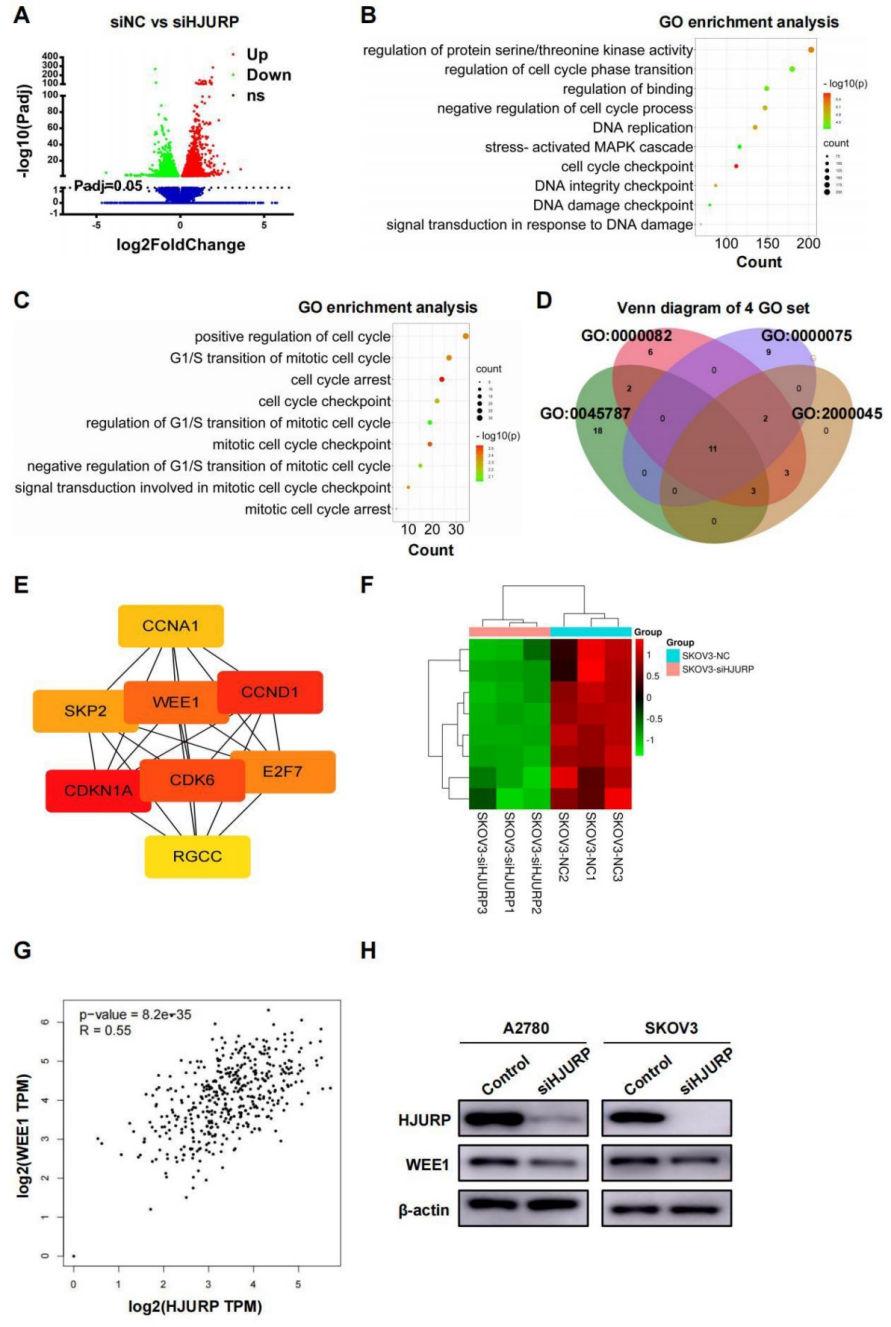
** NGS data of SKOV3 treated with siNC and siHJURP was analyzed in search of regulatory mechanism networks. (A)** Volcano plot displayed DEGs of siNC versus siHJURP.** (B)** GO enrichment of all DEGs in **Figure 3A** was analyzed and illustrated. The size of every sphere represented the level of gene counts enriched in the corresponding pathway. The color of every sphere represented the relative -log_10_ (P-adj.) level. **(C)** GO terms related to cell cycle regulation with adjusted threshold (P-adj.<0.05 and log_2_FoldChange>0.5) was picked out for exhibition. The meaning of legends was the same as that in **Figure 3B**. **(D)** Venn diagram was plotted based on genes from selected GO terms in **Figure 3C**. **(E)** Hub genes were screened from the union set of **Figure 3D**. **(F)** Heatmap was plotted with 8 hub genes based on NGS data. **(G)** Correlation of HJURP and WEE1 expression was analyzed based on ovarian cancer data in GEPIA database. **(H)** WEE1 expression was detected by WB after HJURP silencing. (Quantitative data are described as mean±SD, ^ns^ *P*>0.05, ^*^ *P*<0.05, ^**^ *P*<0.01 and ^***^ *P*<0.001).

SKOV3 cell lines transfected with siNC and siHJURP were used for NGS assay in search of related process and networks associated with HJURP. Totally, 5296 DEGs under the condition of adjusted P value (P-adj)<0.05 were displayed and listed in **Figure 3A** and **Supplementary Table S4**. GO enrichment analysis of all DEGs revealed that HJURP-regulated gene clusters were mainly associated with cell cycle checkpoint, DNA replication, DNA integrity checkpoint, negative regulation of cell cycle process and signal transduction in response to DNA damage (**Figure 3B** and **Supplementary Table S5**), consistent with previous studies of HJURP in cell cycle and chromosomal stability^7^-^9^,^12^. KEGG enrichment analysis revealed that down-regulated gene clusters after HJURP silencing mainly participated in cell cycle, pathways in cancer, cellular senescence, small cell lung cancer and DNA replication, et al. (**Supplementary Figure S3A** and **Supplementary Table S6**). Subsequently, we controlled threshold at log_2_FoldChange>0.5 and P-adj<0.05 for admission of more remarkable down-regulated genes along with siHJURP, prepared for further GO analysis (**Supplementary Table S7**). Considering the basic role of HJURP in centromere and cell cycle regulation^8^,^9^, we selected genes from several cell cycle-associated GO terms enriched above for exploration of downstream regulated networks (**Figure 3C**). Genes from four set of GO terms (GO: 0045787, GO: 0000082, GO: 0000075, GO: 2000045) associated with cell cycle were plotted in Venn diagram (**Figure 3D**). The union set above was prepared for PPI network construction and hub genes were analyzed by cytoscape (**Supplementary Figures S3B, C** and **Supplementary Table S8**). Eight genes (CDKN1A, CCND1, CDK6, WEE1, E2F7, SKP2, CCNA1 and RGCC) were considered to be hub genes (**Figure 3E**). Heatmap of hub genes was plotted in **Figure 3F** using NGS data. Among hub genes, CCND1 and CDK6 was down-regulated, in line with what we detected above of those in protein level, strongly indicating that early G0/G1 proteins CCND1 and CDK6 were regulated by HJURP in transcriptional expression. It was acknowledged that WEE1 was a G2/M cell cycle checkpoint and allowed time for DNA damage repair^25^,^29^. Inhibition of WEE1 with damaged DNA may induce mitotic catastrophe and apoptosis^31^,^32^. Coincidently, HJURP probably participated in DSB repair of DNA^7^ and regulated WEE1, providing more perspectives of the study between HJURP and WEE1. In addition, HJURP was positively correlated with WEE1 in ovarian cancer according to GEPIA database (**Figure 3G**), and protein level of WEE1 after silencing HJURP was also detected by WB (**Figure 3H**).

### HJURP indirectly regulates WEE1 through the transcription factor MYC

**Figure 4:**
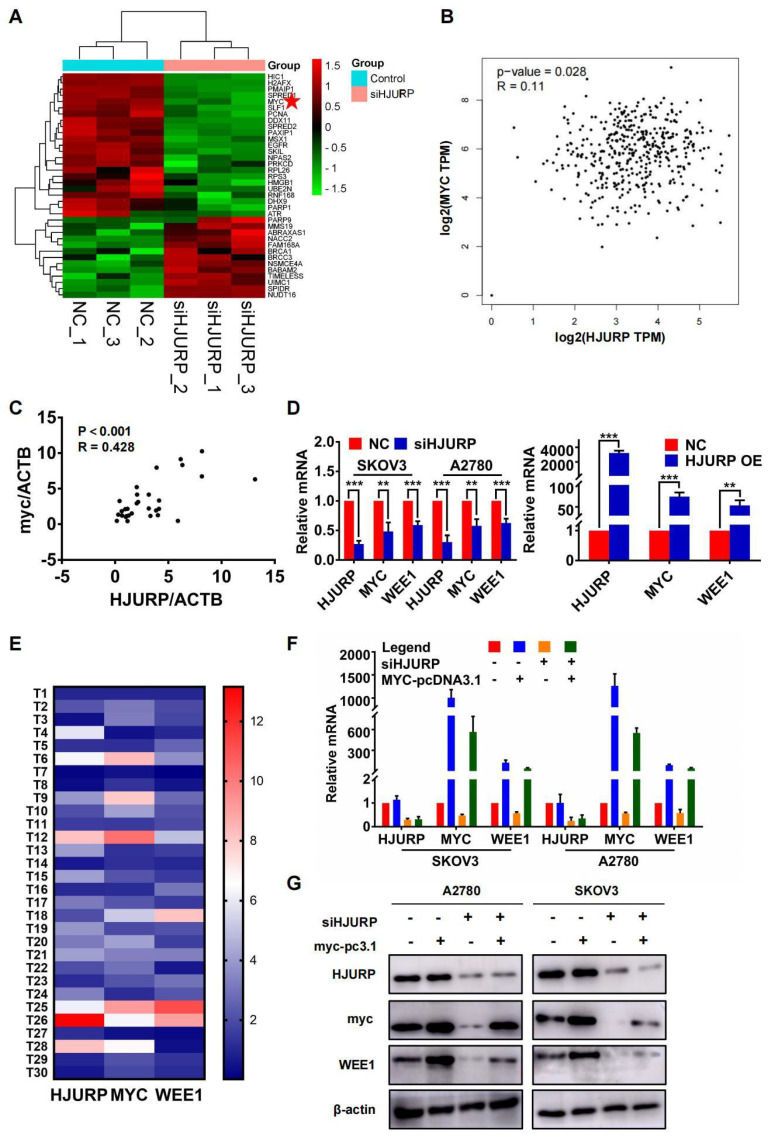
** Silencing HJURP could down-regulate WEE1 via inhibiting MYC expression. (A)** Genes enriched in positive regulation of response to DNA damage stimulus pathway were illustrated in a heatmap based on NGS data, and MYC was marked with a red pentagram.** (B)** Correlation of HJURP and MYC expression was analyzed based on ovarian cancer data in GEPIA database.** (C)** Correlation of HJURP and MYC was illustrated in scatter diagram based on relative mRNA level detected by qRT-PCR in 30 ovarian cancer tissues. R represented Pearson correlation coefficient. **(D)** Relative mRNA level of HJURP, MYC and WEE1 was detected by qRT-PCR. The left histogram illustrated control and siHJURP group in SKOV3 and A2780. The right histogram illustrated control and HJURP overexpression group in SKOV3.** (E)** The expression of HJURP, MYC and WEE1 was detected by qRT-PCR in 30 ovarian cancer tissues, and heatmap was plotted to demonstrate the tendency of consistent expression pattern of the 3 genes. Red cells meant high expression and blue cells meant low expression.** (F-G)** Relative mRNA and protein level of HJURP, MYC and WEE1 was detected by qRT-PCR and WB respectively in SKOV3 and A2780. Down-regulated WEE1 caused by siHJURP was partially rescued by MYC overexpression. (Quantitative data are described as mean±SD, ^ns^ *P*>0.05, ^*^ *P*<0.05, ^**^ *P*<0.01 and ^***^ *P*<0.001).

Considering the response of HJURP in DNA damage induction reported previously and the function of HJURP in DNA repair^7^, ^12^, we selected GO process termed positive regulation of response to DNA damage stimulus for further analysis to explore the intermediate mechanisms of regulation between HJURP and WEE1. Heatmap of genes from the above GO term was plotted in **Figure 4A**. Interestingly, MYC was positively correlated with HJURP both in GEPIA ovarian cancer database and in 30 tumor specimens from our laboratory (**Figures 4B, C**). To demonstrate whether there were regulated association between HJURP, MYC and WEE1, qRT-PCR was performed to detect the expression of MYC and WEE1 after HJURP was silenced or over-expressed. As shown in **Figure 4D**, MYC and WEE1 were obviously down-regulated after silencing HJURP in SKOV3 and A2780. HJURP overexpression could also increase the expression of MYC and WEE1 in SKOV3 (**Figure 4D**). Expression of HJURP, MYC and WEE1 in 30 ovarian cancer specimens was detected by qRT-PCR and relative expression heatmap was plotted in **Figure 4E**. We found that the expression pattern of the 3 genes had the tendency of consistence in every tumor tissue. Subsequently, rescued experiment of HJURP, MYC and WEE1 expression was performed. As shown in **Figures 4F, G**, we found that up-regulated MYC could not affect the expression of HJURP. However, silencing HJURP could down-regulate MYC and WEE1. Interestingly, up-regulating MYC could partially reverse the inhibitory effect of WEE1 caused by silencing HJURP. All results above indicated that HJURP may regulate WEE1 through MYC expression.

**Figure 5:**
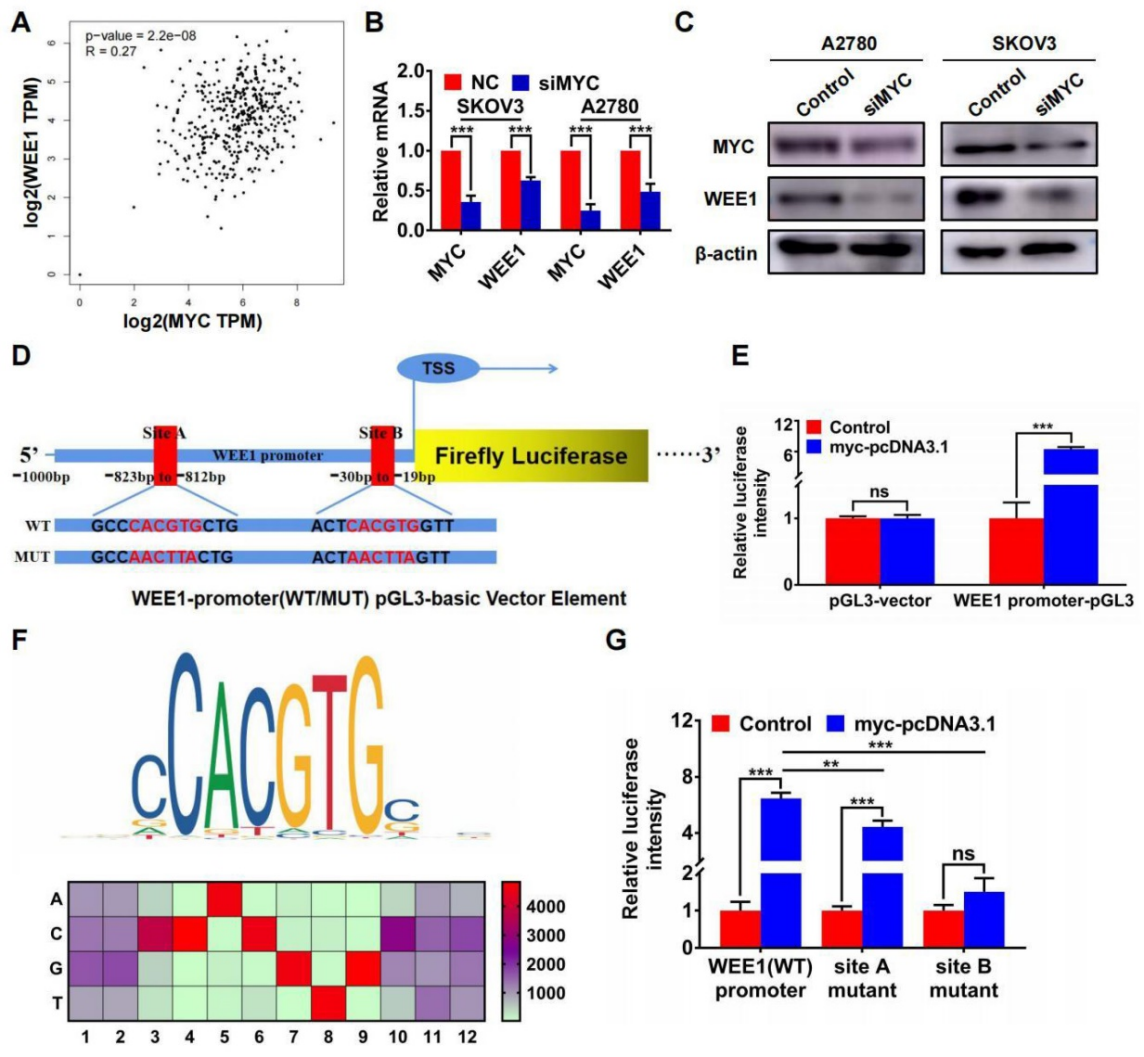
** WEE1 was a downstream target of the transcription factor MYC in ovarian cancer. (A)** Correlation of MYC and WEE1 expression was analyzed based on ovarian cancer data in GEPIA database. **(B)** Relative mRNA level of MYC and WEE1 was detected by qRT-PCR. **(C)** Protein level of MYC and WEE1 was detected by WB. **(D)** The schematic diagram of luciferase assay was illustrated. There were two predicted binding sites in JASPAR. The wild type sequence and corresponding mutant site sequence was listed in WT and MUT box. TSS meant transcription starting site.** (E)** The relative luciferase intensity was demonstrated in histogram. **(F)** The predicted binding sequence in JASPAR was illustrated. The bottom heatmap demonstrated the frequency matrix of every bases(A, C, T, G). **(G)** The relative luciferase intensity treated with or without MYC overexpression was demonstrated in histogram. The left one was performed with luciferase vector cloned with wild type WEE1 promoter. The right two groups were performed with luciferase vector carrying mutant WEE1 promoter in site A and site B respectively. (Quantitative data are described as mean±SD, ^ns^ *P*>0.05, ^*^ *P*<0.05, ^**^ *P*<0.01 and ^***^ *P*<0.001).

Furthermore, we found that MYC was positively correlated with WEE1 in GEPIA ovarian cancer database (**Figure 5A**). Silencing MYC could inhibit the expression of WEE1 both in mRNA and in protein level (**Figures 5B, C**), indicating that WEE1 was regulated by MYC in transcriptional level. Subsequently, we cloned WEE1 promoter into pGL3-basic vector for luciferase activity assay. The schematic luciferase plasmid element was presented in **Figure 5D**. As shown in **Figure 5E**, MYC up-regulation could enhance relative luciferase intensity of WEE1 wild-type promoter. Then, we predicted binding sites of MYC in WEE1 promoter by JASPAR database and found the most frequent binding sequence was CACGTG. The frequency matrix was illustrated in **Figure 5F**. Site A and site B was mutated respectively and WEE1 promoter carrying mutant site was cloned into pGL3-basic vector again for luciferase activity assay (**Figure 5D**). As shown in **Figure 5G**, both mutant site A and mutant site B could lead to decrease of relative luciferase intensity compared with wile-type WEE1 promoter, indicating that MYC could transcriptionally activate WEE1 through both site A and site B. However, in site B mutant group, the increase of luciferase activity caused by MYC up-regulation was of no significance, indicating that site B may be the key binding site of MYC. Collectively, HJURP could modulate WEE1 through MYC and MYC was a transcription factor targeting WEE1 promoter.

### HJURP modulates cisplatin chemoresistance in ovarian cancer through MYC/WEE1 axis

**Figure 6:**
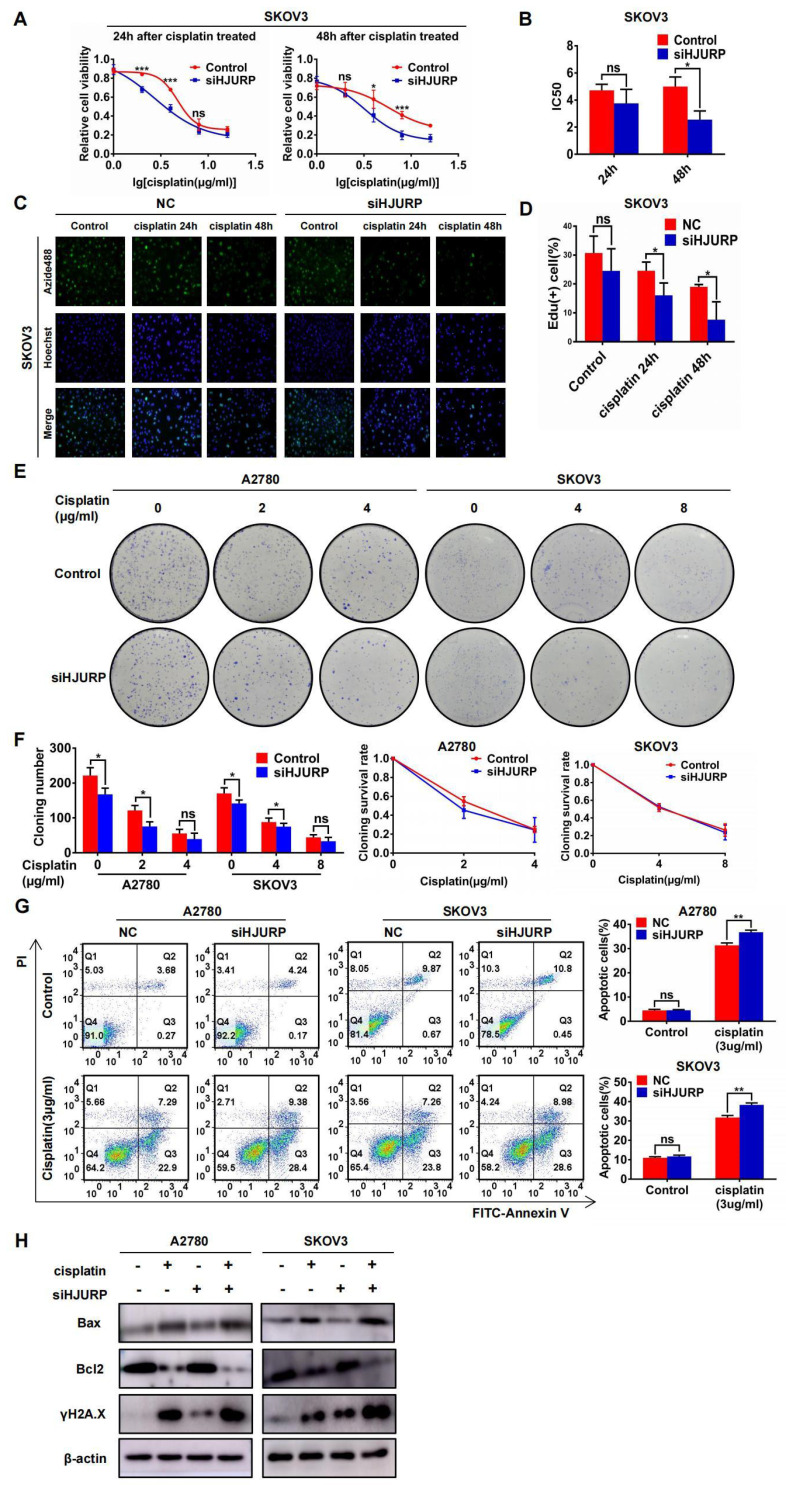
** Silencing HJURP could enhance sensitivity of ovarian cancer cells to cisplatin. (A)** Cell viability of SKOV3 with cisplatin treatment (0, 1, 2, 4, 8, 16μg/ml) was detected by MTT assay. **(B)** IC50 values of cisplatin were calculated using nonlinear regression equation by GraphPad Prism 7.0 software. **(C)** EdU assay was performed in control and siHJURP groups after corresponding cisplatin treatment (200×). Hoechst was used to label nucleus and determine total cell number. **(D)** EdU positive cell proportion was showed in histogram. **(E)** Clonogenic assay was performed in control and siHJURP groups under the gradient treatment of cisplatin in A2780 and SKOV3. **(F)** Cloning formation number of **Figure 6E** was shown in histogram in the left. Cloning survival rate was shown in line chart in the right. **(G)** Apoptosis assay was detected by flow cytometry after cisplatin treatment in A2780 and SKOV3. The cell population images were in the left, and the percentage of apoptotic cells was shown in histogram in the right. **(H)** Bax, Bcl2 and γH2A.X expression was detected by WB. (Quantitative data are described as mean±SD, ^ns^ *P*>0.05, ^*^ *P*<0.05, ^**^ *P*<0.01 and ^***^ *P*<0.001).

To demonstrate the function of HJURP in cisplatin chemoresistance, gradient concentration of cisplatin (0, 1, 2, 4, 8, 16μg/ml) was added to SKOV3 and A2780 for 24 or 48 hours. Cells were transfected with siNC or siHJURP before cisplatin treatment. As shown in **Figures 6A, B** and** Supplementary Figures S4A, B**, cell viability was destructed in a dose-dependent manner, and IC50 of cisplatin was lower in siHJURP group than that in siNC at the appropriate incubation time. To further illustrate whether silencing HJURP could increase sensitivity of cancer cells to cisplatin, EdU proliferation assay was performed. As shown in **Figures 6C, D** and** Supplementary Figures S4C, D**, the percentage of proliferative cells decreased after increasing incubation time of cisplatin (0, 24 and 48 hours), and silencing HJURP could inhibit proliferative cells more compared with control. Clonogenic formation assay was also performed in A2780 and SKOV3 with gradient cisplatin treatment. As shown in **Figures 6E, F**, A2780 was treated with gradient concentration of cisplatin at 0, 2 and 4μg/ml and SKOV3 was treated with that at 0, 4 and 8μg/ml. Cloning number was counted and silencing HJURP could attenuate colony formation at 2μg/ml for A2780 and 4μg/ml for SKOV3(**Figure 6F**). To exclude the influence of siHJURP on proliferation, we calculated cloning survival rate to evaluate the effect of HJURP on chemoresistance. In A2780, cloning survival rate was lower in siHJURP than control at 2μg/ml cisplatin concentration (**Figure 6F**). However, the discrepancy of cloning survival rate was not conspicuous in SKOV3 (**Figure 6F**). As shown in **Supplementary Figures S4E, F**, HJURP overexpression in SKOV3 could enhance clonogenic formation capacity under cisplatin treatment (0, 4 and 8μg/ml), and cloning survival rate was higher in HJURP overexpression group.

Moreover, apoptosis assay performed by flow cytometry demonstrated that silencing HJURP added with cisplatin (3μg/ml) treatment exerted a combined effect. Proportion of apoptotic cells reached a higher level in the combined group than any other single treatment group both in A2780 and SKOV3 (**Figure 6G**). Notably, silencing HJURP only did not lead to apoptosis (**Figure 6G**). Then, markers of apoptosis pathways and DSB were detected by WB. As shown in **Figure 6H**, cisplatin treatment could induce Bax up-regulation and Bcl2 down-regulation, consistent with the apoptosis phenomenon brought about by cisplatin. Silencing HJURP only did not change the expression of Bax and Bcl2 (**Figure 6H**). Moreover, siHJURP could lead to up-regulated γH2A.X, implying that HJURP participated in DNA-damage repair (**Figure 6H**). All results indicated that silencing HJURP could enhance sensitivity of ovarian cancer cells to cisplatin, and combined treatment of cisplatin and HJURP interference could provide a novel strategy against chemoresistance.

We have testified that HJURP could modulate MYC/WEE1 axis and MYC was an upstream transcription factor of WEE1. Considering that WEE1 inhibitor could synergize with many DNA damage agents including cisplatin^31^-^35^, we would like to explore whether MYC/WEE1 axis was the intermediate process of chemoresistance induced by HJURP. As shown in **Supplementary Figures S5A, B**, clonogenic formation number increased in HJURP overexpression group compared with control at 2μg/ml concentration of cisplatin in SKOV3. In A2780, every gradient concentration of cisplatin (0, 2, 4 and 8μg/ml) displayed enhanced capacity of clonogenic formation. Cloning survival rate of HJURP overexpression increased at 2μg/ml concentration of cisplatin in both SKOV3 and A2780 (**Supplementary Figure S5B**). However, all the enhanced clonogenic capacity in HJURP overexpression could be partially reversed by silencing WEE1, indicating that WEE1 was an intermediate mechanism of chemoresistance induced by HJURP. Notably, the reversed effect of siWEE1 was partial especially in SKOV3, implying that MYC/WEE1 axis was not the sole pathway in chemoresistance. Furthermore, apoptosis assay performed by flow cytometry showed that siWEE1 could partially increase percentage of apoptotic cells attenuated by HJURP overexpression, consistent with results in clonogenic formation assay (**Supplementary Figures S5C, D**). All cells were treated with 3μg/ml cisplatin after corresponding transfection in the above apoptosis assay.

**Figure 7:**
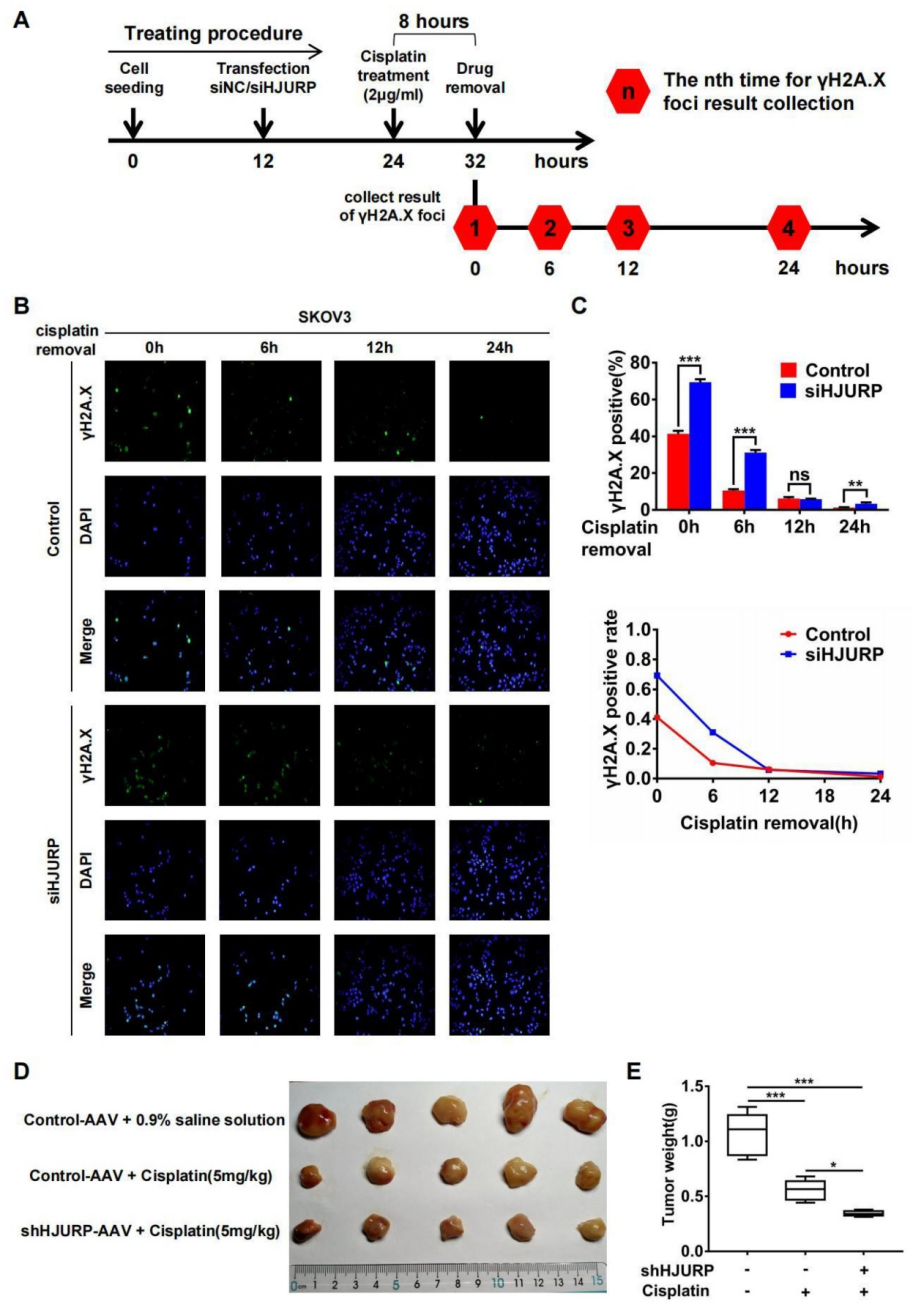
** Silencing HJURP could attenuate DNA repair of cisplatin-induced DNA damage. (A)** Schematic treating procedure was provided to illustrate the measurement of DNA repair of cisplatin-induced DNA damage. **(B)** Immunofluorescence was performed in SKOV3 to detect γH2A.X foci after cisplatin removal for 0, 6, 12 and 24 hours. DAPI was used to label nucleus. **(C)** The percentage of γH2A.X positive foci in **Figure 7B** was illustrated in the upper histogram. The variation trend of γH2A.X positive rate after cisplatin removal was illustrated in line chart at the bottom. **(D)** Xenograft was performed in nude mice and treated with different conditions to illustrate the function of HJURP in cisplatin therapy. Tumor images were captured. **(E)**Tumor weight in **Figure 7D** was shown in box plot. (Quantitative data are described as mean±SD, ^ns^ *P*>0.05, ^*^ *P*<0.05, ^**^ *P*<0.01 and ^***^ *P*<0.001).

Subsequently, immunofluorescence of γH2A.X was performed after cisplatin removal for indicated hours to know if HJURP participated in DNA repair of cisplatin-induced DNA damage. The schematic treating procedure was presented in **Figure 7A**. As shown in **Figures 7B, C**, the percentage of γH2A.X positive foci was decreasing along with cisplatin removal, indicating that cells were going through DNA repair. However, silencing HJURP led to slower speed of γH2A.X decreasing especially during the incipient 6 hours. This phenomenon confirmed that HJURP was necessary for DNA repair of cisplatin-induced DNA damage. Then, to further investigate the combined effect between HJURP knockdown and cisplatin treatment on tumor inhibition, in vivo experiment was performed. As shown in **Figures 7D, E**, cisplatin treatment had an obvious efficacy on tumor growth inhibition, and shHJURP could enhance the inhibitory effect of cisplatin, which further confirmed the application of silencing HJURP for increasing sensitivity of ovarian cancer cells towards cisplatin.

### Silencing HJURP could enhance sensitivity of ovarian cancer cells to AZD1775

**Figure 8:**
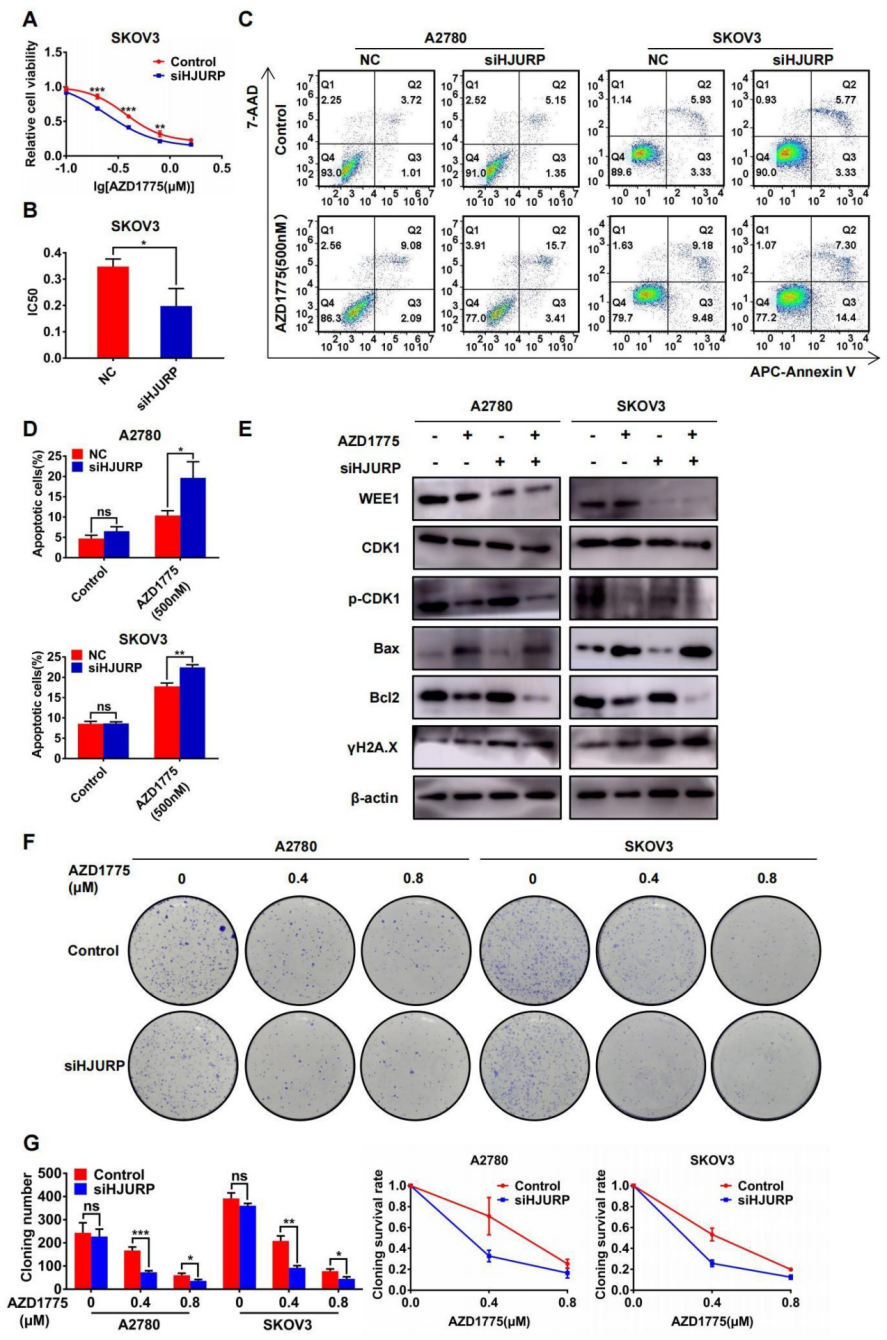
** Silencing HJURP could enhance sensitivity of ovarian cancer cells to AZD1775. (A)** Cell viability of SKOV3 with AZD1775 treatment (0, 0.1, 0.2, 0.4, 0.8, 1.6μM) was detected by MTT assay. **(B)** IC50 values of AZD1775 were calculated using nonlinear regression equation by GraphPad Prism 7.0 software. **(C)** Apoptosis assay was detected by flow cytometry after AZD1775 treatment in A2780 and SKOV3. **(D)** The percentage of apoptotic cells in **Figure 8C** was shown in histogram. **(E)** WEE1, CDK1, p-CDK1, Bax, Bcl2 and γH2A.X was detected by WB to demonstrate the effect of silencing HJURP and AZD1775 treatment in apoptosis and DNA damage.** (F)** Clonogenic assay was performed in control and siHJURP groups under the gradient treatment of AZD1775 in A2780 and SKOV3. **(G)** Cloning formation number of **Figure 8F** was shown in histogram in the left. Cloning survival rate was shown in line chart in the right. (Quantitative data are described as mean±SD, ^ns^ *P*>0.05, ^*^ *P*<0.05, ^**^ *P*<0.01 and ^***^ *P*<0.001).

In view of the antitumor effect of AZD1775 in combined therapy^36^,^37^, the research of AZD1775 in ovarian cancer cells was performed in our study. As shown in **Figures 8A, B**, cell viability assay was performed under the gradient concentration of AZD1775 (0, 0.1, 0.2, 0.4, 0.8, 1.6μM). Cells were transfected with siNC/siHJURP before drug treatment and AZD1775 would be incubated for 72 hours. Cell viability decreased in a dose-dependent manner and silencing HJURP could cause IC50 decline, which indicated that silencing HJURP probably enhanced sensitivity of cancer cells to AZD1775 (**Figures 8A, B**). In addition, apoptosis assay showed that single AZD1775 treatment could increase the percentage of apoptotic cells compared with control, and silencing HJURP plus AZD1775 could reach the highest apoptotic rate (**Figures 8C, D**). Then, WEE1, CDK1, p-CDK1, Bax, Bcl2 and γH2A.X were evaluated by WB in the indicated cells above (**Figure 8E**). Phosphorylation of CDK1 was inhibited by AZD1775 treatment, in agreement with previous studies^25^,^26^. Additionally, both AZD1775 treatment and HJURP silencing could increase the expression of γH2A.X, implying that HJURP and WEE1 might synergistically facilitate DNA-damage repair, and inhibition of both could probably provide opportunities for mitotic catastrophe and cause cell death^31^,^32^. To further verify the function of HJURP in AZD1775 treatment, clonogenic formation assay was conducted subsequently. As shown in **Figures 8F, G**, cloning number and cloning survival rate was obviously attenuated in siHJURP group at 0.4μM concentration of AZD1775 both in A2780 and SKOV3. Besides, HJURP overexpression in SKOV3 could increase cloning number and cloning survival rate at 0.4μM concentration of AZD1775 (**Supplementary Figures S6A, B**). Collectively, silencing HJURP could enhance sensitivity of ovarian cancer cells to AZD1775, and HJURP probably affected cancer response to AZD1775 through mediation of DNA damage repair.

### The combined effect between cisplatin and AZD1775 is subjected to HJURP

**Figure 9:**
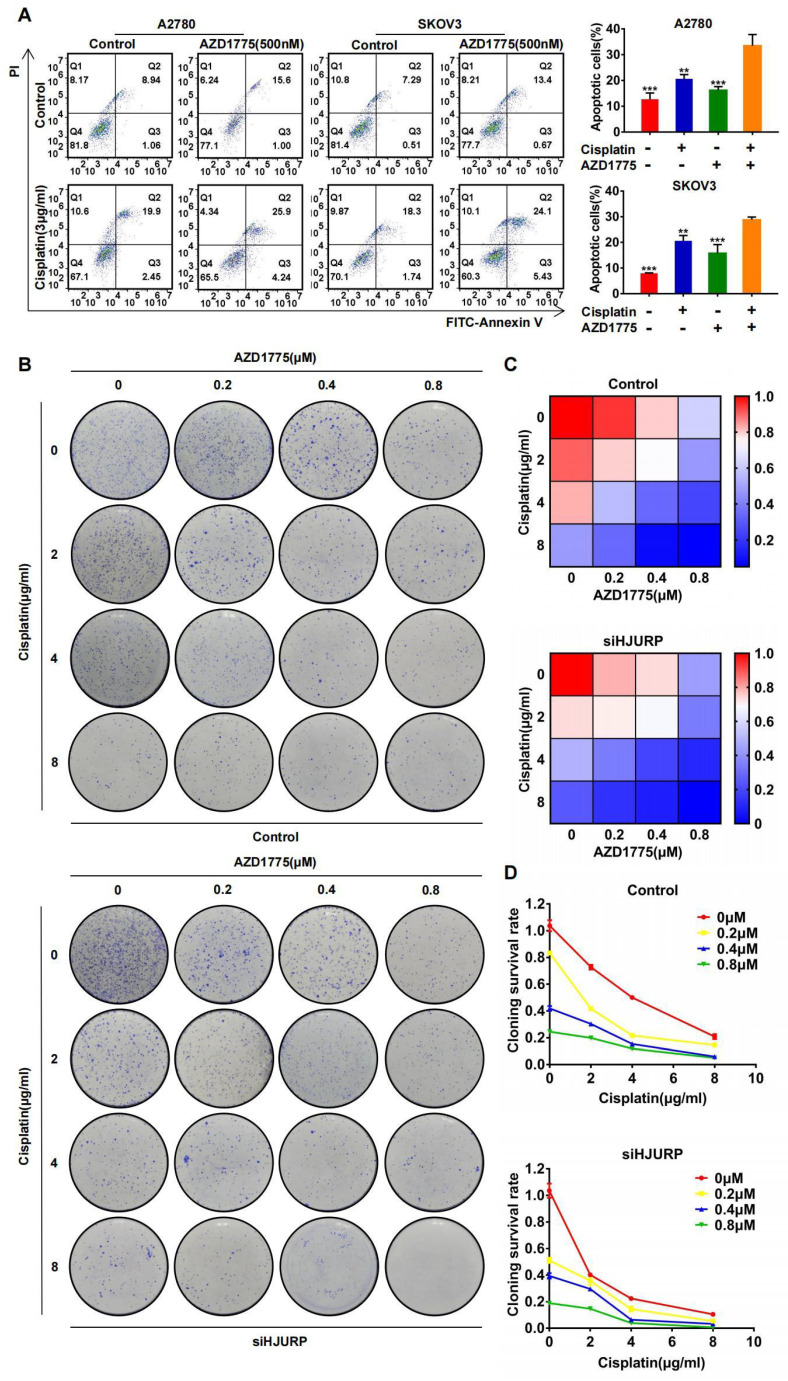
** Silencing HJURP could enhance the combination effect of cisplatin and AZD1775. (A)** Apoptosis assay was detected by flow cytometry to illustrate the effect of single agent or combinatory treatment of cisplatin and AZD1775. The cell population images were in the left, and the percentage of apoptotic cells was shown in histogram in the right.** (B)** Clonogenic assay was performed in control and siHJURP groups under the gradient combinatory treatment of cisplatin and AZD1775. Gradient concentration of cisplatin was 0, 2, 4, 8μg/ml, and that of AZD1775 was 0, 0.2, 0.4, 0.8μM. **(C)** Cloning survival rate was shown in heatmap. Red cells meant high survival rate and blue cells meant low survival rate. **(D)** Cloning survival rate was shown in line chart. Horizontal axis represented cisplatin concentration and each concentration of AZD1775 was plotted a single line. (Quantitative data are described as mean±SD, ^ns^ *P*>0.05, ^*^ *P*<0.05, ^**^ *P*<0.01 and ^***^ *P*<0.001).

The combined effect between AZD1775 and cisplatin has been widely reported^40^-^42^, and we have verified the synergism in A2780 and SKOV3 again. As shown in **Figure 9A**, apoptotic assay was performed to compare the effects of single or combined application of AZD1775 and cisplatin. Obviously, apoptotic percentage reached the highest level in the combined group both in A2780 and SKOV3. Furthermore, we detected whether the combined effect between AZD1775 and cisplatin was subjected to HJURP expression. As shown in **Figure 9B**, clonogenic formation assay was conducted in gradient concentration of the two drugs. Gradient concentration of AZD1775 and cisplatin formed a combined matrix. Relative survival rate of each well was calculated and plotted into heatmap. As shown in **Figure 9C**, the survival rate matrix in the heatmap crawled into top left corner in siHJURP group. Cloning survival rate curve was also plotted at every single concentration of AZD1775 (**Figure 9D**). The cloning survival rate was lower in siHJURP group at the same combined drug concentration. Collectively, the combined effect between cisplatin and AZD1775 was affected by HJURP, and silencing HJURP could promote the synergism of the two drugs.

## Discussion

Ovarian cancer is the most lethal gynecological malignancy globally, and high-grade serous carcinoma accounts for 70-80% ovarian cancer-associated deaths^2^,^3^. Recurrence and chemoresistance are still primary challenges required to overcome. In the present study, HJURP was found to be up-regulated in ovarian cancer tissues and considered to be a hub gene among DEGs in NGS data of ovarian cancer versus fallopian tube. Besides, high expression of HJURP was a risk factor for PFS, and high HJURP level of tumor specimens correlated with poor prognosis in ovarian cancer patients. What we demonstrated above was in agreement with previous studies of HJURP to exert oncogenic effects in various tumors such as lung cancer^7^,^14^, breast cancer^15^,^20^ and hepatocellular carcinoma^16^,^17^, et al. Based on the expression and prognostic value of HJURP in ovarian cancer, we speculated that HJURP probably participated in malignant progression of ovarian cancer. Then, we found that HJURP knockdown could inhibit ovarian cancer proliferation both in vitro and in vivo. Silencing HJURP could induce early G0/G1 cell cycle arrest with CCND1 and CDK6 down-regulated, and HJURP knockdown could decline metastasis abilities probably via affecting EMT process, in accordance with what Chen T, et al. demonstrated in hepatocellular carcinoma^17^,^49^. Additionally, HJURP was reported to participate in DSB repair^7^, which was a novel perspective for exploration of small molecular drugs. These results demonstrated that HJURP could serve as an oncogene and silencing HJURP could provide an opportunity for target therapy.

To further clarify the biological process and signaling pathways HJURP participated in, RNA sequencing of siNC versus siHJURP was performed for exploration of downstream networks related to HJURP. All DEGs of NGS data were used for GO and KEGG analysis. GO analysis revealed that HJURP was associated with cell cycle checkpoint, DNA replication, DNA integrity checkpoint, negative regulation of cell cycle process and signal transduction in response to DNA damage. KEGG analysis indicated that the main pathways regulated by HJURP were cell cycle, pathways in cancer, cellular senescence, small cell lung cancer and DNA replication. Our results illustrated the functional clusters of HJURP and provided more evidence for research of HJURP regulation mechanism. It was confirmed that HJURP mediated maintenance and deposition of CENP-A nucleosomes during G1 phase^8^,^9^ and involved in chromosomal stability^7^,^12^. Andronov L, et al. reported CENP-A nucleosomes could form rosette-like structures around HJURP during G1 phase^50^, in favor of basic function of HJURP as a chaperone of CENP-A in G1 phase. Moreover, HJURP was required for centromeric nucleosome inheritance and CENP-A retention during S phase^51^. Briefly, HJURP was a CENP-A chaperone, essential to centromere integrity and faithful mitosis. However, GO analysis presented above showed that DEGs with siHJURP could be enriched in cell cycle checkpoint and signal transduction in response to DNA damage. Cell cycle checkpoints could allow time for repair of damaged DNA^29^, beneficial to tumor cell survival. Kato T, et al. also reported that HJURP took part in DSB repair and correlated with immortality of cancer cells^7^. All findings reminded that HJURP probably played a role in malignant cell survival especially when it was abnormally over-expressed.

Considering the basic function of HJURP in cell cycle regulation, we reanalyzed NGS data with restricted threshold and selected GO enrichment process associated with cell cycle or DNA replication for further exploration. Hub genes were screened, in line with the function of HJURP in early G0/G1 regulation as for cell cycle. Moreover, we found that WEE1 was down-regulated when silencing HJURP. WEE1 was a G2/M cell cycle checkpoint^25^, and served as an independent prognostic marker in post-chemotherapy ovarian carcinoma^52^. Besides, combination of DNA-damaging agents and WEE1 inhibitor had possibilities to induce cell death synergistically and arouse mitotic catastrophe^31^,^32^. Rajeshkumar NV, et al. reported that AZD1775 synergized with gemcitabine in tumor regression of pancreatic cancer^53^. Combination of AZD1775 and cisplatin could exert synergistic effects on tumor inhibition of gastric cancer and medulloblastoma^40^,^41^. Moreover, inhibition of ATR could improve WEE1 sensitivity^54^, and combination of WEE1 and ATR repression produced tumor-selective synthetic lethality^55^. ATM and ATR kinases were central regulators of DNA damage response signaling pathway and facilitated genomic stability^56^,^57^. DNA damage response was a possible reason for chemoresistance^22^. Interestingly, HJURP was involved in ATM signaling and responded to DNA damage for maintenance of chromosomal stability^7^. Besides, we found that γH2A.X was up-regulated after silencing HJURP, implying that silencing HJURP probably brought about obliteration of DNA damage repair. Serafim RB, et al. had reported that HJURP knockdown could enhance sensitivity of glioblastoma cells to radiation therapy^21^. Based on the evidence above, we speculated that silencing HJURP could be beneficial to cisplatin or AZD1775 treatment and combined therapy was potential to be a novel strategy for resistant ovarian cancer.

Subsequently, the effects of silencing HJURP on cisplatin or AZD1775 therapy were assessed. We found that silencing HJURP could enhance sensitivity of ovarian cancer cells to cisplatin and AZD1775. Combined treatment could lead to more apoptotic induction. However, silencing HJURP could not cause apoptosis alone. Cisplatin was a classical DNA-damaging agent and AZD1775 could promote G2/M transition despite of DNA damage carrying. One possible explanation was that silencing HJURP aroused chromosomal instability and collapse of DNA damage repair, which might bring about ovarian cancer cells susceptible to DNA damage. Further, we aimed to explore an intermediate mechanism between HJURP and WEE1. Considering the response of HJURP expression upon DNA damage, we selected a GO process termed as positive regulation of response to DNA damage stimulus and found the correlation between HJURP, MYC and WEE1. Mechanistic experiments revealed that HJURP could regulate WEE1 through the transcription factor MYC, and rescued experiment indicated that HJURP mediated cisplatin chemoresistance partially because of MYC/WEE1 axis. Moreover, HJURP was necessary for DNA repair of cisplatin-induced DNA damage, which might decrease sensitivity of ovarian cancer cells towards DNA damage agents. Interestingly, the combined effect between cisplatin and AZD1775 was also affected by HJURP expression.

In conclusion, our results indicated that HJURP was over-expressed and promoted malignant progression in ovarian cancer. Cell cycle checkpoint, DNA replication and DNA integrity checkpoint, et al. were considered main functional processes based on enrichment analysis, and WEE1 was determined to be a downstream process associated with HJURP. HJURP regulated WEE1 through transcription factor MYC and modulated cisplatin chemoresistance via MYC/WEE1 axis. Furthermore, silencing HJURP could increase sensitivity to AZD1775 and affect DNA repair of cisplatin-induced DNA damage. Besides, high expression of HJURP was a risk factor for PFS, and high HJURP was correlated with poor prognosis. Altogether, HJURP has the potential to be a novel target in combined therapy and has the significant prognostic value of ovarian cancer.

## Supplementary Material

Supplementary figures and table headings.

Supplementary tables.

## Figures and Tables

**Table 1:** Clinicopathological analysis of HJURP low and high expression.(Before matching)

Parameters	Total	HJURP expression		P-value
		low	high	
Age (years)				0.590
<60	106	46	60	
≥60	50	24	26	
Tumor maximal diameter (cm)				0.731
<4	24	10	14	
≥4	132	60	72	
CA125 (U/ml)				**0.004**
<200	29	20	9	
≥200	127	50	77	
Ascites (ml)				**0.000**
<3000	111	60	51	
≥3000	45	10	35	
FIGO stage				**0.009**
I+II	38	24	14	
III+IV	118	46	72	
Omentum or peritoneum metastasis				**0.009**
Negative	42	26	16	
Positive	114	44	70	
Lymph node metastasis				0.278
Negative	46	25	21	
Positive	51	22	29	
Unknown	59	23	36	
Surgical status				0.491
Optimal	107	50	57	
Sub-optimal	49	20	29	

CA125, carbohydrate antigen 125; FIGO, Federation International of Gynecology and Obstetrics. P-value in bold font means statistically significant.

**Table 2:** Clinicopathological analysis of HJURP low and high expression.(After 1:1 matching)

Parameters	Total	HJURP expression		P-value
		low	high	
Age (years)				0.243
<60	86	40	46	
≥60	38	22	16	
Tumor maximal diameter (cm)				0.803
<4	19	9	10	
≥4	105	53	52	
CA125(U/ml)				0.473
<200	21	12	9	
≥200	103	50	53	
Ascites (ml)				0.638
<3000	102	52	50	
≥3000	22	10	12	
FIGO stage				0.675
I+II	30	16	14	
III+IV	94	46	48	
Omentum or peritoneum metastasis				0.687
Negative	34	18	16	
Positive	90	44	46	
Lymph node metastasis				0.911
Negative	38	19	19	
Positive	46	22	24	
Unknown	40	21	19	
Surgical status				0.318
Optimal	89	42	47	
Sub-optimal	35	20	15	

CA125, carbohydrate antigen 125; FIGO, Federation International of Gynecology and Obstetrics.

**Table 3:** Univariate and multivariate Cox regression analysis of OS.

Clinicopathological parameters	Variable	Univariate Cox regression		Multivariate Cox regression	
		Hazard Ratio (95%CI)	P-value	Hazard Ratio (95%CI)	P-value
Age (years)	<60	Reference		-	
	≥60	1.175(0.753-1.834)	0.478		
Tumor maximal diameter(cm)	<4	Reference		-	
	≥4	0.791(0.455-1.377)	0.407		
CA125 (U/ml)	<200	Reference		-	
	≥200	1.202(0.691-2.092)	0.515		
Ascites (ml)	<3000	Reference			
	≥3000	1.501(0.903-2.495)	0.117	1.355(0.813-2.259)	0.244
FIGO stage	I+II	Reference			
	III+IV	2.374(1.380-4.085)	**0.002**	1.110(0.369-3.341)	0.853
Omentum or peritoneum metastasis	Negative	Reference			
	Positive	2.480(1.477-4.164)	**0.001**	2.480(1.477-4.164)	**0.001**
Lymph node metastasis	Negative	Reference			
	Positive	1.422(0.847-2.387)	0.183	1.133(0.654-1.963)	0.656
	Unknown	1.907(1.129-3.220)	**0.016**	1.266(0.710-2.254)	0.424
Surgical status	Optimal	Reference		-	
	Sub-optimal	1.203(0.771-1.877)	0.415		
HJURP expression	Low	Reference			
	High	1.528(1.011-2.308)	**0.044**	1.400(0.925-2.119)	0.111

CA125, carbohydrate antigen 125; FIGO, Federation International of Gynecology and Obstetrics; 95%CI, 95% confidence interval. P-value in bold font means statistically significant.

**Table 4:** Univariate and multivariate Cox regression analysis of PFS.

Clinicopathological parameters	Variable	Univariate Cox regression		Multivariate Cox regression	
		Hazard Ratio (95%CI)	P-value	Hazard Ratio (95%CI)	P-value
Age (years)	<60	Reference			
	≥60	1.314(0.877-1.967)	0.186	1.248(0.824-1.891)	0.295
Tumor maximal diameter (cm)	<4	Reference		-	
	≥4	0.856(0.510-1.438)	0.558		
CA125 (U/ml)	<200	Reference		-	
	≥200	1.357(0.808-2.280)	0.249		
Ascites (ml)	<3000	Reference		-	
	≥3000	1.315(0.809-2.138)	0.270		
FIGO stage	I+II	Reference			
	III+IV	2.095(1.306-3.361)	**0.002**	0.462(0.142-1.502)	0.199
Omentum or peritoneum metastasis	Negative	Reference			
	Positive	2.668(1.670-4.262)	**0.000**	2.589(1.620-4.139)	**0.000**
Lymph node metastasis	Negative	Reference			
	Positive	1.373(0.853-2.210)	0.192	1.207(0.719-2.027)	0.477
	Unknown	2.285(1.400-3.728)	**0.001**	1.607(0.939-2.750)	0.084
Surgical status	Optimal	Reference			
	Sub-optimal	1.362(0.907-2.045)	0.137	0.789(0.487-1.277)	0.334
HJURP expression	Low	Reference			
	High	1.545(1.059-2.256)	**0.024**	1.453(0.995-2.123)	0.053

CA125, carbohydrate antigen 125; FIGO, Federation International of Gynecology and Obstetrics; 95%CI, 95% confidence interval. P-value in bold font means statistically significant.

## Abbreviations

HJURP: Holliday Junction-Recognizing Protein
CENP-A: chromosomal specific histone H3 variant
NGS: next-generation sequencing
PARPi: poly (adenosine diphosphate-ribose) polymerase inhibitor
DSB: double-strand breaks
CDK: cyclin-dependent kinase
PFS: progression-free survival
TMA: tissue microarray
IHC: immunohistochemistry
qRT-PCR: real-time quantitative polymerase chain reaction
WB: western blot
siRNA: small interfering RNA
NC: negative control
MTT: 3-[4,5-dimethylthiazol-2-yl]-2,5-diphenyl tetrazolium bromide
PI: propidium iodide
SPF: specific‑pathogen‑free
TCGA: The Cancer Genome Atlas
GTEx: Genotype-Tissue Expression
PPI: Protein-protein interaction
GO: Gene Ontology
KEGG: Kyoto Encyclopedia of Genes and Genomes
CC: cellular component
MF: molecular function
BP: biological process
EdU: 5-ethynyl-2'-deoxyuridine
IC50: half maximal inhibitory concentration
DEGs: differentially expressed genes
CA125: carbohydrate antigen 125
OS: overall survival
CI: confidence interval
EMT: epithelial-mesenchymal transition
P-adj: adjusted P value
