# Supplementary material for: HJURP Promotes Malignant Progression and Mediates Sensitivity to Cisplatin and WEE1-inhibitor in Serous Ovarian Cancer
Source: Int J Biol Sci. 2022 Jan 1;18(3):1188–210. doi: 10.7150/ijbs.65589 (PMC8771849; doi:10.7150/ijbs.65589)
Supplement: Supplementary file 1 — Supplementary figures and table headings. [file ijbsv18p1188s1.pdf]

Supplementary Figure S1. **(A)**Top 30 DEGs in **Figure 1B** was integrated with STRING database to establish a PPI network. **(B)**The PPI network integrated in **Supplementary Figure S1A** was calculated with Cytoscape software for the following analysis.

Supplementary Figure S2. **(A)**The relative mRNA of HJURP was detected by qRT-PCR to verify the efficiency of different siHJURP oligos. **(B)**Protein level of HJURP was detected by WB after silencing HJURP. **(C)**The relative mRNA of HJURP was detected by qRT-PCR to verify the efficiency of stable transfected HJURP knockdown cell lines. **(D)**The relative mRNA of HJURP was detected by qRT-PCR to verify the efficiency of stable transfected HJURP overexpression in SKOV3. **(E)**Protein level of HJURP in stable transfected cell lines was detected by WB. **(F)**Xenograft assay was performed in nude mice. The tumor images of control and HJURP overexpression group were captured. **(G)**Tumor weight in **Supplementary Figure S2F** was shown in scatter plot. (Quantitative data are described as mean±SD, ^{ns} P>0.05, * P<0.05, ** P<0.01 and *** P<0.001)

Supplementary Figure S3. **(A)**KEGG enrichment was analyzed based on all down-regulated DEGs in **Figure 3A** after silencing HJURP. **(B)**The union set of **Figure 3D** was integrated with STRING database to establish a PPI network. **(C)**The PPI network integrated in **Supplementary Figure S3B** was calculated with Cytoscape software for the following analysis.

Supplementary Figure S4. **(A)**Cell viability of A2780 with cisplatin treatment(0, 1, 2, 4, 8, 16μg/ml) was detected by MTT assay. **(B)**IC50 values of cisplatin were calculated using nonlinear regression equation by GraphPad Prism 7.0 software. **(C)**EdU assay was performed in control and siHJURP groups after corresponding cisplatin treatment(200×). Hoechst was used to label nucleus and determine total cell number. **(D)**EdU positive cell proportion was showed in histogram. **(E)**Clonogenic assay was performed in control and HJURP overexpression groups under the gradient treatment of cisplatin in SKOV3. **(F)**Cloning formation number of **Supplementary Figure S4E** was shown in histogram in the left. Cloning survival rate was shown in line chart in the right. (Quantitative data are described as mean±SD, ^{ns} P>0.05, * P<0.05, ** P<0.01 and *** P<0.001)

Supplementary Figure S5. **(A)**Clonogenic assay was performed in different conditions under the gradient treatment of cisplatin in SKOV3 and A2780. **(B)**Cloning formation number of **Supplementary Figure S5A** was shown in histogram, and cloning survival rate was shown in line chart. SKOV3 was laid above and A2780 was laid below. As for histogram, asterisks on blue-legend group meant the comparison between red-legend and blue-legend group, and asterisks on green-legend group meant the comparison between blue-legend and green-legend group. **(C)**Apoptosis assay was detected in A2780 and SKOV3 by flow cytometry to illustrate the rescued effect of siWEE1 after HJURP overexpression under cisplatin treatment. **(D)**The percentage of apoptotic cells was shown in histogram. A2780 was

laid in the left and SKOV3 was laid in the right. (Quantitative data are described as mean±SD, ^{ns} *P*>0.05, * *P*<0.05, ** *P*<0.01 and *** *P*<0.001)

Supplementary Figure S6. (A) Clonogenic assay was performed in control and HJURP overexpression groups under the gradient treatment of AZD1775 in SKOV3. **(B)**Cloning formation number of **Supplementary Figure S6A** was shown in histogram in the left. Cloning survival rate was shown in line chart in the right. (Quantitative data are described as mean±SD, ^{ns} *P*>0.05, * *P*<0.05, ** *P*<0.01 and *** *P*<0.001)

Supplementary Table S1. Primer sequences used in the present study.
Supplementary Table S2. Primary and secondary antibodies used in the present study.
Supplementary Table S3. Top 30 up-regulated genes of ovarian cancer generated by NGS.
Supplementary Table S4. DEGs(P-adj<0.05) between siNC and siHJURP generated by NGS.
Supplementary Table S5. GO analysis of all DEGs(P-adj<0.05) between siNC and siHJURP.
Supplementary Table S6. KEGG analysis of all down-regulated genes(P-adj<0.05) along with siHJURP.
Supplementary Table S7. GO analysis of restricted DEGs(log_2FoldChange>0.5 and P-adj<0.05) between siNC and siHJURP.
Supplementary Table S8. Cell-cycle-related DEGs(log_2FoldChange>0.5 and P-adj<0.05) between siNC and siHJURP from GO analysis.

A

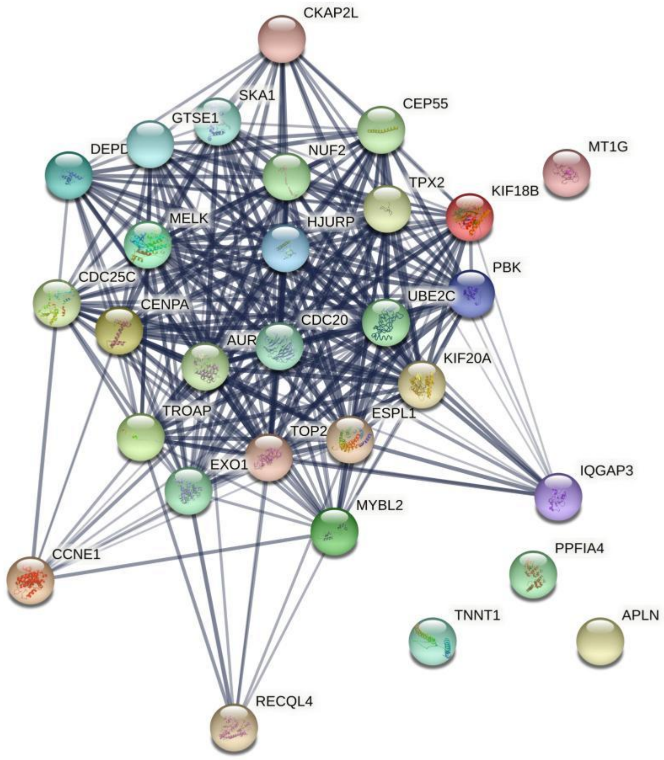
CKAP2L
SKA1
CEP55
GTSE1
DEPD
NUF2
MT1G
TPX2
KIF18B
MELK
HJURP
PBK
CDC25C
UBE2C
CENPA
CDC20
AUR
KIF20A
TROAP
ESPL1
TOP2
EXO1
IQGAP3
MYBL2
PPFIA4
CCNE1
TNNT1
APLN
RECQL4

B

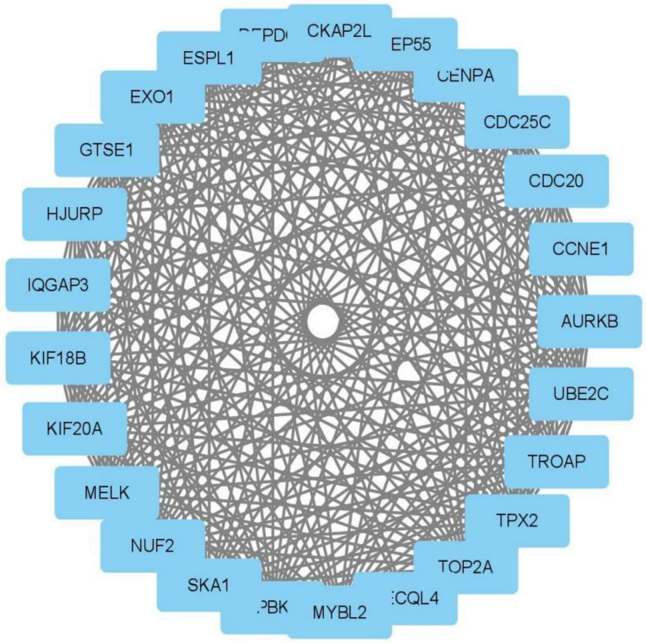
CKAP2L
EP55
ESPL1
CENPA
EXO1
CDC25C
GTSE1
CDC20
HJURP
CCNE1
IQGAP3
AURKB
KIF18B
UBE2C
KIF20A
TROAP
MELK
TPX2
NUF2
TOP2A
SKA1
ECQL4
PBK
MYBL2

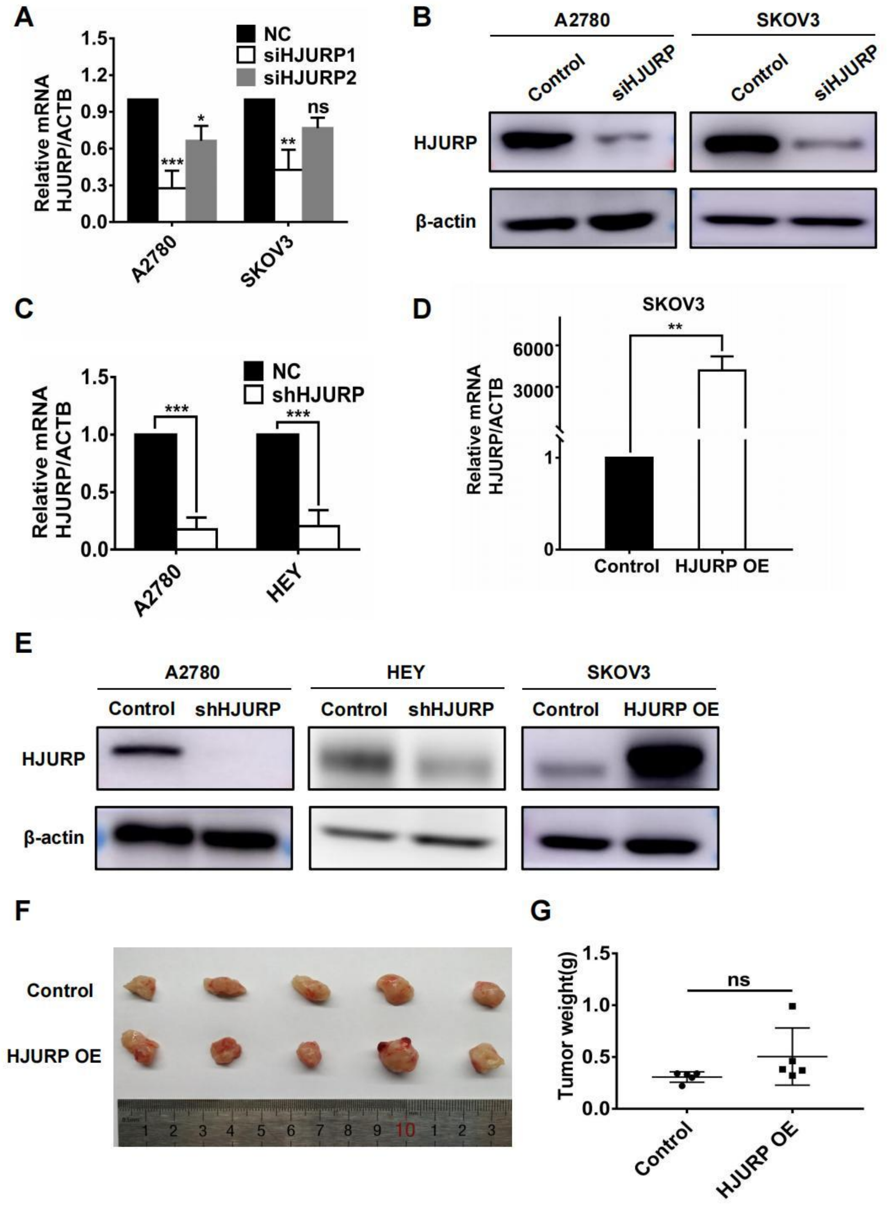

A
NC
siHJURP1
siHJURP2
Relative mRNA HJURP/ACTB
1.5
1.2
0.9
0.6
0.3
0.0
***
*
**
ns
A2780
SKOV3
B
A2780
SKOV3
Control
siHJURP
Control
siHJURP
HJURP
β-actin
C
NC
shHJURP
Relative mRNA HJURP/ACTB
1.5
1.0
0.5
0.0
***
***
A2780
HEY
D
SKOV3
**
6000
3000
1
0
Relative mRNA HJURP/ACTB
Control
HJURP OE
E
A2780
HEY
SKOV3
Control
shHJURP
Control
shHJURP
Control
HJURP OE
HJURP
β-actin
F
Control
HJURP OE
G
Tumor weight(g)
1.5
1.0
0.5
0.0
ns
Control
HJURP OE

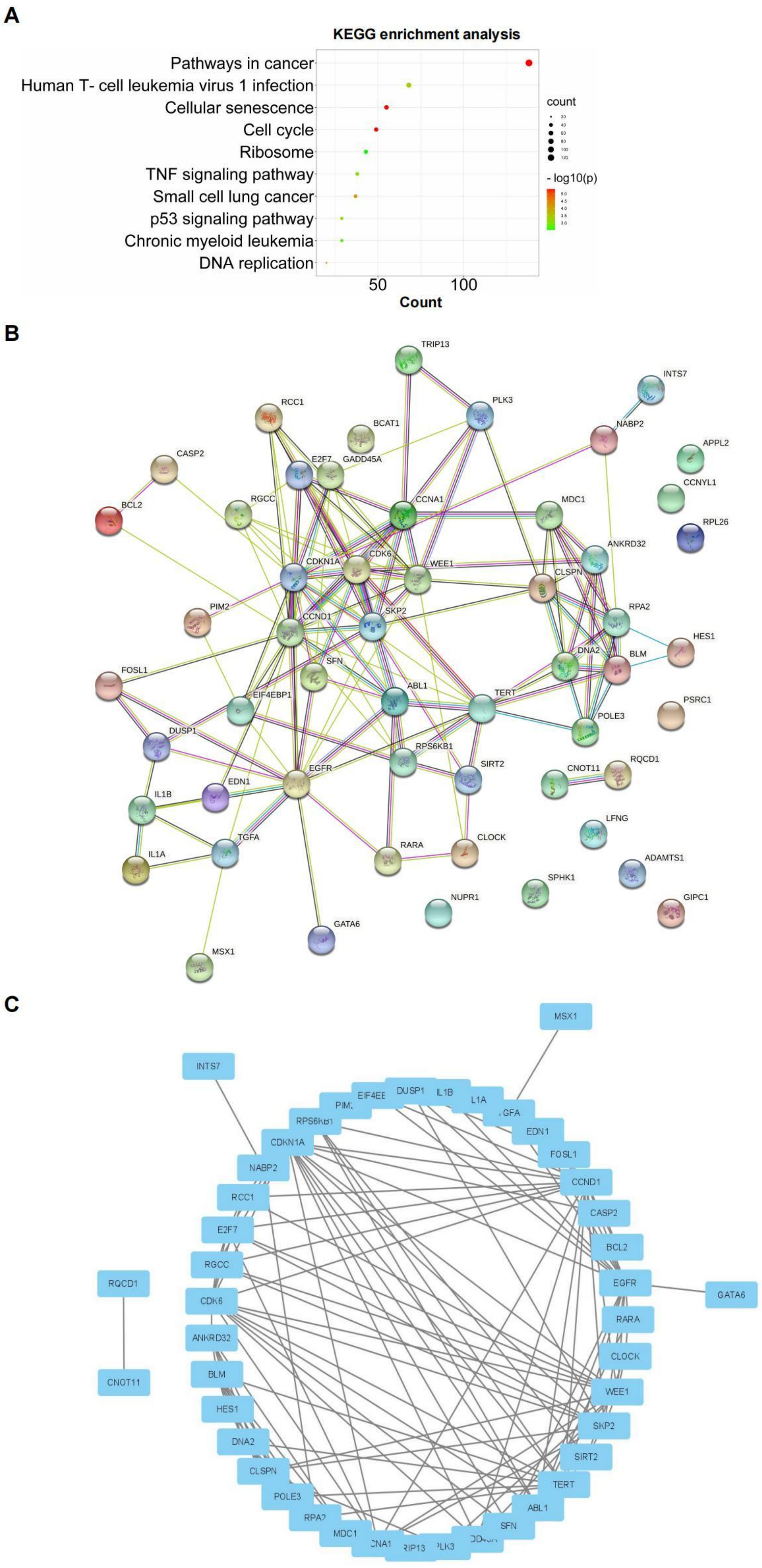
A
KEGG enrichment analysis
Pathways in cancer
Human T- cell leukemia virus 1 infection
Cellular senescence
Cell cycle
Ribosome
TNF signaling pathway
Small cell lung cancer
p53 signaling pathway
Chronic myeloid leukemia
DNA replication
50
100
Count
count
- log10(p)
B
TRIP13
INTS7
PLK3
RCC1
NABP2
BCAT1
APPL2
CASP2
E2F7
GADD45A
CCNYL1
BCL2
RGCC
CCNA1
MDC1
RPL26
ANKRD32
CDKN1A
CDK6
WEE1
CLSPN
PIM2
SKP2
RPA2
CCND1
HES1
DNA2
BLM
FOSL1
SFN
EIF4EBP1
ABL1
TERT
PSRC1
POLE3
DUSP1
RPS6KB1
SIRT2
CNOT11
RQCD1
EDN1
EGFR
IL1B
LFNG
TGFA
RARA
CLOCK
IL1A
ADAMTS1
SPHK1
NUPR1
GIPC1
GATA6
MSX1
C
MSX1
INTS7
DUSP1
RPS6KB1
EDN1
CDKN1A
FOSL1
NABP2
CCND1
RCC1
CASP2
E2F7
BCL2
RQCD1
RGCC
EGFR
GATA6
CDK6
RARA
ANKRD32
CLOCK
CNOT11
BLM
WEE1
HES1
SKP2
DNA2
SIRT2
CLSPN
TERT
POLE3
ABL1
SFN
MDC1

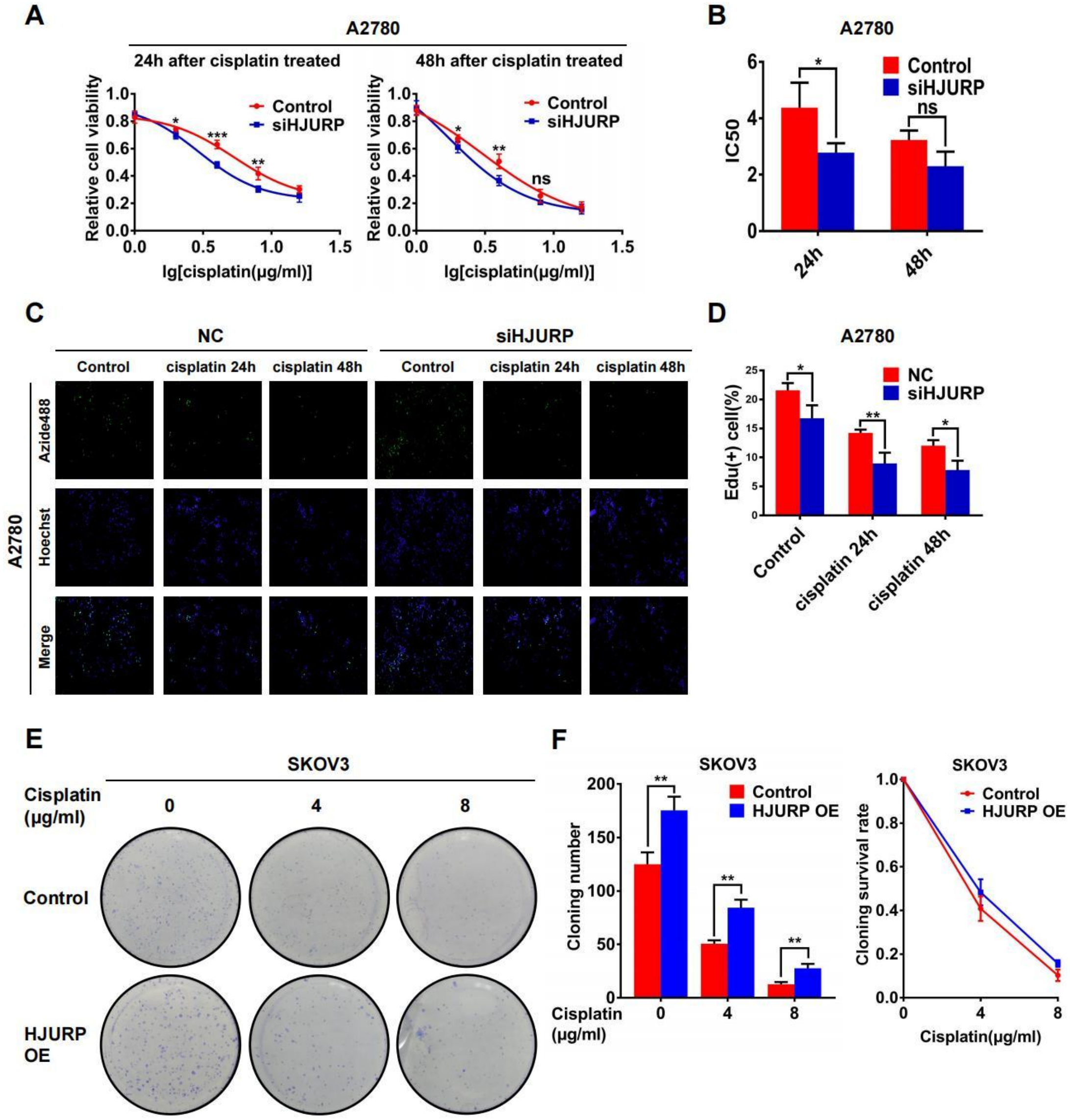

A
A2780
24h after cisplatin treated
48h after cisplatin treated
Relative cell viability
Control
siHJURP
lg[cisplatin(μg/ml)]
B
A2780
IC50
Control
siHJURP
ns
24h
48h
C
NC
siHJURP
Control
cisplatin 24h
cisplatin 48h
Azide488
Hoechst
Merge
A2780
D
A2780
Edu(+) cell(%)
NC
siHJURP
E
SKOV3
Cisplatin (μg/ml)
Control
HJURP OE
F
SKOV3
Cloning number
Control
HJURP OE
Cloning survival rate
Cisplatin(μg/ml)

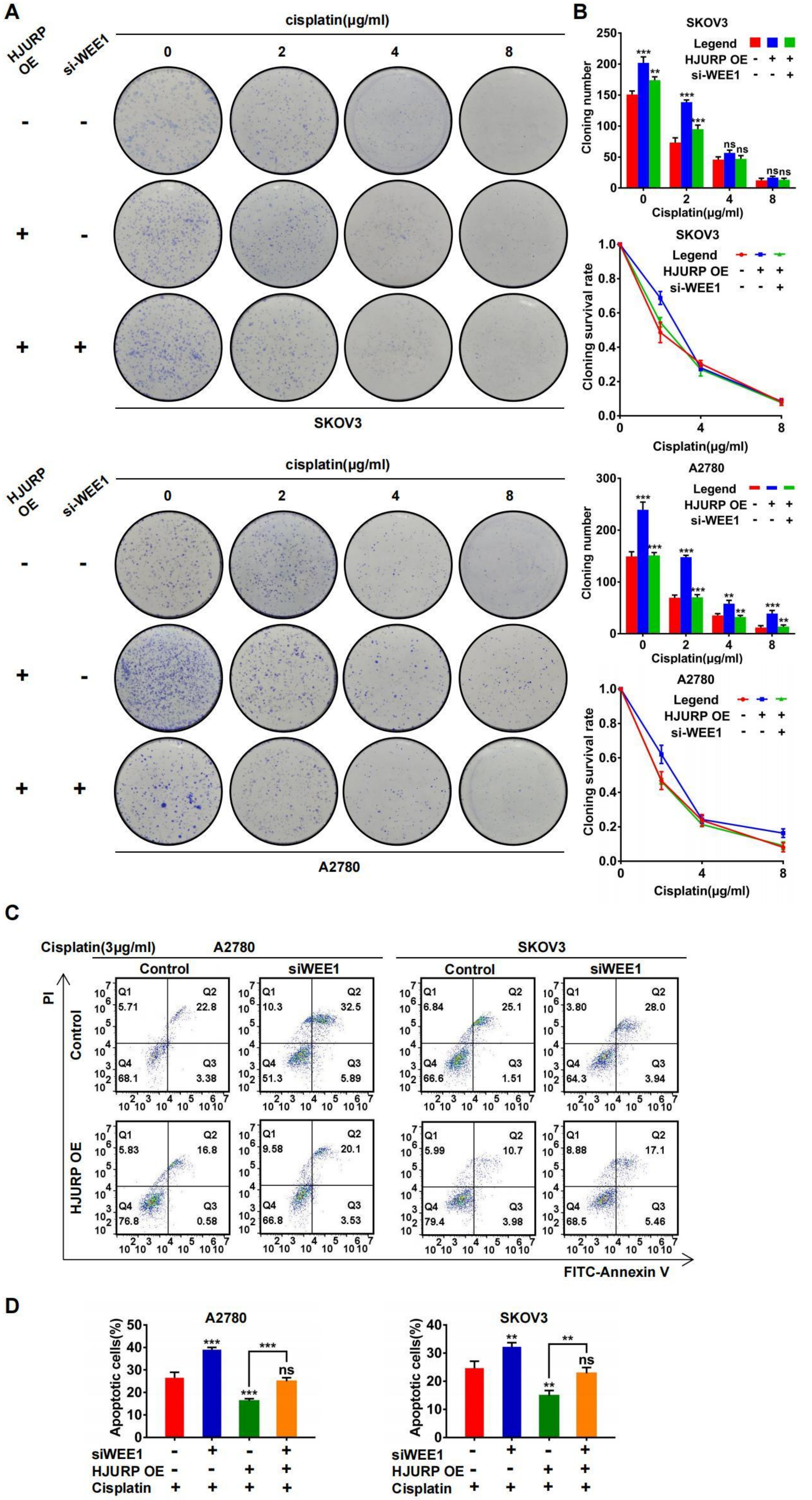

A
cisplatin(μg/ml)
HJURP OE
si-WEE1
0
2
4
8
SKOV3
A2780
B
SKOV3
Legend
HJURP OE
si-WEE1
Cloning number
Cisplatin(μg/ml)
Cloning survival rate
A2780
C
Cisplatin(3μg/ml)
A2780
SKOV3
Control
siWEE1
HJURP OE
PI
FITC-Annexin V
D
A2780
SKOV3
Apoptotic cells(%)
siWEE1
HJURP OE
Cisplatin

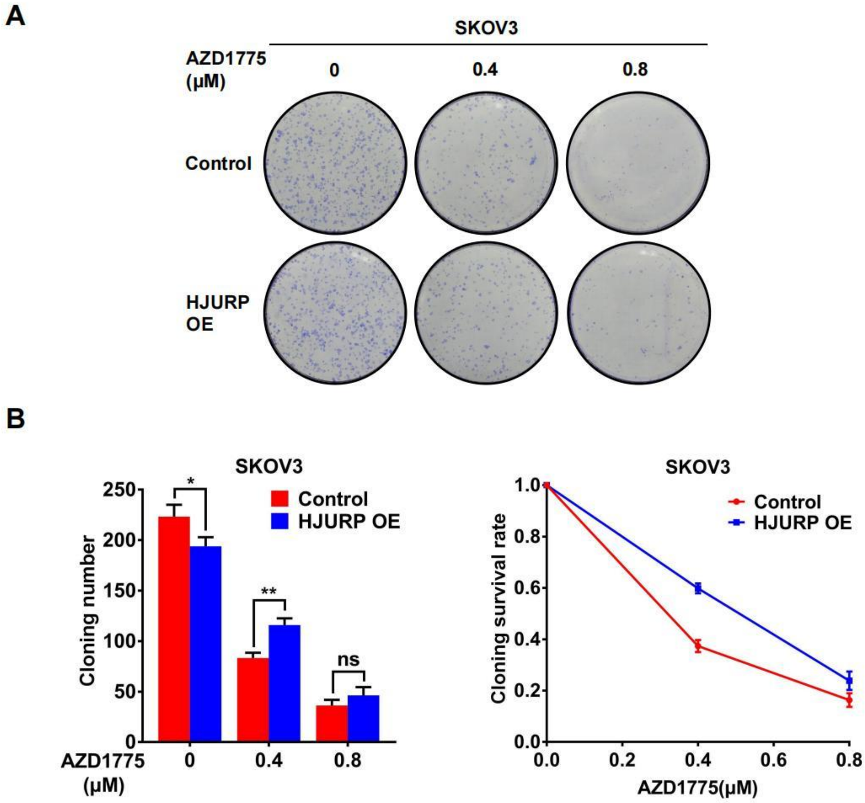
A
SKOV3
AZD1775 (μM)
0
0.4
0.8
Control
HJURP OE
B
SKOV3
Cloning number
Control
HJURP OE
*
**
ns
AZD1775 (μM)
SKOV3
Cloning survival rate
Control
HJURP OE
AZD1775(μM)
