# Supplementary material for: HJURP Promotes Malignant Progression and Mediates Sensitivity to Cisplatin and WEE1-inhibitor in Serous Ovarian Cancer
Source: Int J Biol Sci. 2022 Jan 1;18(3):1188–210. doi: 10.7150/ijbs.65589 (PMC8771849; doi:10.7150/ijbs.65589)
Supplement: Supplementary file 2 — Supplementary tables. [file ijbsv18p1188s2.zip › Supplementary Tables/Supplementary Table S1.Primer sequences used in the present study.docx]

**Supplementary Table S1.** Primer sequences used in the present study.

| Target genes | Primer sequences |
| --- | --- |
| ACTB forward | 5’-CATGTACGTTGCTATCCAGGC-3’ |
| ACTB reverse | 5’-CTCCTTAATGTCACGCACGAT-3’ |
| MYC forward | 5’-TCCCTCCACTCGGAAGGAC-3’ |
| MYC reverse | 5’-CTGGTGCATTTTCGGTTGTTG-3’ |
| WEE1 forward | 5’-AGGGAATTTGATGTGCGACAG-3’ |
| WEE1 reverse | 5’-CTTCAAGCTCATAATCACTGGCT-3’ |
| HJURP forward | 5’-GAAGGGATGTACGTGTGACTC-3’ |
| HJURP reverse | 5’-CCATTCTCTGGGAGATGAAGC-3’ |
