# Supplementary material for: HJURP Promotes Malignant Progression and Mediates Sensitivity to Cisplatin and WEE1-inhibitor in Serous Ovarian Cancer
Source: Int J Biol Sci. 2022 Jan 1;18(3):1188–210. doi: 10.7150/ijbs.65589 (PMC8771849; doi:10.7150/ijbs.65589)
Supplement: Supplementary file 2 — Supplementary tables. [file ijbsv18p1188s2.zip › Supplementary Tables/Supplementary Table S2.Primary and secondary antibodies used in the present study.docx]

**Supplementary Table S2.** Primary and secondary antibodies used in the present study.

| Antibody names | Experiment | Dilution | Company |
| --- | --- | --- | --- |
| Anti-HJURP antibody[EPR22619-41](ab233541) | WB | 1: 1000 | Abcam |
| WEE1(B-11) monoclonal antibody(sc-5285) | WB | 1: 1000 | Santa Cruz |
| N-Cadherin(D4R1H) XP® Rabbit mAb(13116) | WB | 1: 1000 | CST |
| Vimentin(D21H3) XP® Rabbit mAb(5741) | WB | 1: 1000 | CST |
| Slug(C19G7) Rabbit mAb(9585) | WB | 1: 1000 | CST |
| Cyclin B1(D5C10) XP® Rabbit mAb(12231) | WB | 1: 1000 | CST |
| Cyclin D1(92G2) Rabbit mAb(2978) | WB | 1: 1000 | CST |
| Cyclin E1(D7T3U) Rabbit mAb(20808) | WB | 1: 1000 | CST |
| CDK1 Polyclonal antibody(19532-1-AP) | WB | 1: 1000 | Proteintech |
| Phospho-cdc2 (Tyr15) (10A11) Rabbit mAb(4539) | WB | 1: 1000 | CST |
| CDK2 Polyclonal antibody(10122-1-AP) | WB | 1: 2000 | Proteintech |
| CDK4(D9G3E) Rabbit mAb(12790) | WB | 1: 1000 | CST |
| CDK6(D4S8S) Rabbit mAb(13331) | WB | 1: 1000 | CST |
| p21 Waf1/Cip1(12D1) Rabbit mAb(2947) | WB | 1: 1000 | CST |
| p27 Kip1(D69C12) XP® Rabbit mAb(3686) | WB | 1: 1000 | CST |
| BAX Polyclonal antibody(50599-2-Ig) | WB | 1: 5000 | Proteintech |
| BCL2 Polyclonal antibody(12789-1-AP) | WB | 1: 2000 | Proteintech |
| Phospho-Histone H2A.X Rabbit mAb(9718) | WB | 1: 1000 | CST |
| c-MYC Polyclonal antibody(10828-1-AP) | WB | 1: 2000 | Proteintech |
| Beta Actin Polyclonal Antibody(20536-1-AP) | WB | 1: 2000 | Proteintech |
| Anti-Mouse IgG (H+L) Antibody(5220-0341) | WB | 1: 6000 | KPL |
| Anti-Rabbit IgG (H+L) Antibody(5220-0336) | WB | 1: 4000 | KPL |
| Anti-HJURP antibody(ab100800) | IHC | 1: 200 | Abcam |
