# Supplementary material for: HJURP Promotes Malignant Progression and Mediates Sensitivity to Cisplatin and WEE1-inhibitor in Serous Ovarian Cancer
Source: Int J Biol Sci. 2022 Jan 1;18(3):1188–210. doi: 10.7150/ijbs.65589 (PMC8771849; doi:10.7150/ijbs.65589)
Supplement: Supplementary file 2 — Supplementary tables. [file ijbsv18p1188s2.zip › Supplementary Tables/Supplementary Table S3.Top 30 up-regulated genes of ovarian cancer generated by NGS.docx]

**Supplementary Table S3.** Top 30 up-regulated genes of ovarian cancer generated by NGS.

| Gene Name | P-adj. | Log_2_FoldChange |
| --- | --- | --- |
| UBE2C | 2.17E-10 | 6.5746 |
| IQGAP3 | 1.35E-09 | 6.205 |
| CDC20 | 1.38E-08 | 5.7958 |
| ESPL1 | 2.24E-08 | 5.7959 |
| MYBL2 | 3.45E-08 | 6.1173 |
| KIF18B | 4.57E-08 | 5.4895 |
| TNNT1 | 6.96E-08 | 5.4021 |
| MT1G | 8.45E-08 | 9.0554 |
| KIF20A | 8.54E-08 | 5.2865 |
| MELK | 1.13E-07 | 5.2612 |
| CDC25C | 1.44E-07 | 5.4892 |
| TPX2 | 1.58E-07 | 5.1542 |
| TROAP | 1.79E-07 | 5.4254 |
| APLN | 1.93E-07 | 5.7901 |
| CENPA | 3.75E-07 | 5.198 |
| HJURP | 4.16E-07 | 6.2952 |
| DEPDC1 | 5.31E-07 | 5.2254 |
| PPFIA4 | 6.49E-07 | 5.5749 |
| EXO1 | 7.75E-07 | 5.0645 |
| RECQL4 | 8.86E-07 | 4.9565 |
| GTSE1 | 9.50E-07 | 4.8896 |
| PBK | 1.41E-06 | 4.8652 |
| AURKB | 1.71E-06 | 4.8351 |
| NUF2 | 1.75E-06 | 4.7843 |
| SKA1 | 2.19E-06 | 4.9568 |
| CEP55 | 2.21E-06 | 5.3215 |
| CKAP2L | 2.35E-06 | 4.6968 |
| TOP2A | 2.41E-06 | 4.6131 |
| CCNE1 | 2.82E-06 | 4.5349 |
| TACC3 | 3.44E-06 | 4.9141 |
