# Supplementary material for: HJURP Promotes Malignant Progression and Mediates Sensitivity to Cisplatin and WEE1-inhibitor in Serous Ovarian Cancer
Source: Int J Biol Sci. 2022 Jan 1;18(3):1188–210. doi: 10.7150/ijbs.65589 (PMC8771849; doi:10.7150/ijbs.65589)
Supplement: Supplementary file 2 — Supplementary tables. [file ijbsv18p1188s2.zip › Supplementary Tables/Supplementary Table S4.DEGs between siNC and siHJURP generated by NGS.docx]

**Supplementary Table S4.** DEGs(P-adj<0.05) between siNC and siHJURP generated by NGS.

| Gene Name | P-adj. | Log2FoldChange |
| --- | --- | --- |
| VIM  TPM4  S100A16  PLAU  H2AFX  SZRD1  GANAB  EMP1  WWTR1  HJURP  EGFR  PICALM  CCND1  JPT2  PXN  TRNP1  ZNF664  GPAT3  RTN4  SLC20A1  YBX1  SH2B3  DEK  PAPOLA  TRIP13  SS18  CXCL8  G0S2  FOSL1  SKP2  RNF38  AAK1  G3BP2  PGK1  SEMA4B  LAPTM4B  TXNIP  NDRG1  GLE1  CIZ1  VEGFA  DUSP3  COLGALT1  CD164  RRAS2  COL4A1  KDELC2  WNT7A  CSF1  SLC2A1  NCOA6  TINAGL1  CASC19  STC1  AFAP1L1  EFNB1  TIMP1  HSDL2  NR1D1  TUBB6  ETS1  MFSD14B  CTSB  CYP1B1  PIK3CB  EPHA2  GADD45A  NOLC1  TNFRSF10B  SLAIN2  SF3B2  NTN4  RPN1  ERRFI1  SCRN1  CDCA4  PKM  F3  SLC25A39  MESD  ARHGAP23  LAMA5  GLUD1  HAS3  ERO1A  GALNT1  CASP7  GPRC5A  R3HDM1  OGFR  ERLIN2  SMG7  PHC2  SLC39A8  TNFRSF10D  CD44  CREG1  SPRY4  CYFIP1  PIP4K2A  ANP32B  NETO2  TTLL12  HNF1B  POLE3  PLAT  PERP  GIPC1  NUP43  SFT2D2  MMP1  EHD4  KPNA3  IGF2BP2  ATP13A2  SNAP29  ZW10  ARL6IP5  PIP5K1A  NEAT1  EHD1  ZMIZ1  HMGA1  UBASH3B  BZW1  TBC1D9B  EI24  PHLDA2  HABP4  ISOC1  CASP3  MAL2  LONP1  NRBF2  SLC9A3R2  LHX1  SERPINB9  ENSA  CDKN1A  CDK16  FAM129B  RAB5B  METRNL  GJC1  PLAUR  FAM32A  MRPS2  LPCAT1  GOLGA2  DLC1  SEC31A  YBX3  DYNLL2  KDM2A  CTTN  GLRX  NCEH1  SYNJ2  ELF4  XXYLT1  DKK1  NFYC  NUP50  PRMT6  STX12  KDM5B  SLC25A24  VCL  CLINT1  DCP1A  ME1  B4GALT1  ZNF512B  FRMD6  PNP  ELAC2  PTMA  RNF126  CXCL1  SIGLEC15  DCBLD2  SGTA  SOX7  CDS1  ANXA2  PON2  TAB2  REEP3  ZFP36L2  CNOT11  CAV2  SPCS3  CDK14  C22orf46  ZMYM3  INSIG1  APPL2  FUS  SIPA1L3  CAPRIN2  MRGBP  RPRD1B  CHST14  ANGPTL4  NEMP1  ZNF639  GORASP2  XPO6  AMMECR1  STK17B  IGSF3  ZNF629  RABL6  HIPK1  PHLDA1  MAP3K2  TOB1  KRT18  RAD23A  TRIM8  SFRP1  CNOT9  ITGA2  FNBP1  UBE3A  ABL1  TBC1D10B  LRRC8C  ANXA5  CMTM3  LEPR  HDGFL2  EIF3F  KHNYN  STX2  IER3  DUSP4  MTMR6  GADD45B  PRDX4  IER5  NFIB  NAA30  FAM214B  MYO18A  TSPAN14  SOX17  CLIC4  ZNF271P  NUP153  FOXQ1  C6orf62  TLK2  RAC1  WDFY1  DHX37  ID1  YAP1  RBPMS  DENND5A  CCDC86  C12orf75  NRAS  CFDP1  RANBP9  UNC13B  SOX13  LAMB1  PLXNA2  ATG16L1  MICAL2  ELOA  ZDHHC16  LAMB3  AFAP1  AFG3L2  NIFK  PPDPF  CCDC9B  LIMK1  KHSRP  GNPDA1  DLG1  SLC25A22  KIRREL1  NFKBIZ  RAB31  HR  FLOT1  FOXP4  LOXL2  SH2B1  FGF18  DHRS2  TMEM173  TNRC6A  COASY  TUBB4B  CFL2  FOXN2  RAB6A  NDUFS2  SLCO4A1  IL4R  NHLRC3  POLR2D  EZH1  HES1  KRT8  RPL5  SLC6A6  TENM2  FSCN1  RAPGEF3  GRK6  LYPLA1  CCAR1  PCNX3  MYOF  SC5D  LAMC2  DUSP1  PODXL2  RAB3B  ANTXR2  STX3  GLYR1  RTN3  LSM12  SPARC  FAM222B  CCDC80  SULF2  RNF182  PSRC1  UNKL  FAM20C  WBP11  SERINC2  ZNF468  UHRF1  RGS10  MBOAT2  ZBED2  HEG1  TNS1  ADCY9  LAMTOR3  MSMO1  PCDH1  HOXB2  HBEGF  ARHGAP29  FZD1  ZNF678  ITPRIP  CHST11  WEE1  ELK3  TRIM25  LINC02582  SLC38A2  CLOCK  NKIRAS2  NORAD  LPP  CENPF  MKNK1  MIEN1  OSGIN1  RFX7  ASAP1  MCFD2  ZNF721  ZFYVE16  TENM3  TNFAIP2  DDIAS  ADAMTS1  MSR1  DPYSL3  UBALD2  LINC02535  RHOC  TNFRSF1A  CDC42EP4  IBTK  PXDN  NFE2L1  MYO1B  TMEM167A  PSMC3  SMAD3  MCM4  KLHDC10  ZNF765  FOPNL  GFPT2  SPP1  SENP1  RNF170  SIKE1  EDN1  CPNE3  ZNF100  RARA  HEXIM1  ZNF395  BNIP2  CDCP1  COL4A2  GAS2L3  BCAR3  CALM2  PRKAA2  ITGA5  RAB11FIP2  PLEKHB2  ARHGEF2  NRP2  TOMM22  WDR19  ZZEF1  IL6  ATRX  AZIN1  AGO2  MRFAP1  CDC42EP3  PRPF4B  LRRC8A  IQSEC1  S100A10  ELF2  FZD8  EPN1  NRM  PTGS1  MAMLD1  TNS2  DCTN4  EVI5L  BMI1  IDS  CCDC68  SFXN3  MAP3K7  ZSCAN29  EIF4A2  EXOC2  PPFIA1  RUNX1  CHST10  MTERF3  ZNF354A  HSP90AA1  ANPEP  NFIX  EIF4EBP1  GIT1  MT2A  HELLS  PPIP5K2  KIF1C  S100A11  AMN1  PLEKHG2  BNC1  ESM1  ZNF558  PMAIP1  MSRB3  MED13L  ZNF714  CDS2  KIAA1549L  MAFF  LAMA3  SH3BP2  PLAGL2  GLCE  CABIN1  RCC1  DNAJB6  MALAT1  DEPDC1B  CARNMT1  TADA3  CYB5RL  HDGFL3  ATN1  IGFBP3  SEC24B  KITLG  ZNF616  BCAR1  GFPT1  YIPF5  C12orf43  PLA2G12A  COPG2  GXYLT1  FAM217B  AC005392.2  CFAP36  BMS1  RXRB  SEPT11  FAM20B  CCDC85B  WASF3  TMEM189  CUX1  PLEKHA6  TK1  ZBTB5  DUSP6  FLYWCH1  CASP2  AC015813.1  SIRPB1  G3BP1  CCNYL1  EIF5A2  H3F3B  SLC26A2  CUL7  DZIP3  FBXW11  ARL4C  HERC3  BLOC1S6  GPRC5B  LMLN  CAPRIN1  ARPC3  DENND6A  FAM83A  MAPKAPK3  C11orf24  DHTKD1  ETAA1  DAB2  SPRY2  PLVAP  RGS19  ITGA3  NR3C2  NPRL3  NUDT16  ZKSCAN1  MISP  LSS  EFNA5  SIRT2  PAK4  FXR1  ELP1  MDC1  QSOX1  SKA2  ARL14EPL  BECN1  C9orf78  CDK6  RBM7  SELENOI  TMPO  ELL2  ZNF585B  TGIF1  PYGB  KIF11  PTPRJ  SART1  GINS1  SPHK1  IGFBP1  CD276  SPIN4  RASSF8  HOOK3  SET  ZNF83  STK32C  ADGRA3  ZNF41  ZNF33B  IVNS1ABP  ICMT  ITPR2  ADGRL1  ULBP3  R3HCC1  SEC14L1  NABP2  PLEK2  ETV4  NOV  IL1A  PNO1  HNRNPUL1  SLC43A3  C19orf53  APH1B  CLIP1  PPM1A  FUBP1  SNX33  ZNF618  COL8A1  TUBA4A  SDC2  GALE  PAFAH1B1  IRF2BP2  PFKL  GULP1  BSG  CCT5  UFL1  UBE2L3  KLF6  SECISBP2L  AC004846.1  PLRG1  UBE2Z  OSBPL5  ZNF605  ZNF91  LFNG  WBP4  C19orf54  KIF18B  NEIL3  INTS3  MCL1  PURB  VAMP3  TMEM192  USB1  KIAA1217  P3H2  MAP3K3  ARF6  HIVEP3  ARHGAP21  RCC1L  HDAC6  VCAN  ECE1  ZNF492  HIPK2  PAN2  ARL5A  MMP2  TSPAN5  MTHFR  USP12  DHX15  GTF2A2  CNEP1R1  ATP2A2  CEBPB  PEX19  FEN1  PROSER2  PDE7A  PLEC  AL161431.1  FOXF2  CCDC137  ZNF45  ITGB5  TRIM66  CEP104  PRMT2  TBC1D8  CDKN2AIP  PLXNB2  FAM173B  OAF  TSHZ1  ADGRG1  LIF  BRD4  NSUN4  PEX26  TMEM158  GJB3  PHF3  NRAV  ARFGAP1  VPS53  ZCCHC3  PLPP3  LPXN  LRRC17  PRRC1  CLCN3  RAPH1  ZNF254  NLRP1  ANKRD13B  SNX17  NT5E  CYP4F11  AMD1  ZDHHC20  PDE8A  UNC45A  ALYREF  ZNF622  THBS1  RAB8B  KIAA1551  PCBD1  ELMOD2  SHTN1  AC005077.4  ZNF397  CLSPN  KREMEN1  FDFT1  AES  ELFN2  KDSR  PPP6C  KLF10  DPH3  CUL9  KCTD5  KCTD20  TRAM2  FAM198B  NPTN  LYAR  NUP160  PTBP3  MPRIP  SMAD5  GPC6  TWIST2  SRPX  NOSIP  ZNF415  PPP1CB  ANXA7  ISCA1  BCL9L  SACM1L  TMOD1  ITGB3  P4HB  HGH1  PYGO2  ERBB2  HMGCS1  INCENP  PPP3R1  CDK9  FMN1  ZNF76  IFT81  ZNF845  ST3GAL1  SP1  ARSJ  FAM49A  ATAD2  ZNF703  KDELR2  ZNF275  CTIF  CNIH3  RPA2  SFN  DCAF6  DENND4A  AURKA  JUNB  LASP1  VTA1  PSKH1  GOLGA4  PFAS  TTLL4  RMND5A  PTPN14  GNG12  C2CD2  MYC  ZNF430  PROM2  SKI  EXOSC6  ANGPT2  TRPS1  NMNAT2  UVSSA  TMEM245  RPRD2  PTDSS1  MAPK6  NPIPB5  TRIB1  RAP2B  FYCO1  MAU2  DNAJC13  C1QTNF6  PPP3CB  FKBP1A  ERI1  PPTC7  MAP4  ZNF252P  ARF4  CALR  CLP1  CTGF  MYADM  BCKDK  SAMD5  EXOC4  UBE2R2  SEMA3F  C1orf131  ANK1  GSR  SLC11A2  SNHG16  GAS2L1  ATF6B  MAML2  CAPN2  ZNF33A  LARP4  IDH1  TRAF4  ZNF117  CLCN7  TGM2  ZNF532  SLC35G1  ALS2CL  RUNDC1  SIRPA  USP22  MBTPS1  RRM2  RPUSD1  GPR158  PRDM2  CMC2  RBBP4  MUM1  RAD18  C19orf12  POLR3H  PDLIM5  ARHGAP22  FAF2  EPS8  PKNOX1  C1orf116  CDT1  THSD4  CD9  AP3M1  TMBIM6  DENR  SH3BP4  BAIAP2-DT  AKT1S1  PPP2CB  CDC25B  AMTN  PGM2L1  SYT16  WIPF2  BMPR2  SQLE  PARD6G  NACC1  BISPR  CTBP2  DDX24  TNKS1BP1  SRPRA  NADSYN1  TCTA  ACTG1  JAG1  TRIM58  RPS6KB1  MCMBP  AFAP1L2  TGFA  CCDC113  TMEM131  MBOAT7  MSX1  PPP1R9B  DNAJC9  GSTZ1  STAG1  PEAR1  NBR1  NQO1  RRAD  ZNF160  NUP58  KRT15  RMDN1  PLIN3  EXOC5  XPO1  NCS1  MKNK2  SYPL1  CAPZA1  CHST2  ALKBH5  RPS6KA5  ADNP2  GOT2  SMIM3  C4orf46  ABHD3  CCNA1  MYOM3  TP53BP1  FAM49B  ATP1A1  C1orf43  TMEM164  ZDHHC5  SMOX  TNPO1  USP39  KAT14  NSMAF  RPTOR  CCNG1  CRELD1  BAHD1  CES1P2  ASCC2  RNF114  SLC35A4  SLC4A11  CEP85  PODXL  FAM83D  AHDC1  MRFAP1L1  CGNL1  DUS1L  USP5  IFT140  GLTP  ZNF134  TMEM69  HM13  TK2  GLUL  SLC36A1  PCNA  NOTCH1  ZNF43  ZKSCAN2  UCA1  LDHA  SLC35F2  ZNF426  SYDE1  IPO5  SNX9  CSRP1  DTWD2  DSTYK  MORC4  CERK  PARD3B  SNX30  PER1  JMY  DAG1  TOMM40  CPNE2  SASH1  PRKAR2A  CYB561D1  ATG12  HERPUD1  CBFB  CMTM7  ANXA6  SREK1IP1  CCNY  TAGLN  DNAJB11  KIAA1522  ZNF138  RABEP2  ZNF37A  TOP1  REV3L  PHLDB2  ZRANB1  NIPSNAP2  INTS7  SHISA2  S100A6  BTG1  ZSCAN30  BBS2  GPAA1  COQ2  RPS6KA2  POLR3E  LAP3  IL11  ZNF681  LRRC45  MAP3K9  ATP5F1C  PPP1R37  IL1R1  C20orf96  PITX1  TMEM8B  TNS3  E2F4  TTC14  KIF1B  SLC17A5  TERF1  YBX1P1  CREB1  CIART  CDC20  CKAP4  FAM219A  UAP1L1  RBMS1  COL5A2  RALGPS2  SLC16A3  ENC1  TFG  ZIC2  CKB  OTUB2  SEPHS1  RND3  SLC4A1AP  FAM210B  RAB2A  RBM3  DAPK3  LRRN4  HTATSF1  LAMB2  COG2  TXNRD1  EFHD2  BRPF1  C5orf15  FGFBP1  ZNF850  MRPL39  CAPG  ANXA2P2  KRT81  ARAF  AGPAT1  ACBD4  RMI1  TMEM33  CUTALP  LRCH1  GTF2I  ACOT9  LAMC1  CLUAP1  GBA  PCMTD2  TRPC6  PIGS  HMG20B  NLRP2  THBS4  AUP1  TARS2  SPIN1  SLC7A1  NAGLU  LINC00205  STK38  ACO2  FBLIM1  SMTN  CNIH1  BCAP29  AFF1  MOB1A  RAD54L2  GRAMD2B  PORCN  FLI1  DHCR24  NAA35  DAD1  CD55  AHCYL1  NAV3  COQ8B  TBC1D17  ZNF652  ORC2  TMEM9  CCDC198  KBTBD6  DGCR2  MTRR  NUDC  SOCS3  ECI1  PLEKHM1  ICK  TDG  ZBED4  GYS1  PMVK  AKAP12  SERPINE2  NXT1  MCM10  STK38L  SRP68  CASC3  MAN1B1  INPP5F  ERLIN1  CLN6  SCD  PCNX4  RGS3  AGL  TJP1  DUSP5  TENM1  DAZAP2  IGF2R  ACADSB  GTPBP4  DRAXIN  NXPE3  ZBTB44  COTL1  TMEM219  PLEKHA7  MET  CHCHD4  CSTB  CAB39  ZNF317  SEPT9  TEX19  METAP2  MCCC2  FBN1  PHB  ASPH  SPPL2A  FAM167A  EMC3-AS1  BCL2L13  SKIL  GABPB1  PSTPIP2  EGLN1  AP3D1  ADPRHL1  SEMA6A  MLLT6  PML  TSC22D1  KIDINS220  STK25  MRTO4  PVR  PPP2CA  THBS3  TRPC4AP  UPP1  PRNP  KLF5  DTL  S100A4  MYH9  SPSB1  SEC13  FANCA  PPIL2  PTPN11  EP300  CDH6  CCDC84  RPL26L1  DUSP7  TGOLN2  IER3IP1  SLC41A1  ZFAND5  OR7E19P  KLF13  CNTROB  MED15  IL15RA  CLTC  D2HGDH  SLC25A6  PAQR8  ZNF148  ZNF227  BX322639.1  DNA2  MRPS34  CSK  ABCC4  CEP68  MTM1  ITPA  TGFBR1  EXOSC2  COL3A1  OGFRL1  IL18  ZNF544  C1RL  EPHB2  TMEM129  UGDH  HCFC1R1  NOP53  TCTN2  VOPP1  FBXO25  GSKIP  BLVRB  ZNF429  ST6GAL1  ATP6V1E1  PBX2  PANX1  CD59  SOX12  SH3TC2  ZNF766  PAX8  DIXDC1  NCKAP1  RTL6  GNPNAT1  TOR1AIP1  KIAA0355  TMEM231  AGFG1  UAP1  ZNF720  RIPK4  PHACTR2  CYB5R3  PCNT  SMC1A  MED11  LYN  COPE  SRBD1  ZC3H3  CUL4B  USP40  FGF2  CDK17  ZNF180  PRPF19  ACTR1B  TFAM  PIR  ZNF493  PPP6R2  TUBA1B  POLI  NACC2  DYNC2H1  CFAP47  FECH  NOP56  KLF11  TWF2  NSD1  MYL12A  MAP2  ANKRD27  MYH14  ELP3  ARFGAP2  KPNA5  GLI2  KIT  HSPA5  UBE4B  FGFR1OP2  TRIM37  RAE1  ELMO2  CHSY1  CRAT  SNAPC2  FCHSD2  PIK3R1  GPR155  CNN3  SLC35F5  SPRED3  SERTAD4  MRAP2  ZER1  LINC00963  SFSWAP  P2RY1  ZBTB37  FAM84B  FLJ22447  LIN7C  GRID1  NUP214  SCAMP1  PKMYT1  TMEM109  CDK2  HEXDC  CD99L2  SLF1  VAC14  ESYT1  CD40  IL6R  WWP1  PFKFB2  LINC00941  TES  CYP2R1  GPBP1L1  ETS2  CCDC40  DPYSL5  ALPK1  PTPA  CREB3  PDRG1  TRAM2-AS1  ING1  NAP1L3  SUPT3H  CHAMP1  LINC00346  L1CAM  MAP2K3  PKIA  SLC7A5  AP1S2  RIN1  ADAM15  RNASEH1  GTF2H1  MED24  LPCAT2  S1PR3  ERGIC1  SQOR  TIAM1  MVD  NDRG4  UPF1  MAPK3  TMSB10  TMEM106C  SEC14L2  RNF214  LGALS1  NPEPPS  SLC30A6  ATP11C  E2F7  ZNF248  CRIM1  MAD2L1  SERTAD2  ZDHHC9  PUS1  TBL1XR1  VPS35L  IRS1  ZNF471  CHST15  TAB3  RHPN2  TNS4  BCAS4  C18orf54  PSPC1  SBF1  GEN1  PPP1R11  YOD1  NET1  PRKAG1  CCDC57  LSM12P1  ZDHHC24  AP000812.3  AGPAT3  MLKL  BICD2  LIG4  REXO4  GDI1  KARS  GATA6  AJUBA  TRERF1  CREBL2  AC005336.1  TRIM41  ARSB  ENDOV  ALDH1B1  INTS2  TSC22D2  PDCL3  GUCD1  FRS2  PAPLN  ADA  ID4  ANXA3  ACER3  PHTF1  ANKRD42  AC091729.3  MRPS5  TIMP2  ANKRD11  SLC25A1  LLGL1  KRT14  LEPROTL1  UNC13D  ZNF253  ZWILCH  NIPSNAP1  PGRMC2  PCBP2  ZNF184  MEIS2  C12orf29  TUBB  CLBA1  CREB3L2  THAP12  GCKR  NABP1  KIAA0513  PITX3  DOT1L  APOOL  CYB5R1  PNRC2  AHSA2P  POLD3  LRRC27  OSGIN2  WRNIP1  YTHDC1  ROCK2  AIDA  PPFIBP1  HK2  CCDC25  CAMSAP2  FAM160A1  TAF13  UIMC1  PITPNA  KLHL8  HMMR  RNF135  EXOC3-AS1  RSL1D1  PNMA2  MYBL2  LMAN1  ZDHHC18  NELFB  STON2  CUL5  BLM  UBQLN2  ZFP90  CTNNBIP1  PRAG1  NBN  PCDH9  CCDC92  PPP2R5E  STEAP3  SYNJ1  GOLGA3  ZNF793  SALL1  MFAP3  SRSF4  RAB3GAP2  KLF7  GRN  MBTPS2  MFN1  PGAP3  LINC02593  VPS45  CDK1  COMMD8  RNPC3  SGK1  BCL2L2  CYB561  ABHD5  KNSTRN  PIEZO1  KDM2B  ICE1  CAV1  SH3RF1  PPP6R3  NUPR1  LINC01578  DVL1  USO1  ZNF791  PLCG1  SLC45A3  TOPORS  LIPA  NUDCD3  ORC6  CCDC50  KLHDC3  SNAI2  GNA13  ETV5  RAVER1  SEC61A1  BTBD3  COQ7  CCDC191  HOXB5  SLC7A11  CYP1B1-AS1  CHPT1  LINC00857  GTF2F2  INF2  CDC42EP1  PRR14L  CYTOR  CRY1  ATF1  USP10  M1AP  FAM199X  NMT1  ZBTB7A  NFKBIB  FAM168A  WTIP  SIRT5  ECM1  MIR100HG  GTPBP1  CORO1B  TNPO2  FLRT3  GNA11  SETD7  C1S  TTYH3  TADA2B  GPATCH3  KLF2  TBC1D13  ZCWPW1  YWHAG  DR1  CCDC18-AS1  PGM3  UGT2B7  PHKB  KIAA1109  VBP1  CCDC9  RALA  MCM7  CNTRL  RUVBL2  AHCYL2  ASF1B  COPS7B  PLEKHG5  TNFAIP3  NOL7  ADRA1B  DPH7  VGLL4  KLC4  DROSHA  C20orf194  TMEM254  MXRA5  HCCS  INAFM2  CKS1B  RNF150  CWC22  ID2  THNSL1  SGSM2  DTNB  HIP1  ZNF480  XDH  HOOK2  KDR  GRIPAP1  HLCS  SNCG  INTS10  PBDC1  BBOF1  BICD1  PER3  AC009055.2  TMEM30A  CCDC117  PLK3  LNPEP  FNDC4  CDK2AP1  SMAGP  CHIC2  KIAA1549  SIDT2  AC003092.1  IGFBP6  UBR3  TPGS2  GTF2F1  ZNF525  SNHG1  NEK6  TMEM51  KAT2A  PARP3  AC024896.1  HKR1  PARP4  ZNF135  DDA1  MTMR4  DESI2  MYO1D  AGPS  ZDBF2  IFT43  KLF12  CFH  UBE2A  WDR36  MYRF  PRPF6  TFCP2  LUC7L3  COQ4  AC145207.5  C15orf39  CEP131  KCTD18  PALLD  BAZ1A  FAM102B  PEG10  DHFR  ANAPC11  PNISR  ERN1  CGB8  KIAA1147  SMURF2  LATS2  SNN  WDR47  SLFN11  AL445490.1  SERINC1  SINHCAF  TMEM87A  KNTC1  SFR1  FAM129A  FBXO36  PRELID1  STX1A  NOCT  SCG5  FUCA1  SLC23A2  ANKS6  PHF7  LEPROT  TMCC3  MAPKAPK2  CDC27  TRABD2A  NUDT4  ECHS1  KLC2  BRIX1  PTCD2  MTIF2  CHD9  LYRM2  HIST1H1C  ALCAM  KCNN4  ZNF267  SYNM  MED29  EEA1  SIK1B  IMPA2  GPT2  BABAM2  RNF13  PTGR1  SEC31B  ANKRD54  SLC39A9  SESN1  B4GALT2  AHNAK  SECISBP2  ARID3B  POU2F1  H2AFV  GDF15  TFE3  BMP1  SLC39A7  AARS  TNRC18  APBA2  CARMIL1  VPS26C  ITGB8  F2RL1  AAAS  IL15  ARAP3  PSMD13  TOM1  CEP128  CEP290  CENPK  ZNF114  MELK  CDCA5  DDX23  E2F1  AP001372.2  TOP2B  CALM3  PLCB4  CPSF2  ARL5B  COX10  WWP2  RPL9  CBX1  MICAL1  RAD17  GTF2H3  THYN1  SLC35F6  FGFR1  ARRDC1  TOP1MT  TMEM92  PLA2G16  ARNTL  LINC02432  VSIG10L  HMGN4  BNIP3  ZNF609  IKZF4  GLO1  DNAJC6  GNAI2  CRISPLD2  NUP93  OSBPL2  OAZ2  NOTCH2  FLOT2  TNFAIP1  PSMB8  ING5  CAMK2N1  GHITM  RHEB  THUMPD1  RAI1  PGF  CC2D1A  UBXN7  GPR137  GPR108  FO681492.1  ALG3  CNP  ZNF776  KBTBD2  GTF3C5  GID4  NAA40  LRRN1  TMEM8A  ITGA6  PER2  ATP6V1C1  C4orf33  TEF  TRAPPC1  LRRC37A16P  TXN  USP8  MAP3K5  IL1B  EP400  TELO2  EIF3J  CYB561A3  NIT2  ZNF506  TBC1D31  PTER  ABCF3  ATP2B4  TTLL11  CHTF18  GDPD5  WDR54  ABCA1  ELOC  FAM8A1  ADAMTS15  SDC3  PRPS2  ZSCAN5A  FRG1BP  BAX  MCRS1  TERF2  CEP164  TFAP2A  EIF3FP3  COMMD9  CRLS1  PHLPP2  PANK4  MFSD3  LRSAM1  PDE10A  CCDC97  UBXN1  AC092171.2  PSMC4  NUDT21  PLD3  CPSF7  ACSL4  CFAP20  SEPT10  YBX1P10  FRG1JP  WNK1  CDCA7  NLRX1  AC111000.2  DEGS1  SIRT7  ZBED3  NTSR1  MPP4  TMEM237  CSNK1E  PAXIP1  SNHG17  CEP250  ST8SIA5  AKIRIN1  MAPK1  GLIS3  INPP5A  ZMYND19  TPRG1L  NEURL1  LDLRAP1  RRN3  FSIP2  SEMA7A  TCF3  DGKZ  KCNMA1  PHF14  NSMCE4A  SMYD2  ZNF706  NUP188  BLOC1S4  DDX11  DOLPP1  NECTIN3  SSR3  KMT2B  TOP2A  MTMR12  FBXL17  MTFR2  SPC24  TSPYL4  NUMA1  GAS5  TADA2A  NFKBIL1  F11R  MALL  IL7R  ABR  SEC23B  MIRLET7BHG  RPUSD3  CES2  EFR3A  MEF2A  E2F3  PJA2  TBRG1  MTCH1  BAG3  ZNF737  PIGU  SARAF  EIF3A  TMEM64  TSPAN3  WAC-AS1  GK5  MAX  FBXO45  AFDN  EXOC7  JMJD6  TNFRSF21  KIFC2  NFATC2IP  SH3GL3  AXL  NCOA4  TTC33  HRK  ZFP36L1  IKBKE  LAPTM5  PALM  FAM161B  RAF1  CAST  ZNF574  GLS  SYNGAP1  CDK10  RPRD1A  HTT  DBF4  SCEL  WARS  PLA2G6  PRPF40A  SPAG9  ZNF813  KCCAT198  SLC25A11  AL356234.3  ITPK1  SCRN3  CPA4  HSPBAP1  MKS1  FOS  IFNAR1  FANK1  APBB3  FNIP1  RRAGD  DDHD2  CFAP70  FAM171A1  SRSF2  ARPC5L  TSPYL2  TGIF2  SLC16A1  FAM43A  RIPOR1  DPYSL2  RBM24  RC3H2  ZNF229  RAI14  CHN1  DNAH7  JOSD1  LRCH3  LUCAT1  ZNF880  DOCK4  WSB1  SLC24A1  TLDC1  PHF8  RNF40  APLP2  FAM189B  GAA  H2AFZ  FOXJ3  AC005332.6  PTAR1  WDR31  GDF6  HINT3  HES2  FAM227A  BTBD2  RETREG3  TAX1BP1  C11orf68  TLNRD1  AHR  HIRIP3  PEX10  AL596244.1  BACH2  NR6A1  VSIG10  ZNF761  PMS2  SLC50A1  KCNK5  CNDP2  KLHL42  FOXP1  TMEM87B  AGAP1  DIMT1  TM4SF19-AS1  C9orf40  LEMD2  NRDC  SPAG7  UBXN2B  PTCD3  MSL1  CALB1  HNRNPH2  CDK12  SDHC  NUP155  CDC42SE1  PLP2  ZNF28  CAMKK1  ZBTB47  SMYD5  FAM50B  RGS22  ATP9A  GCC1  APOBEC3B  RBM10  LINC00664  METTL21A  COL6A2  ZNF302  TM4SF19  TMEM120A  SRP9  ERBIN  TPD52L2  MOB4  PABPC1  EPB41L1  HDAC7  LMNB2  EIF1AD  DLST  MACO1  JCAD  CLTB  RLIM  TRIQK  COPS4  PPP1R26  PGM2  STS  ZNF84  PSMB1  SLC25A43  ZNF25  SEL1L  RPL37A  RNH1  RDH10  NEBL  MST1R  ABHD14B  VPS72  ATXN3  CBX3  GTF3C3  BCAS1  SLC37A4  CPT1A  ESCO2  MAPKBP1  FUT11  ABCA3  TDRD3  SF3A3  ORMDL3  MAZ  PEA15  CELSR3  FBXL3  TRIB2  PCED1A  BAZ2A  AC083799.1  VRK1  LPIN2  CENPBD1P1  SLC41A3  ASCC1  PSMC2  ZFC3H1  MBD5  FAM172A  ACTB  MTHFD2  SAMD15  CENPBD1  MAPRE1  THOC7  FANCD2  AGBL5  ARFGEF1  NXN  PXDC1  RHBDF1  BX470102.2  IFT46  MLYCD  HDLBP  ARMC1  FBXL19  SMARCAL1  GM2A  CADM1  BSDC1  DLG5  ABLIM1  GDA  TMCC1  RASSF3  NUCKS1  TBC1D1  SBDSP1  MOB3A  PLEKHJ1  CUTA  BACE2  ACOT13  SIK3  TCEA2  SEL1L3  UBTD1  ZDHHC6  RTTN  TM2D2  ORAI1  ARRDC3  BLMH  TTC7B  SDCBP2-AS1  ALDH5A1  SLCO2A1  RPS17  NANS  GAL3ST1  MICB  CDV3  CTR9  TMEM170A  SNUPN  ARNTL2  NUDT9  GPR17  DFFA  DHRS11  MTMR9  RPS3  MAML1  CLCN6  GFOD2  TMX1  TLL2  AC112777.1  SPTY2D1  AP002761.4  KLHL21  ILVBL  BRIP1  TRIM56  RPL22  POLR3A  STAT3  LMBRD2  HS2ST1  LRRIQ1  TPX2  VAV2  RBMS3  NFIA  PPP1R15A  MAP2K7  PLS1  ALDH3B1  MYEOV  AGPAT5  PELI1  MED21  RAD23B  SHROOM3  AP2A1  AMZ2P1  HKDC1  KIAA0319  MTSS1L  ECSIT  ARID2  FANCI  ENO1  CREB5  NECTIN1  SLC12A6  CERS4  TTC12  KNL1  ATP6V0D1  KDM6A  ULK1  SLC47A1  ETFRF1  DPP3  CHURC1  HRH1  PRDX1  FBXL2  HEATR5B  PIK3C3  CHMP2B  RFC3  C12orf49  S100A2  DEDD2  MIPEP  GAPDH  BROX  JUND  EFTUD2  CMTM4  SUMF2  AC243773.1  MOGS  CA12  PSIP1  OGT  GPAM  FOXO4  SMIM13  GALNT11  TAOK1  VANGL1  PPP1R12C  DCAF11  PSMD12  TSC22D3  KLHL12  POLR2L  TEX264  PMP22  SELENOS  NUMB  CCDC159  DAW1  NKAPD1  PPP1R16A  COPB2  ZMYND8  ARL10  IMPACT  KCTD10  DYNLL1  NHLRC2  FOXA2  AKAP17A  CES4A  PPP1R9A  SRP72  RFLNB  DGKA  JUN  PAQR3  NBEA  LARP1  DHX58  TMEM198B  RAB3GAP1  HOXB6  SPRED1  KRT17  SERBP1  SH3BGRL3  SPACA9  CEP89  USP32  SLC35E4  ZNF204P  DUSP10  RMDN3  IRAK1  PCGF3  CPNE1  CLN8  NUAK1  ZNF724  HDGF  CTPS1  AC240274.1  MAP3K20  SUMO2  SUN2  SP3  GSTO1  ZBTB39  HNRNPAB  GATB  SMARCA2  LHX1-DT  SRSF7  MFSD4B  ERGIC2  HERC5  CLCF1  RPS27A  DNAJB12  FGD6  MCRIP2  PRTG  ARHGEF12  CAPN5  SCAF11  GARS-DT  KDELR1  DPY30  TTC3  PABPC4  GNAQ  EIF2S3  TEX10  VPS36  C11orf95  ZNF331  ZNF280B  RAD51C  EXTL3  BEND6  ZNF121  EVL  OSBP  CGB5  TCEANC  SEPSECS  AMDHD2  HMGCR  TKT  ARMT1  PROB1  PRX  HK1  MBD6  TSPO  RCAN1  NIFK-AS1  THAP11  C16orf91  ADAM12  MRPS26  RIPK1  MLXIP  COX8A  SMARCA4  APOL3  HSD11B1L  PRAME  GPR15  PPP1R3B  NSD2  STK11IP  MCCC1  ZFP36  CCHCR1  CDC6  VEZT  NEIL2  TMEM132A  VDAC1  CRLF3  MSI2  RALGPS1  EML1  MAPK8  AC245297.1  SLC16A2  MAFK  CSRNP2  ZKSCAN8  ZNF268  SREK1  TMOD2  CHST7  PRXL2A  DNASE2  5-Mar  TBL3  BDP1  VPS52  WDR82  FDPS  SMARCA1  NCBP2  SAMD4A  ZNF189  PARG  TERT  AC007996.1  VCP  KCTD2  ZNF486  MARCKS  RBM12  NDC1  ZSWIM1  RAB11FIP3  MAPK8IP1  ZNF181  AHSA1  GPAT4  DCDC2  USP42  ARHGEF3  PPP2R5A  MIER3  TMEM201  SMYD3  CALCOCO2  TMEM214  SNX6  TECPR1  USP47  RRP9  KIFAP3  DGCR8  MAGED1  RABEP1  LINC01711  GTF3A  SNX5  STYXL1  CCNQ  MAPT  LARP7  GMPR2  GRWD1  SETDB2  RPL12  ZNF18  GNE  TMEM59  ADTRP  RTEL1-TNFRSF6B  TOGARAM1  LINC01322  THAP8  NID1  WDR92  PACSIN2  SLC7A6  TUBGCP5  SLC9A8  ZNF626  ATXN1  LINC02474  PIGN  FAM160A2  FOXS1  UBA3  MAP9  TAF15  PRKCD  SLC35A3  PHF20  UBE2N  ZNF74  GRB14  PIGV  ZCCHC17  ZC3H12C  ZNF702P  RHOB  PTPN21  CDC34  PEX6  PCDHA12  LINC00674  REEP4  NMT2  PCSK4  BET1L  XPC  MX2  FYTTD1  TRAF6  PHRF1  YKT6  VAV3  DHRS4-AS1  ST3GAL2  PPFIA3  ATP2C1  USP11  KLHL15  FCHO2  HPCAL1  RPA1  LAMTOR1  NECAP2  SKA3  PCSK5  APEH  FN1  SLC16A9  AC012313.1  DDX17  SH3BGRL  ZNF24  SGCB  PELI2  LRRC6  RPS12  PSMC3IP  DMXL2  IFT88  ZNF672  CSNK1G3  POSTN  FBXO5  CDK2AP2  FOXO3  DPYD  PROSER1  SH3KBP1  ABHD2  KIAA1958  TRIM5  TSC1  MEGF9  SMC6  CENPU  TAF4  CAND1  SNRPB  RASSF6  PLXNA1  NMI  MCM5  NT5DC1  UROD  STRN3  ZNHIT1  SERPINB5  KLHDC7A  PIM2  ABCD1  XPOT  SLC29A1  GCNT2  NUDT22  TOX2  PGLS  DOCK5  TROAP  ARFGAP3  SMCHD1  ASAP2  DDX52  MON1B  UBAP1  TXNRD2  CCDC24  ZNF140  ADM  AC016747.1  WHAMM  MAP3K6  SNIP1  FRK  HSPA13  C1orf174  PUM3  LSAMP  TMEM67  NRGN  AC060780.1  RAB27B  ABHD17C  DNMBP  ZNF517  ZNF514  SH3GLB1  PCYT1A  ZNF606  ZNF699  INVS  BLOC1S3  KRT80  VPS37B  MOCS2  QTRT2  COMMD6  APBB2  PIGO  TMUB1  TANC2  RNF167  PPARA  GPRC5C  PPIL1  MT-ATP8  COL13A1  RFC5  ZNF93  NANOS1  ANKRD12  TARBP2  LINC00894  CCSAP  MGAT1  INAVA  RPS21  ATRAID  RNF216P1  CACFD1  PRPF4  RTL8A  TCTN1  DCBLD1  NUDT12  C18orf21  PPIF  PLEKHM2  PREB  NUP35  TPK1  PAPSS2  THOC6  PHF10  GTF2E2  HCFC2  ATF5  BAG1  ARHGAP39  PRKAR1A  TCP1  ZFP91  SLC39A13  SSH3  DDX54  UHRF1BP1  TBC1D14  WIPF1  CARF  ZFAS1  GPKOW  SYNGR2  RELT  RSU1  RPS6KC1  DCLK2  FA2H  PSMA4  GPR107  GALNT2  WDR24  NNT  CCND3  ACAT2  MECOM  MRPL20  TNC  DSG2  AC011503.2  AP5Z1  POLRMT  MORN1  C9orf64  MTBP  MMRN2  MKKS  FXYD5  DCAF13  MRPS6  PIAS2  ZBED6CL  DDB2  SUPT6H  TCIRG1  ARHGAP32  TIMM21  BTRC  BIRC5  MRTFA  PIMREG  UTP25  DPAGT1  NFYA  RGS17  NCKIPSD  CRTC2  AC233968.1  ROM1  RAB11FIP5  ZNF623  UBP1  UBE2K  TROVE2  PIP4K2C  MPDU1  EGR1  TEX30  FAM72B  MED8  CAPS2  RNF14  RASSF1  AFG1L  CLIP4  KIAA2026  LRRC38  SNAI1  FEM1C  FOXO1  BCCIP  FAM118A  PDCD6  CORO2B  MIR222HG  TMEM154  PRKCE  FSTL1  SLC25A4  COPS9  C12orf76  GDF11  CD109  AC004585.1  WDR34  VPS25  SLFN12  SHANK3  SIAH2  NUP54  RRM1  RPLP1  PCDHGA4  OCIAD2  RIPK2  NLRP3  CHMP7  MELTF  CANX  ZNF624  SUGP1  SERTAD1  GGCX  TRANK1  COPS2  SLC37A2  ANKRD50  DHRS1  RRP8  CXCL2  SUN1  FAM201A  LSM14B  GAB1  TEX2  LINC01139  PTPN12  PATL1  COX15  C6orf223  NPR2  FNDC1  ZNF689  RPL26  TRMT2B  FAM124A  MMP3  ATP6V0A1  ZUP1  LRRC37A4P  HOXB13  SNX18P3  LRRC23  CPSF6  ATAD3B  SLC9A3R1  ZFP28  ITM2B  TBC1D9  C1orf52  EIF2D  CDK20  XYLT1  TICAM1  ARHGEF26  CAVIN2  ZNRF3  CXXC1  CTDSP2  CTXN1  SNX8  NCSTN  TAF4B  MVK  PARP6  PLPP1  DNTTIP1  MCUR1  EED  TPP2  ZNF85  SP110  LCOR  ANKRD10  MEIS3P1  SUV39H2  AL133216.2  PHOSPHO1  IPO7  TM9SF4  PPP1CC  YJEFN3  MTHFSD  RNASEH2C  AGA  CCNL1  AP2M1  AHNAK2  YIF1B  CCP110  RRBP1  CAPZA2  AL513550.1  PCDHB8  CDKL5  CBX5  OPA1  ZNF716  SPATA24  ZYG11A  COL17A1  DCTD  ARMC8  ZFR  VPS13D  SLC38A10  EHD2  RIT1  QPRT  SPEF2  ZNF185  CCNJ  MST1P2  KCTD9  RPS23  ACAT1  VPS13B  EIF3D  RPL24  PYGL  INTS1  COL16A1  SNRK  SLC4A4  ITM2C  TAGLN2  CAMK2G  TGFBR3  MAN2B2  CARM1  SPRYD3  IL33  IFT52  HNRNPH1  TNFRSF10A  NDE1  FBXO7  POLA1  ELOVL6  SPRED2  IFIT1  AP2S1  ZFPM2  GSN  PSMB4  MRPL27  PDK1  CIAPIN1  CTSH  YEATS2  NF2  AC026401.3  LINC02241  NOVA2  SOD2  ARMH3  NBPF1  OBSL1  UBA52  SCFD2  PGGT1B  ZNF35  AKAP9  FAM98A  TLE3  USP7  KIF9  UNG  ARRDC4  FBXO22  GLG1  KLHL2  VPS41  RAB28  MCUB  HMGB3  TFPI  ZNF528-AS1  PGRMC1  LINC02434  PKN2  RNF157  PHF19  LRP6  HLA-B  GFM2  CYB5R4  AMOTL1  DNAJA1  MANF  TRIP11  ITGAX  MNT  EAPP  SOWAHC  CCDC144B  RPGRIP1L  CIB1  STK11  ZDHHC4  MAF1  EVA1B  AK8  ACSL3  HAUS1  HCN2  RMI2  COL4A3BP  SLC25A46  ZNF836  MIR155HG  HIVEP1  HIC1  IGFBP4  NIPAL1  IKZF5  RBM38  PCDHGA10  AOC3  ASXL2  TMEM170B  STAMBPL1  SEMA3B  CD46  NAA50  ASB8  PEX11A  MTX3  SFXN1  ZNF704  RRAGA  NCAPD2  ODF2  DAP  ZNF510  SPATA20  ANKRD28  TTC37  GRB2  MED9  FOXE1  AC015813.6  AL590428.1  GRAMD4  UBE2G2  WHRN  CBR4  VAMP7  UTP4  SPATA33  P2RY2  BMP2K  RGS9  ZC3H12A  TMEM167B  MEF2C  YWHAZ  TGFB1I1  CD3EAP  OTUD7B  ZNF17  LINC01127  H2AFY2  XIAP  TERF2IP  SLC4A7  SLC25A37  OGDHL  MAST4  BPTF  ELOVL1  QSOX2  GUCY1A2  HFE  UHRF1BP1L  NME7  MEX3D  IL36B  BSCL2  PIAS1  ACTR10  LHB  ATP6V1E2  HSD17B4  TRIM29  POLR1B  TXNL4B  LIN9  ATXN2  GBP2  MARCKSL1  TFRC  TNFRSF25  SNHG4  ZNF362  ZNF708  RAP2C  DNAJC21  TEAD3  LRBA  ITFG1  FOXI3  IQCE  STIL  TLR6  KCNK1  LINC00662  MRPL58  SIX4  PAQR5  MAPK8IP2  KCNAB3  IFT122  SPTAN1  TSNAXIP1  PPT1  KIFC3  DENND2A  THUMPD3  DDR1  TMCO4  CRYZL2P  SH3D19  HYAL2  KCTD12  DHRS13  IGFBP7  PAN3  WDR43  DNMT1  SERPINB8  UACA  ASCC3  PINK1  NAPG  MED27  EIF5  SLC26A11  RFK  ZNF589  NRIP3  RPLP0  LMF2  ENSAP2  PDSS2  MCOLN3  ATG16L2  KLHL22  FXN  DLD  MLF1  BX640514.2  SNHG19  PLAG1  ADAM19  DCHS1  HARS  PEX11B  ACVRL1  MAN2C1  HLA-C  CITED4  IL1RAP  IFT74  ATG4A  NCOA1  MBD4  FN3KRP  AC105277.1  BRI3BP  KIF14  RBM33  CCDC106  MCU  M6PR  PRRT1  STRN4  MICU2  MPP5  APP  LINC01293  KIAA1324L  LAMP1  DDX39A  MGAT4A  CTSL  FAHD1  ATF3  NUB1  AC008736.1  GSK3A  MARK4  TWNK  ASB6  FAM126A  GNB5  POFUT1  RNF141  HTATIP2  ELOB  ATP6V0E2  MC1R  ARL16  PCTP  ALDH1A3  CNOT8  MRPL10  DYNC2LI1  ERLEC1  TCEANC2  PUM2  DDX3X  ZBTB34  SIL1  EDIL3  AL391244.1  CABLES2  SYNPO  RETREG2  MRPL32  AC019069.1  C1QTNF1  WDR25  IK  PFKFB4  MSH2  PADI1  LINC00707  ARFIP1  AKTIP  TDO2  ERV3-1  UST  ROR1  PTK2  RPS15  HECTD3  MFSD2A  FAM122A  H2AFY  HDAC4  UBAP2L  RAD51D  DDAH1  PCGF5  KIF1BP  NR1H3  LRRC40  PITPNB  TMEM104  ZNF274  NUP85  IGF1R  LINC01559  ATG14  SDC4  DNAL4  MMAB  AC006058.1  CYR61  LPAR3  PRPF38A  SLC25A10  PEX16  LINC02274  KIF20B  ETFBKMT  LINC00342  LSM1  SYNGR1  Z94160.1  FAM111B  PGPEP1  ZNF729  GPX1  MYBL1  FNBP1P1  SELENOO  RGMB  HNRNPC  KIF16B  FADS1  NCBP1  SBDS  AC124319.2  ZNF658  RWDD2B  INTS12  PCDHA1  TMED5  TLE1  SLC25A20  AP3M2  SLC2A6  AP5M1  ATP1B3  DIP2A  UGCG  SHANK2  PINX1  DDX39B  CEP192  DNAJB1  ARHGEF7  FANCB  P4HTM  PTPRR  PXMP4  FLNB  ERG28  TTC39B  EBP  SHQ1  ELOVL5  ZNF585A  HLA-A  CAPN15  ZMAT2  NUP205  AEN  TIMELESS  RPL10A  GSTCD  MAP4K2  KCTD7  PLCD1  RAB3IL1  PIGZ  CDC73  DNAJC18  EPB41L4B  ZNF638  SF3B5  CIP2A  LTO1  USE1  SOX9  SLC2A11  ASPSCR1  CRYAB  TRA2A  SOCS1  FICD  SRP14  UBE2I  DUSP22  ILF3-DT  GPCPD1  BRD3OS  ZNF507  PPP1R13B  LMBRD1  GABRE  PMFBP1  TAF7  RB1  TNFAIP8L3  OSBPL11  BCKDHB  GEMIN4  PNPT1  ZHX1  SKP1  NRL  PTRHD1  TRAPPC11  HMGN5  AC090673.1  SLPI  VPS33A  NIPSNAP3A  DCAF17  RUSC2  PPP1R3E  SLC39A14  HSPA4  HMGB1  CAMLG  ABCC3  AJM1  TRAK2  STK36  AC078883.1  CPS1  SPAG8  XRCC3  WDR81  VLDLR  ESPL1  AC116533.1  MTIF3  GLRX3  MCTS1  MX1  ATF7  IFI16  AL133367.1  BTN3A3  SLC25A30  ZRANB2  GNGT2  TM4SF18  PHF5A  ICA1  WNT7B  DCTPP1  VPS18  PTMAP5  LCA5L  SLC38A1  UBAC1  NEO1  WDHD1  CCM2  POP5  GPD1L  FAM208A  HAUS6  SP2  PSMG4  C3orf58  FANCG  SPTLC3  BMT2  HERC1  ZNF559  RNF20  HNRNPD  ZADH2  RFC4  GPATCH11  FBLN1  ZNF263  KCNC3  YPEL5  PFN1  UTP3  DLG3  DGKE  NME6  KRT19  NPLOC4  PATJ  TRIM2  WARS2  ZNF676  C19orf48  SPRY1  TCF7L2  SLC35G2  SON  ARHGEF25  ANKRD9  ZCCHC14  PCBP1-AS1  ZFYVE26  SLIT3  ATP6V1B2  ZNF783  ZNF484  NPIPB12  HNRNPF  ZNF879  MYZAP  TALDO1  LOXL1  GGH  GRAMD1A  ADO  CD82  IRF9  CST3  STN1  HEXIM2  ZNF717  MLLT3  MYH15  EMC3  GHDC  ATP6V1F  WDR26  SAT2  SMIM19  ZNF98  TNFRSF11A  CHMP3  TRAPPC12  ZEB1  ZSWIM9  BACH1  LCMT2  NDUFB2  TUBGCP4  CHP1  WRAP73  PKD1  CDADC1  MSANTD2  CPSF3  ANKLE2  ZNF528  ZFP62  MRPL54  SLC25A40  NSF  TPRKB  SUMO3  ZNF675  AGGF1  FAM192A  EBPL  RBM22  RSAD2  ARTN  DLGAP5  ZNF280C  NBEAL1  AC139769.1  TWF1  ARMCX5  B4GALT4  NOP58  SAP30L  EIF4B  DEF8  FOXL1  CRMP1  RNF144B  PBXIP1  AC060766.1  OFD1  RPL7  PWP1  UBN1  MAT2B  GRAMD1C  LONRF1  ZNF780B  OSBPL3  TBC1D24  AC011379.2  VPS54  ARID1A  GOLPH3  RUSC1  GMCL1  ATP6V0B  ZNF600  ASB1  CLDN4  CLGN  RNF130  CAPNS1  SLC39A6  AC073861.1  SMPD1  KLHDC9  PRMT3  USP46  VPS9D1  WIPI2  MIR22HG  MED16  TPST2  MSRA  CETN2  DPCD  AC060766.7  HDAC1  SRPK1  AC084033.3  ALDH18A1  ALMS1  RAB30  SRSF3  COX11  SAP30BP  KLF4  THAP7-AS1  ANKRD10-IT1  MAPK13  SORBS1  C17orf53  GGPS1  RBSN  TBC1D8B  ARHGAP19  SYT15  NUDCD2  SLC12A3  ALG14  RPS28P7  RBM44  2-Mar  PRKCA  FAM207A  MXD1  TRMT10C  MED28  NCAPG2  ZNF329  ZNF876P  TLK1  FMNL3  LYRM7  BTAF1  ZNF512  ARFRP1  KIAA0930  PCBP4  ZCCHC4  FAAP100  NBL1  SLBP  ZNF343  WDCP  MUC5AC  OASL  CLYBL  ANGEL1  PHGDH  CLEC2B  PCDHB5  RAB20  MED10  RAB29  ZNF285  FAS  LMNB1  POLR2E  MME  UBQLN4  GBP1  CDKN2C  ABCC1  PKP3  CHCHD2  RRP1  LINC00620  TGFBR2  METTL1  PPM1D  ZNF141  TMEM175  MKRN2  GLI3  TSPAN15  PCGF2  PTPRM  ZMYND11  LINC00847  TMEM203  SNRPA1  COL4A4  FBF1  CYP20A1  USP36  PRIM2  MAP1A  NEPRO  STK16  TMUB2  FEM1A  IGFL3  RALBP1  PC  DARS  SH3BP5L  MGC12916  SLC9A3-AS1  EIF2AK1  GMNN  SF3A1  ZFYVE1  BST2  REC8  TUG1  TSPAN6  YTHDF3  TIPRL  ST13  CIAO2A  SYT7  SCRIB  RRP15  MYBBP1A  KIF21B  SND1  ACVR1B  AK5  GOLGA5  NOL9  TRMT1  ADAT1  TACC1  WDR41  ZNF582  TEDC1  FTO  RPS29  GJA1  NT5DC3  IL6ST  ELAVL1  FOCAD  RPS27AP16  SCN8A  LONP2  MUC1  APBA3  HSP90AA2P  RPS6KL1  MIF4GD  MYPOP  ASB13  RALGAPB  MEGF6  XAF1  CLIP2  CTDNEP1  NCKAP5L  IFNGR1  PAGR1  OPA3  CBX7  RBM14  DCLRE1B  CNOT3  CCT6A  AK4  ZSCAN20  AL513327.1  CWC27  SYAP1  ADGRL3  STOML1  PURPL  SLC35E3  CHD7  MGST1  UBLCP1  NATD1  MBLAC2  TOMM20  UBE2S  CD47  Z95115.1  PIP5K1C  PRXL2C  EDNRA  NARS  IFI6  FAM122B  FLNA  DIP2C  NDUFA5  TBK1  FRMD3  TSNAX  SH2D3A  KCTD15  LARS  ORC5  WASHC5  NKRF  SAT1  NUPL2  NPAS2  SPOCK1  SAMHD1  SRSF1  UBE2V1P2  RPS28  ATP5MC2  CPT2  RFC1  NUDCD1  TRIM69  RPS19  SNW1  CRYBG2  FAM214A  FUZ  PSMB5  LRP8  BYSL  PDHB  ZBTB2  BCL2L12  NGLY1  CHD4  C6orf106  POMGNT1  ZNF221  CLDN2  SARS  PRDX3  PDE12  ASAP3  TBCE  TRIP6  SOAT1  FUT4  AIMP2  ADAMTS5  HINT1  SFTA1P  FAM161A  GVQW3  ESYT2  MAD1L1  ZNF432  GNL3  BAG5  TIA1  AC115223.1  GCOM1  FTSJ1  WDFY2  MR1  LTBP1  TXNRD3  RTCB  ELMSAN1  FBXW9  BRWD3  UNC5B  PGP  DBF4B  PTK2B  ARFIP2  IFI27  MRPL40  SHROOM1  ARHGAP1  LRP4  SLX4IP  ZNF414  ANGEL2  PRKAR1B  PSMC6  SLC1A3  ARPC5  STUB1  LIMCH1  MPP3  RBMX  RPLP0P2  CALHM3  PQLC2  MIR137HG  TRAF3  GTF2IP7  ZNF701  SLU7  FKBP7  ADCY10P1  ACSL5  SGF29  SLC35B2  RRS1  TMEM242  C19orf57  VPS26B  PIGX  CHAC2  AL355338.1  CPNE8  ANAPC15  EML3  BX322234.1  RSPH3  CHD3  PANK3  GBA2  DNAJC27  DMRT3  MAP3K8  RNF185  METTL4  MRPL35  FBRS  C19orf66  RAB8A  HEMK1  Z68871.1  GSDME  SLCO3A1  ARL6IP6  RBM19  RPIA  ZNRF1  ROBO4  CHRNB1  METTL22  ABLIM3  TAF1  SEMA4C  TRNAU1AP  MYL5  FBXO2  GEM  ZC3H6  PHF11  GCA  CNOT6  AL365181.2  PSEN1  ACACA  AC007325.4  MAPK14  SGTB  CPLANE1  POLE4  AC015912.3  MANCR  KATNB1  ZNF385D  AKR1A1  TTC3P1  FAH  PDGFA  AC022400.7  RYBP  PCDHGB2  AC123912.4  AC087741.1  UBE4A  VASH1  TMEM171  PRRG1  METTL17  SAFB  LRRC37B  PCDHGB6  ASAH1  KIF17  PLCD3  FAM162A  TMCO3  STAT2  ARL2  NAPA  ARHGEF28  TIRAP  SHOC2  ANKS1A  CD58  GART  C9orf116  ZBTB45  TTC9C  PHYH  AC105460.1  SNTB2  TPT1P5  ATP13A3  ZNF680  HADHB  SNTB1  RNF187  LYST  AL592183.1  EPHB1  USP16  UBALD1  MYCBP2  SUSD6  AGPAT2  FHL2  TVP23B  STK26  ZNF197  PAXX  CCZ1B  MRPL14  PIGBOS1  CAVIN1  DECR1  DBNDD1  ATP8B2  ZNF488  NFKBIA  TRAF5  RCBTB2  UXT  FBXO28  ICE2  CLCC1  CIAO1  HOXB7  EXOC6B  ADD1  SCOC  NUDT19  RPS20  SRSF12  ARPIN  MRAS  ZNF658B  HNRNPA0  TMEM185A  HMGB2  HMOX1  UNK  CYBA  DCUN1D2  PEX5  CLIP3  EFR3B  MAGED2  CENPQ  RSF1  TM9SF2  APOL2  AP002784.2  MMP24OS  ANO7  RHEBL1  MATN3  SPEF1  USF3  HSPA9  LINC00443  YIPF3  TRPC4  UBE2Q1  MRPS9  FRG1HP  MTOR  CCDC58  ZNF780A  TULP4  SLC29A4  DOK4  UQCR10  PTGES2  RPLP2  ENOX2  ZNF354B  ECT2  SRSF8  MYO5B  LYRM4-AS1  KIAA1191  MINCR  SDHB  HOXB9  S100A3  UFD1  PREX1  TIMM17A  AL022322.1  AL161891.1  LINC00942  NAP1L4  KIAA1841  ZFX  RPS2  PRPSAP2  SNHG5  UNC119B  CRNDE  HIGD2A  DLX1  NGRN  GRPEL1  JRKL  NRP1  TRIM62  GAS6  ZNF257  RTF1  PROS1  PACS2  AATBC  YIPF6  TCF12  ANKRD13A  SPAG16  SYTL2  DNASE1L1  MBD1  HYLS1  DOK3  APOE  MZT1  ZNF12  CHD8  GTF3C6  PRKCSH  KIAA0319L  EXOSC9  CMTM6  CTDSPL2  H1FX  AC048341.1  SLC9A6  PATL2  N4BP2L1  PHC3  ANXA8L1  NINL  PCDHGB3  SLMAP  BCL2  POLL  C21orf58  AC092910.3  NEIL1  RAET1E  7-Mar  RPS24  AL606760.1  ZNF174  AC005520.2  MFSD11  CCDC110  CCBE1  PSMD7  RPE65  NRF1  REPS2  EIF6  CCDC51  URB1-AS1  ISG15  SDCBP  SOS2  SPAG1  C6orf89  CIT  XPO4  PRKAR2B  SMURF1  EIF4E2  PPM1H  SIPA1L1  TBC1D20  ECSCR  FAM78A  FAM45A  SS18L1  LATS1  COG1  MRPL23  TAZ  FAM57A  ABCA2  SEC24C  LIN54  RANGAP1  XRCC2  NOL10  YBEY  CYLD  DCP2  MOSPD3  MACF1  L2HGDH  TMPRSS5  HECTD4  NR1D2  GPSM3  DCAF12  MTMR1  PTBP1  ARMCX3  PPP1R21  LUC7L2  RCOR1  LONRF3  SDHAF1  AL365181.3  TMEM184A  TRPM4  ASXL1  KDM1B  SAMD8  CS  LHX4  SRD5A1  PEMT  UNC119  UBR5  ANKRD34B  ABI3BP  ZNF211  CLSTN1  PITHD1  COX17  TMEM205  RNF10  MAP4K3  TTC21B  KLF16  PPP2R1A  RPL23AP82  MIR1244-2  BBS10  PDXK  OBSCN  RPARP-AS1  HIGD1A  GPR157  KAT5  VWDE  MPP2  PUS7  GS1-124K5.4  U2AF2  CHEK2  DENND1B  CASP8AP2  FAM174B  C16orf87  KHDRBS3  BRSK2  PCDH10  HPRT1  ZNF322  POMT2  MARS2  IRF7  CHMP2A  THUMPD3-AS1  BRD7  NME2  SPATA2  UBE2F  FAAP20  FAM204A  MAP3K21  CSNK1A1  GNAI3  RHOV  FAM120AOS  RNF25  TRIM65  CGGBP1  CALCOCO1  PUDP  AC010343.1  CARD19  LVRN  LHFPL6  SYF2  GPN3  MTA2  INTU  FTSJ3  TKFC  HELQ  MPC1  MED13  MAD2L2  ATP6AP1  FAM160B2  DHCR7  OXLD1  DAXX  AMY2B  APH1A  TAOK3  FAM53C  SCAF4  TP53INP2  REEP2  FZD7  FGFR2  GAS6-DT  ROS1  RACK1  INTS13  AKNA  GDF9  TNFRSF13C  ZBTB4  S100A13  GSTP1  PCLAF  TIE1  NDUFAF7  MEX3A  SHISAL1  PDHX  KNOP1  PEX11G  RPL34P33  HAVCR1  IFI44  OSCP1  CARD10  MRPL45  WDR90  HM13-IT1  CRYBB2P1  CEBPZ  HPGD  RPL23  AC010186.3  ARHGAP24  CADM4  MCM2  TSPAN4  STT3A  HS3ST1  ADAMTSL4  WDR76  ZNF891  FHL1  PTGES3  COPZ1  MAPK11  THBS2  IRF2  CHAC1  RPS13  L3HYPDH  GPR176  LUZP1  UBL3  HADHA  SLC33A1  REEP5  ZNF182  BAG2  MLX  MIA3  NIN  PSMB6  TMEM151A  CDH24  NDUFAF3  LPCAT4  MT-TN  ZNF800  GLRB  RRM2B  TET3  CTNNA1  BET1  GATAD1  RPL14  RRP1B  BEND7  TP53BP2  HECTD1  SVIP  AL136295.7  IDO1  UGGT1  MARK2  AC016831.6  ARHGAP35  MT-ND3  ADCK2  ZFP30  THEM6  PLEKHG3  ZNF734P  FNBP4  WNT3  LRWD1  ANKRD18A  LINC01411  NRG1  RNF19B  ADAL  COPS7A  NPC1  WDTC1  ERCC3  ETHE1  VCPKMT  MMD  CRAMP1  ZNF670  RFWD3  GCC2  ZNF470  ZNF311  FARSA  FBXL18  NPIPB3  PREPL  APPL1  SCARA3  FN3K  EXOC8  PNPLA6  PSME4  ACTN1  MVP  FABP5  PIP4K2B  NAGK  DSP  FBXO4  COG5  RARS2  SMARCD2  RASSF7  LIN52  EIF4A1  PCCA  ATF6  TWIST1  ADCY6  FXR2  RAD52  SPECC1L  FAM86C2P  SMDT1  AAR2  TSPAN17  ENY2  PRUNE2  ABRACL  EXOC1  C8orf82  SEMA4F  SMARCB1  NDUFA8  MLPH  UHMK1  PTS  PDXDC2P-NPIPB14P  MAVS  NAPB  DTX2  USP20  LY6E  SLC45A4  MLF2  FOXD2-AS1  AC026412.1  PLEKHF2  POR  SPOPL  ANKRD13D  CXorf38  KDELC1  FAM107B  LINC01629  TJAP1  NXNL2  MEF2D  TRIM16L  EIF4A3  MOSPD2  C1orf226  AKR1C2  CIRBP  SLC18A2  AL136164.4  TSPAN2  AGPAT4  RPL39P3  PSMD14  GPR180  WNK3  HDAC11  CASC8  KANK1  LETM2  AL391988.1  CYB5B  TMEM268  TFB2M  WDR45  GOLGB1  MRPS18B  COA4  SLC9A7  AUNIP  AC017100.1  CFAP410  PWWP2A  AC068946.1  THAP6  CCDC32  C3orf67  FADD  AP3S1  HSPD1  SDF2  KMT2E  ERFE  RECK  FHOD1  TMEM50B  SDHD  YIPF2  MFAP5  PPM1K  MIR193BHG  UBE2L6  DOCK1  ERCC4  SMAP1  WDR78  GTF3C2  FBXO34  IFITM3  NDUFS8  NAGA  ARHGAP30  FAM89A  DDI2  NREP  PCM1  CATSPERG  ICA1L  LINC01569  RGCC  TMEM53  UBE2SP1  ZNF112  GOSR2  DNHD1  HPS1  OSBPL10  SCGB2B2  CCNDBP1  NEMF  NPAT  RTN4R  MZF1  NLN  TNFRSF9  CTDSPL  MGST3  RNASET2  TUBAL3  STMN1  UPRT  ITPKA  SCHLAP1  BAZ1B  FERMT2  SPATA18  CALM1  LBR  ZKSCAN5  EMILIN2  C22orf39  SEC61B  ABRAXAS1  FERMT1  AGO3  FBXO46  KAT7  BRCC3  LYRM9  AC102945.2  SYNDIG1  MFNG  FBL  STEAP2  HSPA4L  AHCY  HAUS2  AL354719.2  IARS2  HS3ST3A1  FLVCR1-DT  CNOT4  MANEA  CLMN  DDX42  C11orf1  STARD13  WSB2  ARMC6  DPF1  TCEAL9  SRRM1  MT-RNR2  GMPS  IFI44L  RPL31  ANXA11  GPR161  AC010343.3  EMILIN3  DRD4  TMEM132B  SLC16A1-AS1  HID1  TRIM16  AFG3L1P  DDX12P  IMPAD1  LRGUK  RAC3  CDC45  HASPIN  CARS2  F2RL2  RAB22A  AMZ2  ENO4  RAD51-AS1  OTUD3  COG3  HNRNPA1  CLUHP3  NDUFA13  TBC1D16  MEN1  ACOT7  FEZ2  SDSL  CRELD2  ZNF446  LMNA  ZBTB22  NUP88  L3MBTL1  AC011451.1  BEND3P3  LHFPL2  CNBP  BLOC1S5  AP006623.1  RPL35A  C6orf99  HPS4  AC004656.1  UBE2J1  CC2D1B  ZNF883  AC009950.1  GPRIN1  CFAP44  TMCO1  FKBP3  AC022240.1  XRCC5  TMEM248  MCM6  QDPR  BCL2L1  CKS2  RAD1  TTC30A  MAGT1  SFPQ  NFX1  AP1S1  NRARP  TRIM68  ABCF1  AP1AR  KCNG1  DPM1  IFIH1  UBE2E1  TRIM36  FAM120A  PIM1  CRIP2  MAP2K5  RNF115  ULBP1  QRICH1  SP100  TRIM24  PCCB  EPAS1  PRKCH  NOP16  NDFIP2  STXBP3  GATD1  EIF3H  ADAR  ITPRID2  ARID1B  PPP3CC  EPS8L3  TMEM44  BRCA1  LINC02577  ZNF491  TMEM159  SERTAD4-AS1  PRLR  KCNIP3  TRAF3IP1  CPSF4  GNB2  THAP10  GNAI1  PCID2  AL359551.1  BEX3  DOCK2  JADE2  OAS2  POLR3G  AL136985.3  PRKN  AMFR  SMS  SEH1L  SLC38A9  MIER2  TTBK2  LINC01511  FCF1  VPS13C  LRFN1  WNT9A  RPS5  ELK4  DSCC1  NUBPL  EPHA5  GTF2IP20  DNAJC4  STX16-NPEPL1  TBC1D32  MMS19  MINDY2  ATP10D  USP19  ZNF688  KLHL14  ZNF99  STX11  SYT11  FBXL15  SLC19A2  GDAP1  LRRC20  PLEKHG1  AL355001.2  ITGA4  FAM241B  EPHB4  SCAF1  UCK2  CERS2  ZBTB12  ADAMTS13  WAPL  NAMPT  RXRA  AP002990.1  FBXL16  NINJ1  PCAT7  MYORG  ZNF251  AARS2  PFKFB3  ATP8A1  CEMIP2  PF4V1  MBP  GRHPR  GATAD2A  LINC00520  DZIP1L  PFDN4  ARHGAP26  SIVA1  ZNF286B  ATP5MC1  LNP1  HLX  MN1  OLFM5P  GAB2  ZSWIM5  CDK19  ZNF440  TMX3  FZR1  BCR  PAFAH2  RARRES3  CHIC1  ADORA2B  MORN4  CENPT  SGMS1-AS1  GNPAT  LPAR1  AC135977.1  DCAF15  IPO11  HERC2P9  ERMAP  MRPS36  ING4  SPIDR  CPM  RILPL2  TMEM161B-AS1  HRASLS  ARRDC1-AS1  TMED7  TRMT1L  TMTC3  RNF146  HIPK3  ZNF226  MXD4  LRRK1  EXO1  FP565260.1  JARID2  ZNF827  DNAJC1  METTL27  FRRS1  CUTC  RHBDD3  TLE4  MAFG  PLOD1  NPIPB4  NBDY  ENKD1  AC006058.4  GORASP1  SPEN  PARP9  TMEM107  STARD10  ZNF790-AS1  DENND4B  PJVK  ZNF852  EBAG9  DHX38  FNDC3A  UTP15  BOK  ZNF234  FAM234A  SH3GLB2  NUCB1  PTMS  USP1  GLB1  SPRYD7  ARHGDIB  CHORDC1  RSBN1L  ACSL1  LARP6  RECQL4  KRT86  ZNF75D  ZNF273  HEATR3  PROCR  PARP1  ARHGEF40  CPLANE2  ADGRG6  RECQL5  CDYL2  KIN  SRM  UNC5CL  IFT80  RPL27  HAGLR  B2M  GNS  ZNF595  FAM53A  OCEL1  UBE2G1  RNASEH1-AS1  STAMBP  MPHOSPH9  IKZF2  TRPC1  C1orf109  IQGAP1  NEB  MTND2P28  AC008467.1  ECH1  MAT2A  CDC5L  EMP3  NAGPA  TSGA10  SMIM10L1  FAM45BP  HAGH  GCFC2  ZNF790  SAMM50  SCCPDH  IKBKG  KIF3A  RPL8  SLC24A3  TEAD4  CSRNP3  CLIC3  METTL9  MCMDC2  RELL1  ZNF784  ULK3  TMSB4X  TBX1  DHX9  RHOG  IQANK1  FZD5  B3GAT3  RNF6  PRDM5  ODC1  RBBP4P1  AC011462.4  AC117402.1  AATF  CCDC144A  PAAF1  RGS5  SCN1B  FBXO11  LINC01705  TENT4B  CHMP4B  ZNF436  PLK1  DUS3L  MOB3B  NPHP1  MYNN  AKAP11  PDCD6IP  MAGEF1  ZNF90  ISY1  ARPC2  LINC01089  TRIM26  USP9X  RPGR  SNED1  KRT10  TRIM38  KLHL35  EHMT2  ZNF846  FBXW10  MCTP1  POPDC3  MIR181A2HG  DBNL  PTP4A2  SYNE1  TNKS  EEF1AKMT1  KCTD4  MT-ND1  EPG5  FOXD1  CCDC91  GOLGA1  FOXD4L1  MAMDC4  TMEM9B  AC079062.1  CASP9  ASIC1  CCDC6  SPG11  ATP2B1  DNAJB9  AL031714.1  TRMT61B  ARMC5  RCE1  FAM111A  DCLRE1C  ANKRD34A  ACTN4  CDIP1  MYEF2  AL590666.2  AC087612.1  AHRR  SNHG15  ADSS  PIGP  ZNF561-AS1  MDH1B  RAB21  ZCCHC10  YY1AP1  TOPBP1  USP31  BCAT1  RIMS1  ANKRD20A19P  EDEM2  PSMD6  LGR4  PLA2G15  CHAF1A  G6PD  TNRC6C  SDE2  WDR17  SH3TC2-DT  ONECUT2  ADAMTS6  LINC01748  ZFAND1  AP000769.1  GGT4P  CEP78  SPPL2B  PISD  ZNF554  ZNF839  PITPNM3  INIP  ZNF552  DNAH1  LTBP4  NOMO3  PKD1L1  RPE  LIMD2  PCDHGA7  C17orf97  KIAA0040  AP001362.2  TCEAL3  TIMP3  KIAA0232  DDX1  MAPK9  ZNF382  INPPL1  PRDM11  ANAPC16  SDF4  FKBP14  POGK  ACP1  FEM1B  EZH2  SETD1A  MRPL22  UBL5  EIF5B  DHFRP1  WDR11  LRR1  SNRNP40  NBPF12  BTBD11  SF3B3  MAP2K4  NNT-AS1  AL139260.1  MRI1  KCTD14  FMO4  POLR2G  CIAO2B  LINC00511  AC012645.1  HOXA1  MRPS22  COX5A  TOGARAM2  ROMO1  TCTN3  MFN2  PARPBP  GPI  RPS8  ZNF841  TTC30B  MOSPD1  UBB  WISP2  RSBN1  RRNAD1  CCDC155  CHCHD5  AC073529.1  ALDH3A1  MRPL12  CCDC28A  BCL10  ETFDH  RAD54L  NOTCH3  SPRN  CEBPZOS  WASF1  PDE8B  AC015922.3  LINC02035  PIGW  SAMD9  IP6K1  MADD  NOXA1  ILKAP  AFF4  PEAK1  COL18A1  ZNF733P  ABCB8  TEX9  C5orf24  STYX  FADS3  UCKL1  MSANTD4  RGS2  NFE2L3  DUSP8  MAPRE2  NUFIP2  GALNT14  KMT5A  TRAPPC6A  HNRNPU  CLU  ZC3H18  GPS1  CHID1  IPO9  GEMIN5  COPRS  GPRC5D-AS1  PPAT  MTPN  PARS2  LPGAT1  RBM15B  BNIPL  HEXA  CEBPD  MUS81  DPY19L2P2  MBOAT1  RAB15  ADGRA2  UGT1A6  FAM3A  DNAL1  FIZ1  FBXO21  FNTA  ZNF596  ARPC4  ISOC2  CLASP1  CCDC167  INPP5E  DHX35  RMND1  CHD2  OARD1  ZNF598  MILR1  KIAA0100  SHROOM4  ATR  PPP1R12A  C1RL-AS1  AC092718.7  PPP1R18  EHMT1  ARHGAP27  DDX60  DOCK6  TSEN2  FBXO43  CAMTA2  PANX2  MDH2  GNG2  PIM3  SLC25A15  AC008687.6  XBP1  AP3S2  MSI1  MAPKAP1  AC084337.1  SEC11C  GEMIN8  RPL13A  ARL6IP1  TM4SF1  CCSER2  AL009176.1  ZDHHC1  HHIP  PDGFD  WASHC3  RNF168  MICU1  CDCA7L  CCDC88A  ACAP3  TCEA1  ACD  WASF2  USP25  BUB3  MT1X  TPT1  PPM1F  TMEM216  LINC01963  PIP4P2  LINC00909  FKBP1C  PPP1R12B  EMD  SH3BP5-AS1  OXCT1  PPP1R7  KCNQ3  ATOH8  AKR1C1  TOB2  CTSS  LRRC29  AKAP7  RBPMS2  SSBP2  SREBF1  UTP20  CHEK1  TECR  FRAT2  PRPF40B  OTUD5  OAS1  CENPM  ADI1  C22orf23  FLRT2  AC125437.1  AXIN1  MBNL2  TENT5B  PARP10  PRKAR2A-AS1  AC027020.2  ACP6  MTHFD1  SCAMP1-AS1  HDAC2  PFDN2  PLCL2 | 0  0  0  0  0  3.56E-288  1.15E-270  6.95E-178  9.68E-146  7.34E-141  5.10E-136  3.83E-134  9.12E-127  3.59E-122  2.38E-121  1.63E-120  5.56E-118  5.45E-117  7.89E-116  3.70E-108  3.06E-107  8.66E-105  3.21E-104  7.60E-104  1.72E-103  3.29E-103  2.43E-102  8.15E-101  5.49E-100  1.47E-99  2.38E-93  9.72E-92  7.68E-89  8.62E-89  1.54E-87  6.76E-87  1.04E-81  1.90E-78  9.73E-78  1.82E-77  2.79E-76  6.79E-73  2.20E-72  2.19E-71  2.40E-71  6.62E-71  9.61E-71  1.64E-70  1.65E-70  2.86E-69  4.69E-69  1.17E-67  1.35E-67  2.10E-66  5.59E-66  9.04E-66  9.99E-66  7.71E-64  2.36E-63  7.15E-63  2.29E-62  9.15E-62  1.02E-61  2.59E-61  1.36E-60  1.40E-60  6.34E-60  4.81E-59  2.02E-58  3.78E-58  9.94E-58  1.07E-57  1.18E-57  1.53E-57  1.60E-57  1.88E-57  3.25E-57  3.53E-57  8.48E-57  2.45E-56  1.46E-55  1.79E-55  2.32E-55  5.82E-55  1.97E-54  2.79E-54  4.31E-54  7.97E-54  1.33E-53  3.79E-53  1.05E-52  3.71E-52  3.00E-51  9.20E-51  1.09E-50  1.17E-50  1.57E-50  5.16E-50  3.47E-49  4.39E-49  5.07E-49  5.34E-49  1.63E-48  3.48E-48  4.57E-48  5.11E-48  1.66E-47  2.09E-47  2.85E-47  2.62E-46  4.00E-46  4.14E-46  1.81E-45  2.50E-44  4.99E-44  5.26E-44  8.61E-44  9.87E-44  1.54E-43  2.06E-43  7.16E-43  7.58E-43  1.11E-42  2.16E-42  2.29E-42  4.42E-42  2.73E-41  3.56E-41  3.57E-41  4.90E-41  4.90E-41  6.58E-41  1.05E-40  3.47E-40  6.90E-40  7.64E-40  1.30E-39  1.47E-39  1.60E-39  2.49E-39  2.72E-39  4.69E-39  6.04E-39  6.73E-39  8.55E-39  5.21E-38  9.70E-38  2.45E-37  2.57E-37  2.78E-37  2.78E-37  3.37E-37  7.83E-37  8.73E-37  1.03E-36  1.33E-36  1.80E-36  2.27E-36  5.84E-36  7.11E-36  1.25E-35  1.37E-35  1.81E-35  3.20E-35  3.51E-35  3.92E-35  3.94E-35  3.96E-35  9.44E-35  1.64E-34  2.03E-34  3.51E-34  4.52E-34  5.52E-34  5.59E-34  5.71E-34  5.74E-34  7.33E-34  1.17E-33  1.17E-33  1.17E-33  1.20E-33  2.57E-33  2.86E-33  4.91E-33  4.99E-33  2.44E-32  2.98E-32  3.44E-32  3.75E-32  3.89E-32  5.75E-32  7.46E-32  1.32E-31  1.46E-31  3.45E-31  4.20E-31  4.55E-31  4.68E-31  8.09E-31  8.09E-31  1.03E-30  1.12E-30  1.36E-30  1.97E-30  1.98E-30  2.39E-30  4.05E-30  4.97E-30  6.27E-30  9.99E-30  1.38E-29  2.00E-29  2.43E-29  2.97E-29  4.22E-29  4.82E-29  6.08E-29  6.16E-29  6.22E-29  6.22E-29  7.11E-29  7.57E-29  1.05E-28  1.53E-28  1.72E-28  2.33E-28  2.53E-28  2.75E-28  2.93E-28  4.07E-28  4.87E-28  5.35E-28  6.66E-28  6.67E-28  8.62E-28  9.95E-28  1.06E-27  1.13E-27  1.31E-27  1.43E-27  1.62E-27  1.78E-27  3.70E-27  3.83E-27  4.65E-27  4.71E-27  5.57E-27  5.89E-27  6.18E-27  6.61E-27  7.50E-27  7.84E-27  7.90E-27  8.04E-27  8.74E-27  1.03E-26  1.04E-26  1.13E-26  1.42E-26  1.53E-26  1.61E-26  1.62E-26  1.88E-26  2.04E-26  2.09E-26  2.21E-26  2.59E-26  2.71E-26  4.00E-26  4.33E-26  4.77E-26  5.34E-26  5.45E-26  5.77E-26  6.22E-26  6.22E-26  7.17E-26  8.81E-26  9.18E-26  1.04E-25  1.21E-25  1.55E-25  2.34E-25  3.24E-25  3.64E-25  4.65E-25  5.26E-25  5.66E-25  5.80E-25  5.83E-25  6.07E-25  6.45E-25  7.10E-25  7.44E-25  8.21E-25  8.91E-25  1.04E-24  1.05E-24  1.10E-24  1.53E-24  2.00E-24  2.51E-24  3.27E-24  3.64E-24  3.69E-24  4.80E-24  5.24E-24  6.58E-24  6.58E-24  7.34E-24  7.50E-24  1.04E-23  1.23E-23  1.28E-23  1.60E-23  1.91E-23  2.06E-23  2.07E-23  2.07E-23  2.10E-23  2.24E-23  2.77E-23  3.31E-23  6.42E-23  8.73E-23  1.12E-22  1.30E-22  1.31E-22  1.96E-22  2.29E-22  2.30E-22  2.48E-22  2.80E-22  2.88E-22  3.04E-22  5.06E-22  5.14E-22  6.18E-22  7.24E-22  7.41E-22  8.38E-22  8.40E-22  8.52E-22  8.78E-22  8.81E-22  1.10E-21  1.14E-21  1.18E-21  1.27E-21  1.34E-21  1.40E-21  1.62E-21  1.70E-21  2.15E-21  2.15E-21  2.73E-21  2.98E-21  3.04E-21  3.11E-21  3.13E-21  3.19E-21  3.94E-21  5.02E-21  5.27E-21  5.80E-21  6.30E-21  7.98E-21  8.65E-21  8.92E-21  9.06E-21  9.92E-21  1.08E-20  1.15E-20  1.19E-20  1.36E-20  1.44E-20  1.66E-20  2.15E-20  2.69E-20  2.93E-20  3.02E-20  4.11E-20  4.91E-20  6.63E-20  7.24E-20  7.94E-20  8.01E-20  9.08E-20  9.47E-20  1.12E-19  1.33E-19  1.46E-19  1.87E-19  2.06E-19  2.47E-19  2.65E-19  2.72E-19  3.11E-19  3.16E-19  3.25E-19  3.27E-19  3.76E-19  3.83E-19  4.35E-19  4.41E-19  5.75E-19  5.95E-19  6.42E-19  6.43E-19  6.57E-19  7.79E-19  7.92E-19  9.16E-19  9.67E-19  1.13E-18  1.23E-18  1.27E-18  1.29E-18  1.37E-18  1.41E-18  1.45E-18  1.45E-18  1.51E-18  1.68E-18  1.93E-18  2.15E-18  2.23E-18  2.71E-18  2.95E-18  3.02E-18  3.06E-18  3.29E-18  3.63E-18  3.66E-18  4.45E-18  4.45E-18  4.55E-18  4.65E-18  4.96E-18  5.44E-18  6.26E-18  7.47E-18  8.09E-18  1.03E-17  1.09E-17  1.22E-17  1.35E-17  1.44E-17  1.70E-17  1.73E-17  1.75E-17  1.76E-17  1.99E-17  2.11E-17  2.15E-17  2.17E-17  2.17E-17  2.22E-17  2.24E-17  2.28E-17  2.35E-17  2.53E-17  3.00E-17  3.35E-17  3.41E-17  3.51E-17  4.06E-17  4.15E-17  5.05E-17  5.06E-17  5.31E-17  5.82E-17  6.06E-17  6.06E-17  6.07E-17  6.10E-17  6.13E-17  6.20E-17  6.69E-17  6.70E-17  7.80E-17  9.18E-17  9.25E-17  9.60E-17  1.01E-16  1.08E-16  1.13E-16  1.14E-16  1.16E-16  1.33E-16  1.41E-16  1.45E-16  1.55E-16  1.60E-16  1.62E-16  1.71E-16  1.80E-16  2.05E-16  2.28E-16  2.32E-16  2.46E-16  2.61E-16  2.62E-16  2.86E-16  3.19E-16  3.30E-16  3.52E-16  3.61E-16  3.75E-16  3.80E-16  4.01E-16  4.36E-16  5.01E-16  5.14E-16  5.60E-16  5.93E-16  6.29E-16  6.30E-16  6.43E-16  7.02E-16  7.19E-16  7.53E-16  7.97E-16  8.10E-16  9.29E-16  9.87E-16  1.02E-15  1.04E-15  1.10E-15  1.13E-15  1.28E-15  1.30E-15  1.36E-15  1.40E-15  1.42E-15  1.52E-15  1.57E-15  1.67E-15  1.69E-15  1.71E-15  1.76E-15  1.79E-15  1.84E-15  1.84E-15  2.00E-15  2.27E-15  2.32E-15  2.87E-15  3.04E-15  3.04E-15  3.07E-15  3.18E-15  3.34E-15  3.38E-15  3.55E-15  3.97E-15  4.34E-15  4.54E-15  4.57E-15  4.60E-15  4.93E-15  5.58E-15  5.99E-15  5.99E-15  6.78E-15  6.91E-15  7.07E-15  7.24E-15  8.05E-15  8.05E-15  8.46E-15  8.47E-15  8.76E-15  9.20E-15  9.64E-15  1.03E-14  1.03E-14  1.08E-14  1.18E-14  1.19E-14  1.24E-14  1.24E-14  1.32E-14  1.40E-14  1.40E-14  1.45E-14  1.46E-14  1.50E-14  1.58E-14  1.96E-14  2.02E-14  2.09E-14  2.11E-14  2.11E-14  2.13E-14  2.13E-14  2.34E-14  2.36E-14  2.85E-14  2.88E-14  2.91E-14  2.97E-14  3.02E-14  3.19E-14  3.22E-14  3.24E-14  3.24E-14  3.54E-14  3.67E-14  3.82E-14  3.91E-14  4.11E-14  4.20E-14  4.24E-14  4.52E-14  4.63E-14  4.67E-14  4.83E-14  4.97E-14  5.22E-14  5.23E-14  5.29E-14  5.51E-14  5.51E-14  5.65E-14  5.68E-14  5.69E-14  5.92E-14  5.94E-14  6.14E-14  6.25E-14  6.79E-14  6.88E-14  6.88E-14  7.94E-14  7.96E-14  8.02E-14  8.10E-14  8.48E-14  8.57E-14  8.68E-14  9.84E-14  9.96E-14  1.09E-13  1.09E-13  1.10E-13  1.11E-13  1.14E-13  1.18E-13  1.19E-13  1.21E-13  1.22E-13  1.23E-13  1.24E-13  1.27E-13  1.30E-13  1.49E-13  1.51E-13  1.51E-13  1.54E-13  1.68E-13  1.79E-13  1.87E-13  1.99E-13  2.05E-13  2.17E-13  2.19E-13  2.22E-13  2.36E-13  2.49E-13  2.53E-13  2.63E-13  2.69E-13  3.00E-13  3.36E-13  3.49E-13  3.97E-13  4.21E-13  4.55E-13  4.58E-13  4.62E-13  4.64E-13  4.69E-13  4.98E-13  5.05E-13  5.08E-13  5.23E-13  5.32E-13  5.65E-13  5.67E-13  5.67E-13  5.88E-13  6.02E-13  6.07E-13  6.07E-13  6.15E-13  6.25E-13  6.33E-13  6.41E-13  6.77E-13  6.77E-13  6.77E-13  6.81E-13  6.85E-13  6.88E-13  6.98E-13  6.98E-13  7.00E-13  7.01E-13  7.25E-13  7.33E-13  7.38E-13  7.48E-13  7.48E-13  7.48E-13  7.67E-13  8.88E-13  8.89E-13  9.09E-13  9.36E-13  9.54E-13  9.66E-13  9.82E-13  9.82E-13  1.00E-12  1.03E-12  1.07E-12  1.15E-12  1.15E-12  1.18E-12  1.21E-12  1.22E-12  1.24E-12  1.30E-12  1.31E-12  1.38E-12  1.41E-12  1.45E-12  1.47E-12  1.58E-12  1.60E-12  1.66E-12  1.73E-12  1.78E-12  1.79E-12  1.80E-12  1.91E-12  2.07E-12  2.21E-12  2.25E-12  2.27E-12  2.39E-12  2.51E-12  2.56E-12  2.69E-12  2.70E-12  2.91E-12  2.99E-12  3.24E-12  3.39E-12  3.41E-12  3.49E-12  3.52E-12  3.64E-12  3.69E-12  3.76E-12  3.76E-12  3.97E-12  4.08E-12  4.12E-12  4.23E-12  4.81E-12  5.05E-12  5.13E-12  5.19E-12  5.49E-12  5.50E-12  5.62E-12  5.74E-12  5.89E-12  6.28E-12  6.33E-12  6.42E-12  7.56E-12  7.68E-12  7.93E-12  8.01E-12  8.02E-12  8.29E-12  8.33E-12  8.35E-12  8.55E-12  9.12E-12  9.16E-12  9.16E-12  9.16E-12  1.01E-11  1.04E-11  1.09E-11  1.10E-11  1.10E-11  1.11E-11  1.13E-11  1.14E-11  1.18E-11  1.21E-11  1.23E-11  1.23E-11  1.30E-11  1.30E-11  1.31E-11  1.34E-11  1.35E-11  1.37E-11  1.40E-11  1.41E-11  1.42E-11  1.44E-11  1.45E-11  1.46E-11  1.48E-11  1.53E-11  1.54E-11  1.55E-11  1.66E-11  1.77E-11  1.77E-11  1.85E-11  1.85E-11  1.88E-11  1.94E-11  2.07E-11  2.07E-11  2.13E-11  2.16E-11  2.16E-11  2.18E-11  2.19E-11  2.30E-11  2.32E-11  2.33E-11  2.47E-11  2.66E-11  2.70E-11  2.74E-11  2.82E-11  2.93E-11  2.93E-11  3.00E-11  3.07E-11  3.08E-11  3.08E-11  3.08E-11  3.12E-11  3.15E-11  3.17E-11  3.25E-11  3.39E-11  3.78E-11  3.79E-11  3.81E-11  3.97E-11  4.06E-11  4.17E-11  4.39E-11  4.60E-11  4.60E-11  4.63E-11  4.86E-11  4.87E-11  4.96E-11  5.07E-11  5.13E-11  5.21E-11  5.51E-11  5.84E-11  5.99E-11  6.20E-11  6.28E-11  6.39E-11  6.41E-11  6.78E-11  6.78E-11  6.92E-11  6.95E-11  6.95E-11  7.66E-11  7.74E-11  7.88E-11  8.07E-11  8.15E-11  8.15E-11  8.64E-11  8.67E-11  8.85E-11  8.88E-11  9.32E-11  9.48E-11  1.02E-10  1.03E-10  1.04E-10  1.07E-10  1.07E-10  1.07E-10  1.07E-10  1.10E-10  1.11E-10  1.12E-10  1.16E-10  1.18E-10  1.21E-10  1.21E-10  1.21E-10  1.26E-10  1.27E-10  1.27E-10  1.30E-10  1.32E-10  1.34E-10  1.37E-10  1.39E-10  1.47E-10  1.51E-10  1.65E-10  1.66E-10  1.67E-10  1.74E-10  1.77E-10  1.81E-10  1.82E-10  1.83E-10  1.88E-10  2.03E-10  2.11E-10  2.11E-10  2.32E-10  2.32E-10  2.33E-10  2.34E-10  2.48E-10  2.49E-10  2.52E-10  2.57E-10  2.57E-10  2.64E-10  2.66E-10  2.66E-10  2.87E-10  2.92E-10  2.98E-10  2.99E-10  3.00E-10  3.11E-10  3.18E-10  3.20E-10  3.40E-10  3.47E-10  3.60E-10  3.71E-10  3.72E-10  3.79E-10  3.91E-10  3.92E-10  4.03E-10  4.08E-10  4.25E-10  4.28E-10  4.29E-10  4.31E-10  4.38E-10  4.49E-10  4.65E-10  4.75E-10  4.88E-10  4.91E-10  4.91E-10  5.15E-10  5.15E-10  5.21E-10  5.34E-10  5.37E-10  5.37E-10  5.50E-10  5.67E-10  5.96E-10  6.22E-10  6.32E-10  6.37E-10  6.43E-10  6.77E-10  6.79E-10  6.89E-10  6.89E-10  6.99E-10  7.05E-10  7.05E-10  7.59E-10  7.67E-10  7.90E-10  7.95E-10  8.01E-10  8.58E-10  8.64E-10  8.70E-10  8.75E-10  8.84E-10  9.18E-10  9.20E-10  9.52E-10  9.52E-10  9.83E-10  9.95E-10  1.00E-09  1.02E-09  1.05E-09  1.05E-09  1.05E-09  1.06E-09  1.07E-09  1.08E-09  1.09E-09  1.09E-09  1.09E-09  1.11E-09  1.12E-09  1.12E-09  1.13E-09  1.14E-09  1.15E-09  1.17E-09  1.17E-09  1.17E-09  1.17E-09  1.20E-09  1.24E-09  1.28E-09  1.28E-09  1.29E-09  1.33E-09  1.36E-09  1.36E-09  1.36E-09  1.38E-09  1.41E-09  1.41E-09  1.42E-09  1.46E-09  1.46E-09  1.54E-09  1.58E-09  1.63E-09  1.78E-09  1.81E-09  1.83E-09  1.85E-09  1.98E-09  1.99E-09  2.05E-09  2.17E-09  2.22E-09  2.22E-09  2.24E-09  2.27E-09  2.31E-09  2.33E-09  2.34E-09  2.45E-09  2.47E-09  2.54E-09  2.58E-09  2.62E-09  2.64E-09  2.65E-09  2.78E-09  2.78E-09  2.82E-09  2.83E-09  2.83E-09  2.84E-09  2.91E-09  3.07E-09  3.15E-09  3.24E-09  3.30E-09  3.33E-09  3.34E-09  3.34E-09  3.39E-09  3.41E-09  3.42E-09  3.56E-09  3.58E-09  3.60E-09  3.60E-09  3.60E-09  3.62E-09  3.70E-09  3.72E-09  3.74E-09  3.74E-09  3.75E-09  3.78E-09  3.85E-09  3.94E-09  3.96E-09  3.96E-09  3.98E-09  3.98E-09  3.98E-09  4.04E-09  4.11E-09  4.12E-09  4.33E-09  4.35E-09  4.35E-09  4.36E-09  4.36E-09  4.36E-09  4.41E-09  4.48E-09  4.52E-09  4.59E-09  4.72E-09  4.90E-09  5.10E-09  5.14E-09  5.21E-09  5.23E-09  5.32E-09  5.40E-09  5.43E-09  5.65E-09  5.67E-09  5.69E-09  5.70E-09  5.76E-09  5.84E-09  5.92E-09  6.16E-09  6.18E-09  6.18E-09  6.31E-09  6.41E-09  6.42E-09  6.42E-09  6.43E-09  6.48E-09  6.55E-09  6.66E-09  6.66E-09  6.97E-09  7.14E-09  7.16E-09  7.16E-09  7.16E-09  7.33E-09  7.39E-09  7.65E-09  7.67E-09  7.75E-09  7.90E-09  7.96E-09  8.15E-09  8.20E-09  8.31E-09  8.46E-09  8.66E-09  8.87E-09  9.05E-09  9.37E-09  9.37E-09  9.47E-09  9.56E-09  9.62E-09  9.75E-09  9.83E-09  9.83E-09  1.00E-08  1.02E-08  1.04E-08  1.05E-08  1.06E-08  1.06E-08  1.07E-08  1.10E-08  1.15E-08  1.16E-08  1.16E-08  1.17E-08  1.18E-08  1.20E-08  1.22E-08  1.25E-08  1.25E-08  1.25E-08  1.26E-08  1.26E-08  1.28E-08  1.33E-08  1.38E-08  1.41E-08  1.49E-08  1.55E-08  1.56E-08  1.57E-08  1.58E-08  1.62E-08  1.62E-08  1.62E-08  1.62E-08  1.63E-08  1.64E-08  1.65E-08  1.65E-08  1.66E-08  1.69E-08  1.77E-08  1.85E-08  1.85E-08  1.86E-08  1.87E-08  1.94E-08  1.97E-08  1.99E-08  2.01E-08  2.12E-08  2.12E-08  2.12E-08  2.17E-08  2.24E-08  2.33E-08  2.37E-08  2.37E-08  2.41E-08  2.42E-08  2.42E-08  2.47E-08  2.57E-08  2.67E-08  2.70E-08  2.74E-08  2.75E-08  2.75E-08  2.77E-08  2.77E-08  2.91E-08  2.94E-08  3.08E-08  3.25E-08  3.25E-08  3.28E-08  3.36E-08  3.38E-08  3.41E-08  3.41E-08  3.45E-08  3.45E-08  3.49E-08  3.49E-08  3.49E-08  3.74E-08  3.76E-08  3.76E-08  3.81E-08  3.84E-08  3.86E-08  3.88E-08  3.92E-08  4.01E-08  4.09E-08  4.12E-08  4.14E-08  4.23E-08  4.26E-08  4.34E-08  4.36E-08  4.50E-08  4.54E-08  4.60E-08  4.61E-08  4.72E-08  4.87E-08  4.99E-08  5.00E-08  5.00E-08  5.12E-08  5.15E-08  5.17E-08  5.25E-08  5.36E-08  5.43E-08  5.51E-08  5.65E-08  5.71E-08  5.75E-08  5.76E-08  5.93E-08  5.96E-08  6.08E-08  6.29E-08  6.45E-08  6.55E-08  6.56E-08  6.69E-08  6.69E-08  6.76E-08  6.76E-08  6.76E-08  6.78E-08  6.79E-08  6.86E-08  6.86E-08  6.93E-08  7.07E-08  7.08E-08  7.08E-08  7.20E-08  7.27E-08  7.28E-08  7.29E-08  7.33E-08  7.36E-08  7.43E-08  7.53E-08  7.56E-08  7.75E-08  7.75E-08  7.88E-08  7.99E-08  8.08E-08  8.16E-08  8.36E-08  8.79E-08  9.48E-08  9.89E-08  9.91E-08  1.01E-07  1.01E-07  1.04E-07  1.05E-07  1.05E-07  1.05E-07  1.07E-07  1.09E-07  1.09E-07  1.11E-07  1.14E-07  1.16E-07  1.16E-07  1.19E-07  1.19E-07  1.19E-07  1.20E-07  1.21E-07  1.22E-07  1.24E-07  1.28E-07  1.28E-07  1.29E-07  1.30E-07  1.35E-07  1.36E-07  1.38E-07  1.38E-07  1.39E-07  1.39E-07  1.41E-07  1.45E-07  1.47E-07  1.48E-07  1.50E-07  1.50E-07  1.51E-07  1.53E-07  1.61E-07  1.68E-07  1.68E-07  1.70E-07  1.70E-07  1.72E-07  1.76E-07  1.79E-07  1.81E-07  1.81E-07  1.88E-07  1.92E-07  1.97E-07  1.98E-07  1.98E-07  2.01E-07  2.05E-07  2.08E-07  2.08E-07  2.09E-07  2.10E-07  2.11E-07  2.15E-07  2.17E-07  2.17E-07  2.18E-07  2.21E-07  2.27E-07  2.27E-07  2.28E-07  2.29E-07  2.30E-07  2.48E-07  2.48E-07  2.53E-07  2.57E-07  2.63E-07  2.76E-07  2.77E-07  2.81E-07  2.87E-07  2.93E-07  2.95E-07  3.06E-07  3.07E-07  3.08E-07  3.16E-07  3.17E-07  3.19E-07  3.20E-07  3.23E-07  3.25E-07  3.31E-07  3.33E-07  3.35E-07  3.42E-07  3.55E-07  3.65E-07  3.79E-07  3.81E-07  3.81E-07  3.84E-07  3.89E-07  3.92E-07  3.99E-07  4.03E-07  4.13E-07  4.14E-07  4.23E-07  4.28E-07  4.28E-07  4.32E-07  4.33E-07  4.33E-07  4.37E-07  4.37E-07  4.54E-07  4.55E-07  4.63E-07  4.64E-07  4.73E-07  4.75E-07  4.87E-07  4.88E-07  4.93E-07  4.95E-07  5.02E-07  5.13E-07  5.13E-07  5.13E-07  5.23E-07  5.27E-07  5.28E-07  5.53E-07  5.64E-07  5.67E-07  5.69E-07  5.73E-07  5.82E-07  5.98E-07  5.99E-07  6.01E-07  6.01E-07  6.04E-07  6.14E-07  6.16E-07  6.52E-07  6.52E-07  6.56E-07  6.59E-07  6.84E-07  6.85E-07  6.91E-07  6.95E-07  7.06E-07  7.08E-07  7.09E-07  7.12E-07  7.12E-07  7.16E-07  7.16E-07  7.26E-07  7.31E-07  7.31E-07  7.31E-07  7.32E-07  7.35E-07  7.43E-07  7.46E-07  7.48E-07  7.63E-07  7.72E-07  7.72E-07  7.80E-07  7.82E-07  7.92E-07  7.92E-07  7.95E-07  8.23E-07  8.45E-07  8.45E-07  8.52E-07  8.58E-07  8.81E-07  8.83E-07  8.88E-07  8.90E-07  8.93E-07  9.00E-07  9.03E-07  9.04E-07  9.17E-07  9.33E-07  9.38E-07  9.46E-07  9.56E-07  9.69E-07  9.72E-07  9.72E-07  9.95E-07  1.00E-06  1.02E-06  1.05E-06  1.06E-06  1.08E-06  1.08E-06  1.09E-06  1.09E-06  1.10E-06  1.10E-06  1.11E-06  1.12E-06  1.12E-06  1.12E-06  1.12E-06  1.13E-06  1.13E-06  1.13E-06  1.14E-06  1.15E-06  1.17E-06  1.21E-06  1.21E-06  1.22E-06  1.22E-06  1.25E-06  1.25E-06  1.26E-06  1.27E-06  1.27E-06  1.28E-06  1.32E-06  1.32E-06  1.37E-06  1.40E-06  1.40E-06  1.41E-06  1.41E-06  1.42E-06  1.43E-06  1.44E-06  1.44E-06  1.45E-06  1.47E-06  1.50E-06  1.51E-06  1.55E-06  1.56E-06  1.57E-06  1.58E-06  1.61E-06  1.71E-06  1.71E-06  1.73E-06  1.75E-06  1.75E-06  1.76E-06  1.77E-06  1.78E-06  1.79E-06  1.79E-06  1.79E-06  1.79E-06  1.82E-06  1.85E-06  1.86E-06  1.86E-06  1.86E-06  1.89E-06  1.91E-06  1.93E-06  2.00E-06  2.00E-06  2.02E-06  2.04E-06  2.09E-06  2.09E-06  2.10E-06  2.13E-06  2.14E-06  2.16E-06  2.18E-06  2.18E-06  2.20E-06  2.23E-06  2.25E-06  2.25E-06  2.26E-06  2.29E-06  2.31E-06  2.31E-06  2.32E-06  2.35E-06  2.37E-06  2.38E-06  2.40E-06  2.40E-06  2.41E-06  2.44E-06  2.46E-06  2.46E-06  2.48E-06  2.49E-06  2.49E-06  2.50E-06  2.53E-06  2.54E-06  2.54E-06  2.55E-06  2.57E-06  2.57E-06  2.61E-06  2.61E-06  2.70E-06  2.74E-06  2.74E-06  2.76E-06  2.76E-06  2.76E-06  2.79E-06  2.84E-06  2.85E-06  2.88E-06  2.92E-06  2.92E-06  2.99E-06  3.05E-06  3.06E-06  3.09E-06  3.09E-06  3.12E-06  3.16E-06  3.16E-06  3.19E-06  3.20E-06  3.20E-06  3.20E-06  3.21E-06  3.22E-06  3.24E-06  3.31E-06  3.32E-06  3.32E-06  3.32E-06  3.35E-06  3.36E-06  3.37E-06  3.38E-06  3.39E-06  3.41E-06  3.42E-06  3.43E-06  3.50E-06  3.53E-06  3.59E-06  3.59E-06  3.60E-06  3.65E-06  3.65E-06  3.65E-06  3.66E-06  3.74E-06  3.74E-06  3.82E-06  3.83E-06  3.86E-06  3.86E-06  3.88E-06  3.88E-06  3.93E-06  3.94E-06  3.94E-06  3.97E-06  3.98E-06  4.04E-06  4.04E-06  4.08E-06  4.10E-06  4.10E-06  4.18E-06  4.19E-06  4.22E-06  4.24E-06  4.35E-06  4.37E-06  4.42E-06  4.42E-06  4.43E-06  4.48E-06  4.62E-06  4.62E-06  4.69E-06  4.75E-06  4.79E-06  4.83E-06  4.91E-06  4.91E-06  4.93E-06  4.95E-06  4.96E-06  4.98E-06  4.98E-06  4.99E-06  5.00E-06  5.01E-06  5.01E-06  5.03E-06  5.04E-06  5.07E-06  5.11E-06  5.12E-06  5.16E-06  5.18E-06  5.28E-06  5.28E-06  5.28E-06  5.30E-06  5.38E-06  5.39E-06  5.45E-06  5.45E-06  5.48E-06  5.48E-06  5.56E-06  5.57E-06  5.66E-06  5.72E-06  5.75E-06  5.77E-06  5.77E-06  5.77E-06  5.85E-06  5.94E-06  5.94E-06  5.96E-06  5.97E-06  6.01E-06  6.04E-06  6.06E-06  6.14E-06  6.21E-06  6.22E-06  6.23E-06  6.25E-06  6.38E-06  6.43E-06  6.50E-06  6.51E-06  6.54E-06  6.59E-06  6.59E-06  6.63E-06  6.68E-06  6.72E-06  6.73E-06  6.77E-06  6.77E-06  6.78E-06  6.89E-06  6.90E-06  6.94E-06  6.95E-06  7.02E-06  7.02E-06  7.08E-06  7.10E-06  7.12E-06  7.17E-06  7.30E-06  7.42E-06  7.42E-06  7.44E-06  7.54E-06  7.55E-06  7.67E-06  7.72E-06  7.83E-06  7.89E-06  7.94E-06  7.95E-06  7.98E-06  8.04E-06  8.22E-06  8.29E-06  8.70E-06  8.98E-06  9.26E-06  9.27E-06  9.27E-06  9.27E-06  9.28E-06  9.28E-06  9.29E-06  9.29E-06  9.48E-06  9.57E-06  9.57E-06  9.58E-06  9.59E-06  9.64E-06  9.70E-06  9.75E-06  9.86E-06  9.93E-06  9.94E-06  9.94E-06  9.98E-06  9.99E-06  1.00E-05  1.01E-05  1.02E-05  1.02E-05  1.02E-05  1.03E-05  1.04E-05  1.04E-05  1.04E-05  1.04E-05  1.05E-05  1.05E-05  1.05E-05  1.05E-05  1.06E-05  1.06E-05  1.07E-05  1.09E-05  1.09E-05  1.09E-05  1.11E-05  1.11E-05  1.11E-05  1.11E-05  1.11E-05  1.13E-05  1.13E-05  1.13E-05  1.13E-05  1.13E-05  1.13E-05  1.14E-05  1.16E-05  1.16E-05  1.16E-05  1.16E-05  1.17E-05  1.17E-05  1.18E-05  1.18E-05  1.19E-05  1.20E-05  1.21E-05  1.21E-05  1.22E-05  1.23E-05  1.24E-05  1.24E-05  1.26E-05  1.26E-05  1.26E-05  1.27E-05  1.27E-05  1.28E-05  1.29E-05  1.29E-05  1.30E-05  1.32E-05  1.32E-05  1.33E-05  1.34E-05  1.35E-05  1.36E-05  1.36E-05  1.37E-05  1.37E-05  1.38E-05  1.38E-05  1.39E-05  1.39E-05  1.39E-05  1.42E-05  1.42E-05  1.47E-05  1.47E-05  1.47E-05  1.47E-05  1.48E-05  1.48E-05  1.48E-05  1.48E-05  1.49E-05  1.49E-05  1.51E-05  1.51E-05  1.51E-05  1.52E-05  1.52E-05  1.54E-05  1.54E-05  1.55E-05  1.57E-05  1.57E-05  1.57E-05  1.58E-05  1.58E-05  1.59E-05  1.61E-05  1.65E-05  1.65E-05  1.66E-05  1.66E-05  1.66E-05  1.67E-05  1.67E-05  1.67E-05  1.67E-05  1.69E-05  1.70E-05  1.70E-05  1.71E-05  1.75E-05  1.77E-05  1.77E-05  1.79E-05  1.80E-05  1.80E-05  1.80E-05  1.80E-05  1.84E-05  1.85E-05  1.85E-05  1.85E-05  1.86E-05  1.86E-05  1.86E-05  1.86E-05  1.89E-05  1.92E-05  1.97E-05  2.00E-05  2.02E-05  2.03E-05  2.07E-05  2.09E-05  2.09E-05  2.10E-05  2.10E-05  2.10E-05  2.10E-05  2.10E-05  2.11E-05  2.12E-05  2.13E-05  2.16E-05  2.16E-05  2.19E-05  2.19E-05  2.21E-05  2.21E-05  2.21E-05  2.24E-05  2.28E-05  2.29E-05  2.29E-05  2.30E-05  2.30E-05  2.33E-05  2.33E-05  2.34E-05  2.34E-05  2.36E-05  2.37E-05  2.40E-05  2.42E-05  2.43E-05  2.46E-05  2.46E-05  2.47E-05  2.47E-05  2.48E-05  2.49E-05  2.49E-05  2.50E-05  2.50E-05  2.51E-05  2.53E-05  2.53E-05  2.55E-05  2.55E-05  2.57E-05  2.57E-05  2.57E-05  2.58E-05  2.58E-05  2.61E-05  2.61E-05  2.62E-05  2.63E-05  2.66E-05  2.68E-05  2.69E-05  2.69E-05  2.69E-05  2.75E-05  2.77E-05  2.79E-05  2.80E-05  2.82E-05  2.82E-05  2.83E-05  2.84E-05  2.85E-05  2.86E-05  2.86E-05  2.88E-05  2.93E-05  2.95E-05  2.95E-05  2.96E-05  2.98E-05  2.99E-05  3.00E-05  3.01E-05  3.05E-05  3.05E-05  3.07E-05  3.12E-05  3.13E-05  3.15E-05  3.15E-05  3.18E-05  3.18E-05  3.20E-05  3.20E-05  3.21E-05  3.22E-05  3.22E-05  3.25E-05  3.26E-05  3.27E-05  3.28E-05  3.29E-05  3.29E-05  3.31E-05  3.33E-05  3.39E-05  3.39E-05  3.41E-05  3.42E-05  3.42E-05  3.45E-05  3.47E-05  3.47E-05  3.47E-05  3.47E-05  3.48E-05  3.49E-05  3.54E-05  3.55E-05  3.60E-05  3.65E-05  3.69E-05  3.69E-05  3.70E-05  3.70E-05  3.72E-05  3.77E-05  3.80E-05  3.80E-05  3.85E-05  3.86E-05  3.86E-05  3.87E-05  3.94E-05  4.00E-05  4.00E-05  4.03E-05  4.04E-05  4.08E-05  4.08E-05  4.08E-05  4.09E-05  4.12E-05  4.15E-05  4.17E-05  4.18E-05  4.21E-05  4.23E-05  4.29E-05  4.29E-05  4.29E-05  4.30E-05  4.32E-05  4.33E-05  4.36E-05  4.36E-05  4.36E-05  4.40E-05  4.40E-05  4.41E-05  4.44E-05  4.49E-05  4.50E-05  4.51E-05  4.53E-05  4.56E-05  4.60E-05  4.61E-05  4.62E-05  4.71E-05  4.77E-05  4.81E-05  4.83E-05  4.98E-05  4.99E-05  5.02E-05  5.05E-05  5.08E-05  5.08E-05  5.17E-05  5.19E-05  5.21E-05  5.25E-05  5.28E-05  5.29E-05  5.34E-05  5.40E-05  5.42E-05  5.46E-05  5.46E-05  5.48E-05  5.54E-05  5.55E-05  5.55E-05  5.56E-05  5.59E-05  5.60E-05  5.69E-05  5.74E-05  5.74E-05  5.77E-05  5.83E-05  5.86E-05  5.88E-05  5.88E-05  5.89E-05  6.00E-05  6.01E-05  6.01E-05  6.02E-05  6.05E-05  6.07E-05  6.10E-05  6.10E-05  6.13E-05  6.13E-05  6.17E-05  6.17E-05  6.19E-05  6.29E-05  6.32E-05  6.36E-05  6.38E-05  6.40E-05  6.43E-05  6.45E-05  6.45E-05  6.46E-05  6.49E-05  6.52E-05  6.56E-05  6.58E-05  6.59E-05  6.72E-05  6.75E-05  6.82E-05  6.83E-05  6.88E-05  6.92E-05  6.92E-05  6.94E-05  6.96E-05  7.00E-05  7.01E-05  7.01E-05  7.02E-05  7.07E-05  7.08E-05  7.16E-05  7.19E-05  7.21E-05  7.21E-05  7.24E-05  7.26E-05  7.31E-05  7.33E-05  7.33E-05  7.34E-05  7.36E-05  7.36E-05  7.38E-05  7.43E-05  7.51E-05  7.51E-05  7.54E-05  7.57E-05  7.66E-05  7.71E-05  7.73E-05  7.77E-05  7.83E-05  7.88E-05  7.90E-05  7.97E-05  8.04E-05  8.04E-05  8.06E-05  8.31E-05  8.43E-05  8.44E-05  8.45E-05  8.45E-05  8.45E-05  8.47E-05  8.58E-05  8.65E-05  8.66E-05  8.70E-05  8.71E-05  8.76E-05  8.76E-05  8.82E-05  8.96E-05  9.07E-05  9.07E-05  9.10E-05  9.14E-05  9.14E-05  9.16E-05  9.16E-05  9.21E-05  9.23E-05  9.25E-05  9.35E-05  9.38E-05  9.46E-05  9.51E-05  9.64E-05  9.68E-05  9.73E-05  9.79E-05  9.83E-05  9.85E-05  9.87E-05  9.87E-05  0.000100112  0.000102998  0.000104229  0.000104328  0.000104377  0.000104493  0.000104681  0.00010556  0.000106442  0.000106741  0.000106833  0.00010721  0.000107889  0.000108449  0.000109232  0.000109303  0.000109303  0.000110278  0.000110369  0.000111411  0.000111411  0.00011184  0.000112211  0.000112211  0.000112473  0.000112648  0.000114665  0.000114851  0.000115224  0.000115919  0.000116112  0.00011725  0.000117436  0.00011746  0.000117734  0.000117734  0.000119097  0.000119301  0.000119301  0.000119301  0.000119434  0.000120013  0.000120223  0.000121497  0.000121971  0.000122295  0.000122962  0.000125372  0.000125582  0.000126129  0.000126792  0.000127026  0.000128008  0.000130551  0.000131468  0.000133487  0.000133869  0.000134537  0.000137856  0.000137977  0.000138112  0.000138669  0.000138762  0.000139371  0.000140381  0.000140812  0.000141391  0.00014209  0.00014232  0.000142408  0.000142536  0.000143912  0.000144619  0.000144942  0.000146825  0.000146842  0.000147325  0.000148096  0.000149517  0.000149818  0.000149988  0.000150124  0.000151068  0.000152692  0.00015338  0.000153574  0.000154252  0.000155263  0.000155498  0.000155498  0.000156326  0.000156947  0.000156947  0.000157695  0.000157695  0.000159088  0.000160105  0.00016289  0.000163785  0.000164285  0.000165162  0.00016628  0.00016628  0.00016628  0.000167155  0.000167839  0.000169024  0.00016915  0.000170033  0.000170158  0.000170158  0.00017091  0.00017452  0.000175171  0.000175805  0.000177125  0.000178827  0.00017896  0.000179318  0.000179465  0.000179549  0.000180704  0.000180871  0.000181857  0.000182641  0.000182665  0.000182742  0.000183787  0.000183843  0.000185203  0.000186066  0.000186718  0.000187524  0.000187941  0.000188033  0.000188117  0.000188979  0.000189735  0.000189956  0.000190718  0.000195769  0.000196363  0.000196576  0.000197098  0.000198074  0.000198774  0.000198902  0.000201726  0.000201883  0.000203795  0.000204505  0.000204887  0.000205385  0.000207089  0.000207176  0.000207657  0.000207729  0.000207853  0.000208286  0.000208504  0.000209271  0.000210091  0.000210213  0.00021054  0.00021054  0.000210963  0.000212369  0.000213902  0.000214726  0.000214726  0.000217208  0.000217347  0.000218213  0.000219364  0.000220233  0.00022094  0.000223244  0.000223453  0.000225186  0.000225363  0.00022548  0.000225627  0.000226184  0.000226712  0.000226858  0.00022713  0.000227996  0.000227996  0.000228292  0.000229062  0.000229062  0.000229339  0.000230379  0.000230484  0.000231997  0.000231997  0.0002325  0.000232516  0.000233699  0.00023561  0.000236873  0.000236873  0.000238986  0.000239905  0.000241895  0.000242104  0.000242165  0.000242226  0.000242226  0.000243625  0.000244025  0.000245666  0.000247185  0.000248733  0.00024931  0.00025028  0.000251503  0.000251845  0.000253938  0.000255171  0.000258061  0.000259205  0.000260846  0.000262691  0.000263101  0.000263755  0.000264122  0.000265699  0.000267883  0.000267883  0.000269721  0.000270862  0.00027136  0.000273113  0.000273384  0.000273384  0.000273427  0.000274965  0.000275292  0.000275322  0.000275721  0.000275874  0.000277343  0.000282856  0.000284703  0.000285319  0.000288541  0.000290706  0.000291064  0.000292612  0.000292667  0.000294239  0.00029605  0.000296541  0.000298189  0.000299475  0.000301431  0.000308114  0.000309393  0.000309423  0.000310956  0.00031256  0.000313942  0.00031715  0.000317234  0.000317357  0.00031796  0.000319894  0.000319904  0.000320487  0.000321194  0.000324263  0.000325121  0.000325515  0.000325659  0.000325708  0.000327394  0.000327958  0.000329285  0.000329762  0.00033087  0.000331542  0.000332449  0.000333298  0.000335638  0.000338707  0.000339994  0.000339994  0.000339994  0.000341755  0.000343469  0.000346687  0.000347482  0.00034922  0.000350923  0.000352109  0.000352834  0.000353296  0.000355057  0.000355524  0.0003559  0.000359274  0.000360052  0.000360067  0.000360067  0.000360067  0.000363624  0.000363827  0.000364485  0.000364969  0.00036557  0.00036565  0.000365701  0.000365701  0.000367991  0.000368328  0.000371358  0.000372309  0.000374171  0.000374312  0.000376318  0.000377946  0.000378346  0.000379899  0.000382787  0.000384307  0.000384458  0.000386279  0.000386909  0.000386909  0.000386909  0.000386909  0.000387899  0.000391096  0.000391708  0.000392381  0.000392742  0.00039313  0.000397981  0.00039849  0.000401701  0.000402901  0.000403246  0.000405229  0.000405903  0.000406274  0.000408257  0.000411002  0.000412763  0.000413244  0.000413739  0.000418052  0.000419709  0.000422559  0.000426044  0.000426044  0.000427581  0.00042787  0.00043267  0.000434393  0.000434406  0.000434547  0.000434547  0.000436878  0.000437901  0.000439474  0.00044057  0.000440999  0.000441044  0.00044208  0.000442112  0.000442788  0.000442911  0.000444637  0.000447161  0.000447994  0.000449603  0.000450827  0.000451919  0.000454577  0.000454682  0.000454983  0.000454983  0.000455008  0.000456577  0.000456953  0.000459627  0.000459627  0.00045981  0.000460236  0.000462546  0.000463243  0.000463286  0.000463286  0.000463286  0.00046442  0.000465679  0.000466448  0.00046704  0.000471027  0.000475315  0.000476017  0.000477748  0.000477748  0.000478839  0.000480301  0.000480301  0.000482197  0.000483509  0.000485541  0.000485541  0.000485564  0.000485619  0.000485677  0.000486483  0.000487891  0.000488712  0.000495551  0.000496683  0.000501185  0.000503597  0.000505127  0.000507015  0.000508137  0.000508137  0.000508137  0.000514178  0.000517868  0.000519121  0.00052178  0.000524298  0.000524596  0.000525088  0.000525187  0.000527074  0.000528578  0.000528797  0.000532262  0.000532378  0.000533774  0.000539572  0.000540061  0.00054035  0.000542496  0.000542541  0.00054303  0.000546822  0.000547124  0.00054847  0.000548891  0.000550064  0.000552413  0.000553759  0.000554258  0.000556102  0.000556102  0.000561816  0.000565943  0.000567711  0.000568094  0.000568163  0.000569647  0.000569647  0.000571628  0.000575336  0.000575687  0.00057658  0.000576625  0.000577729  0.000578591  0.000580843  0.000581934  0.000592852  0.000593647  0.000599302  0.000599949  0.000601934  0.000603132  0.00060427  0.000610615  0.000617158  0.000617158  0.000617217  0.000618031  0.000618628  0.000620036  0.000620571  0.000624014  0.000626066  0.000627509  0.000629124  0.000629458  0.000630112  0.000630773  0.000630952  0.000630952  0.000631  0.00063338  0.00063371  0.000633962  0.000634551  0.000636219  0.000636361  0.000637428  0.000639778  0.00064455  0.000645487  0.000646903  0.000648616  0.000654869  0.000657764  0.000657764  0.000658411  0.000661786  0.000662791  0.000665337  0.000665467  0.000665467  0.000665502  0.000666765  0.000667246  0.000667246  0.000668954  0.000669129  0.000672011  0.000672143  0.000672363  0.000682748  0.000683546  0.000683795  0.000683795  0.000683795  0.000684624  0.000688978  0.000691276  0.000695399  0.000695399  0.00069661  0.00069661  0.000697846  0.000699461  0.000699843  0.000700447  0.000702594  0.000704239  0.000704239  0.000707746  0.000709735  0.000710142  0.0007169  0.00071712  0.000717852  0.000718755  0.000719908  0.000723775  0.000725903  0.000725903  0.000737149  0.000739877  0.000740886  0.00075001  0.000754649  0.000755645  0.00075709  0.000757812  0.000760337  0.000761804  0.000762386  0.000762835  0.000764706  0.000767356  0.00077049  0.000775307  0.000775391  0.000775391  0.000780208  0.000780208  0.000782155  0.000782617  0.000782802  0.000784004  0.000786536  0.000786536  0.000788696  0.000788696  0.000789453  0.000795552  0.000796267  0.000797487  0.000801567  0.000802549  0.000807468  0.000807666  0.000807666  0.000809457  0.000809774  0.000811173  0.000816747  0.000818713  0.000822575  0.000826254  0.000826424  0.00083388  0.000836091  0.000839157  0.000839157  0.000845532  0.000845532  0.000845554  0.000847297  0.000858319  0.000863387  0.000863769  0.000865268  0.000865268  0.000865268  0.00086621  0.000867068  0.000867068  0.000867905  0.000870333  0.000871204  0.000876069  0.000878619  0.000881413  0.000887845  0.000888639  0.00089074  0.000891031  0.000895008  0.000895008  0.00089951  0.000900559  0.000906404  0.000906404  0.00091048  0.000913508  0.000917731  0.00091963  0.00091963  0.000921449  0.000929071  0.000930978  0.000937475  0.000939908  0.000946934  0.000947172  0.000947508  0.00094841  0.00095201  0.000958332  0.000964251  0.000969601  0.000970173  0.000975967  0.000978716  0.000981257  0.000987765  0.000990776  0.000999006  0.000999515  0.001000446  0.00100215  0.001003821  0.001022378  0.001034525  0.001034668  0.001034798  0.001043062  0.001048524  0.001052214  0.001060322  0.001061056  0.001064965  0.001068414  0.001069027  0.001073791  0.001080305  0.001081312  0.001085925  0.001088811  0.001089036  0.001091301  0.001091884  0.001106338  0.001106781  0.001110067  0.001110067  0.001111063  0.001116678  0.001116678  0.001116678  0.001117409  0.001125324  0.001126632  0.001132617  0.001133424  0.001140267  0.001147598  0.00114803  0.001157318  0.001159026  0.001161359  0.001161359  0.001165182  0.001165271  0.001166841  0.001167115  0.001167487  0.001170179  0.001179194  0.00117961  0.00117961  0.001184725  0.001185874  0.001203002  0.001203002  0.001203002  0.001203002  0.001206199  0.001227545  0.001229815  0.001231886  0.001233877  0.001241983  0.001243165  0.001243165  0.001253658  0.001256999  0.001258416  0.001258659  0.001261728  0.001262857  0.00127272  0.001278923  0.001291334  0.00129263  0.001299933  0.001303974  0.001303974  0.001310747  0.001312796  0.00132021  0.00132021  0.00132021  0.001326263  0.001328098  0.00133328  0.001336401  0.00133702  0.001339177  0.001340788  0.001350522  0.001350988  0.001350988  0.001355029  0.001356234  0.001361862  0.00136789  0.0013738  0.0013738  0.001373937  0.001374288  0.001377341  0.001377728  0.001392698  0.001400833  0.001400833  0.001404998  0.001406876  0.001411704  0.001418234  0.001428872  0.001430701  0.001433111  0.001437569  0.001439512  0.001440337  0.00144084  0.001441558  0.001441558  0.001442109  0.001449283  0.00145527  0.001456236  0.001460888  0.001464564  0.00146781  0.001475966  0.001475966  0.001480961  0.001483226  0.001485522  0.001497314  0.001497314  0.001497887  0.001501763  0.001507393  0.001536344  0.001538451  0.001542733  0.00154716  0.001551693  0.001552089  0.001552401  0.00155708  0.00155708  0.001560654  0.001560713  0.001560713  0.001566249  0.001578108  0.001580368  0.001591489  0.001599444  0.001600203  0.001606003  0.001606003  0.001615209  0.001618547  0.00163197  0.001635082  0.001655281  0.001658486  0.001659707  0.001668064  0.001671407  0.001680911  0.001680911  0.00168732  0.001690134  0.001696592  0.001696592  0.001696592  0.001696592  0.001705286  0.001709512  0.001709821  0.001709821  0.00171337  0.001718063  0.001720238  0.001726509  0.001736817  0.001741464  0.001742001  0.001748879  0.001751663  0.001752036  0.001753556  0.001768559  0.001770589  0.001788367  0.001790095  0.001791066  0.001793845  0.00180125  0.001806017  0.001807013  0.001811631  0.001811631  0.001812737  0.00181539  0.001816605  0.001817987  0.001818505  0.001818742  0.001823828  0.001824061  0.001833322  0.001834422  0.001836381  0.00185302  0.00185302  0.001854298  0.001871116  0.001875443  0.001888466  0.001892935  0.001895477  0.001895754  0.00189894  0.001903066  0.001905872  0.001908136  0.00191237  0.00191302  0.001920039  0.001929695  0.001933942  0.001933989  0.00193463  0.001945205  0.001958934  0.001965364  0.001967641  0.001970283  0.001972032  0.001972032  0.001979344  0.001980207  0.001983389  0.001984809  0.001991341  0.001999552  0.002000706  0.002008379  0.002008379  0.00201502  0.002018339  0.002026983  0.002028924  0.002036641  0.00203853  0.00203853  0.002043443  0.002047263  0.002054587  0.002056421  0.002058613  0.002064116  0.002067925  0.002071626  0.002075743  0.002083187  0.002085912  0.002096046  0.00210894  0.002126135  0.002127082  0.002127082  0.002127082  0.00214516  0.002145747  0.002145747  0.002153111  0.002168057  0.002176592  0.002178502  0.00217854  0.002184237  0.002189237  0.002191666  0.00219202  0.002195002  0.002196316  0.002199154  0.002212323  0.002212323  0.002212323  0.002214312  0.002214312  0.002219474  0.00222638  0.002228816  0.00223265  0.002239003  0.002240202  0.002242555  0.002242633  0.002254362  0.002300994  0.002314702  0.002321164  0.002321164  0.002323705  0.002332085  0.002334251  0.002335669  0.002355649  0.002355684  0.002357817  0.002367257  0.00236878  0.002381816  0.002390155  0.002421486  0.002422477  0.002429953  0.002429953  0.002453945  0.002468067  0.002468067  0.002468067  0.002471061  0.002479269  0.002481713  0.002487618  0.002512693  0.002522032  0.002527805  0.002533724  0.002547089  0.002551753  0.002551753  0.002553565  0.002560914  0.002563316  0.002563316  0.002564087  0.002564433  0.002565499  0.002568701  0.002568701  0.002572226  0.002577778  0.002578418  0.002584046  0.002587886  0.002593885  0.002594938  0.002598491  0.002598673  0.002608139  0.002624919  0.002624919  0.002633006  0.002640731  0.002647168  0.002655811  0.002656827  0.002656827  0.002684517  0.002684517  0.002685194  0.002685206  0.002685842  0.002693375  0.002707155  0.002710213  0.002720018  0.002726426  0.002737038  0.002741045  0.0027527  0.002752895  0.002753431  0.002754035  0.002761375  0.002776329  0.002781993  0.002788496  0.002798902  0.002798902  0.00280578  0.002806324  0.002813985  0.002836435  0.002853551  0.002855703  0.002858351  0.002858872  0.002859598  0.002881075  0.002891243  0.002902402  0.002902402  0.002905125  0.002906842  0.002933994  0.002939211  0.002939293  0.002939293  0.002940318  0.002940318  0.002940318  0.002944217  0.002948521  0.002970884  0.002976636  0.002979364  0.002991035  0.003003007  0.003019028  0.003020168  0.003045548  0.00304716  0.003056102  0.003058876  0.003081951  0.003082371  0.003093861  0.003097308  0.003102203  0.003107613  0.003109677  0.003116278  0.003125059  0.003129833  0.003144161  0.003146468  0.003149051  0.003155748  0.003157749  0.003158021  0.003159507  0.003164028  0.00317763  0.003187057  0.00319291  0.003199432  0.003200378  0.003209204  0.003215898  0.003220954  0.003225468  0.003227778  0.003228182  0.003239974  0.003248823  0.003253119  0.003253354  0.003260103  0.003271215  0.003277588  0.003283408  0.003283408  0.003283408  0.003321602  0.003338907  0.003347736  0.003354719  0.003377605  0.003379022  0.003393519  0.003393718  0.003394343  0.003394343  0.003425266  0.003426309  0.003457333  0.003470795  0.003473471  0.003476354  0.003529802  0.003531556  0.003537582  0.00354099  0.003543507  0.003544115  0.003547986  0.003562805  0.00356408  0.003572781  0.003574829  0.00358092  0.003591329  0.003592406  0.003594075  0.003617163  0.003635198  0.003636443  0.003649261  0.003649261  0.003653063  0.003660438  0.003669185  0.003671985  0.003686271  0.003690354  0.003696818  0.003698446  0.003700489  0.003703702  0.003721207  0.003727719  0.003731395  0.003742066  0.003744231  0.003755936  0.003793677  0.00380054  0.003813989  0.003817447  0.003819999  0.003827954  0.003829043  0.003829818  0.003847342  0.003879669  0.003894041  0.003895502  0.003907332  0.003913603  0.003924861  0.003933038  0.003943296  0.003959355  0.003960721  0.003967561  0.003967561  0.003978458  0.004023019  0.00405454  0.004062656  0.004067575  0.004068265  0.004068741  0.004068905  0.004086568  0.004086674  0.004090016  0.004092266  0.004099781  0.004106274  0.004111701  0.00411625  0.004123571  0.004123623  0.004123623  0.004128427  0.004132745  0.004149364  0.004169858  0.004170735  0.004172524  0.004179791  0.004185051  0.004201447  0.004211436  0.004227349  0.004235685  0.004285014  0.0042865  0.004287143  0.004289891  0.004296963  0.004302265  0.004307615  0.004311429  0.004324  0.004340702  0.004353278  0.004360487  0.004360487  0.00438102  0.004386002  0.004386262  0.004387589  0.004387602  0.004418858  0.004421509  0.004444685  0.004447192  0.004450795  0.004454313  0.004462011  0.004462309  0.004471  0.004476955  0.004477955  0.004479997  0.004480434  0.004485315  0.004512845  0.004532586  0.004536002  0.00453668  0.00453668  0.004566533  0.004574458  0.004574832  0.004584623  0.004599557  0.004603198  0.004608212  0.004609345  0.00460992  0.00460992  0.004620725  0.004620725  0.004644654  0.004649454  0.004655575  0.004657177  0.004673131  0.004674245  0.004684924  0.004685264  0.00469977  0.004701049  0.004712953  0.004726192  0.004732054  0.004740052  0.004757702  0.004782122  0.004782122  0.004798686  0.004826313  0.004835258  0.004837417  0.004851151  0.004867068  0.004867068  0.004873446  0.004877984  0.004894929  0.004896647  0.004901926  0.004902708  0.004909429  0.00491068  0.004929233  0.004929233  0.004950049  0.004958788  0.004978893  0.005001474  0.005024248  0.00504367  0.005052715  0.005053655  0.005061088  0.005066189  0.005073544  0.005077865  0.005092298  0.005092298  0.00509805  0.005110911  0.005125352  0.005125352  0.005132887  0.005143438  0.005147877  0.005164068  0.005168607  0.005171348  0.005175819  0.005180081  0.005188527  0.005189731  0.005197141  0.005222149  0.005228306  0.005236183  0.00524062  0.005241654  0.005268544  0.005268866  0.005283472  0.005283472  0.005283638  0.005307144  0.005317173  0.005337756  0.00535766  0.005360746  0.005362825  0.005364837  0.005380691  0.005383121  0.005383684  0.005383684  0.005386424  0.005390024  0.005391081  0.005400645  0.005405957  0.005410067  0.005412316  0.005418299  0.005442446  0.005456157  0.005474074  0.005474074  0.005476923  0.00548012  0.005492978  0.005501064  0.005504124  0.005534465  0.005535383  0.005552358  0.005576957  0.005584107  0.005590488  0.00560594  0.005621993  0.005627728  0.00562799  0.005634561  0.005641002  0.005643692  0.005673116  0.005677099  0.005677099  0.00567801  0.00571073  0.005721273  0.005738529  0.005744029  0.00574741  0.005759288  0.005770635  0.005771247  0.005787838  0.005788948  0.005820744  0.005825252  0.005838376  0.005856687  0.005874762  0.005874762  0.00587524  0.005877327  0.005886556  0.005898218  0.005911506  0.005920716  0.005922727  0.005922727  0.005923752  0.005926362  0.005930203  0.005980876  0.005998542  0.006020179  0.006026659  0.006036156  0.006060221  0.006077965  0.006084145  0.006091911  0.006108578  0.006129253  0.00613205  0.006157079  0.006157971  0.006166583  0.006182935  0.006183136  0.006183136  0.006183136  0.006205025  0.006205906  0.006255803  0.00625875  0.00626868  0.006272424  0.006272424  0.006274862  0.006293931  0.006293931  0.006309834  0.006321757  0.006326737  0.006326737  0.006341956  0.006354309  0.006373925  0.006381141  0.00638722  0.006399881  0.006406151  0.00642039  0.00642039  0.006436877  0.006458044  0.006477952  0.00647876  0.006525961  0.006530337  0.006530337  0.0065557  0.006583693  0.006583843  0.006590425  0.006602461  0.006602461  0.006602461  0.00660824  0.006642868  0.006645852  0.006648964  0.006658429  0.0066828  0.006688178  0.006712327  0.006728258  0.006744544  0.006751323  0.006757468  0.006778697  0.006787358  0.006794016  0.006808502  0.006836937  0.006840644  0.006844992  0.006855927  0.006859707  0.006861354  0.006884875  0.006886763  0.00689965  0.00689965  0.006917678  0.006920997  0.006934117  0.006964246  0.00697066  0.006975306  0.006979464  0.006994804  0.007019138  0.007047613  0.007050138  0.007071927  0.007072528  0.007075809  0.007077237  0.007077714  0.007080551  0.007094503  0.007098407  0.007114783  0.007120491  0.007120491  0.007127795  0.007127795  0.007127795  0.007131562  0.007131562  0.007166254  0.007182497  0.007219931  0.007279392  0.007293701  0.007312035  0.007312035  0.007312035  0.007313254  0.007315406  0.007330688  0.007330943  0.007360073  0.007366215  0.007398211  0.007398211  0.007403833  0.00740775  0.007439206  0.007451306  0.007451306  0.007487455  0.007504972  0.007567378  0.007585901  0.007606582  0.007610417  0.007618207  0.007622871  0.007631619  0.007645016  0.007660861  0.007660861  0.007661253  0.007686144  0.007704501  0.007709524  0.007712221  0.007717252  0.007719073  0.007737883  0.007738903  0.007762569  0.007766046  0.007766963  0.00777177  0.007780815  0.007800475  0.007805752  0.007809612  0.00781918  0.007841329  0.007847314  0.007855738  0.007855738  0.007872114  0.007881062  0.007882505  0.007891892  0.007934534  0.007959541  0.007960109  0.008005515  0.008029107  0.008049303  0.008089017  0.008104802  0.008115208  0.008132916  0.008179835  0.008185469  0.008188143  0.008188143  0.008188143  0.008202846  0.008212647  0.008239613  0.008253752  0.008261342  0.008272795  0.008280561  0.008280561  0.008287861  0.008290296  0.008290296  0.008290638  0.008296407  0.008296722  0.008305764  0.008305764  0.008317615  0.008317878  0.008317878  0.008317878  0.0083281  0.00834365  0.008346366  0.008346366  0.008369997  0.00837003  0.008381648  0.008384852  0.008400315  0.008400315  0.008405825  0.008465809  0.008579874  0.008636721  0.008636721  0.008646885  0.008646885  0.008675991  0.008676762  0.008676762  0.008695281  0.008744331  0.008761536  0.008797928  0.008812155  0.008842471  0.008845024  0.008884713  0.008899417  0.009015325  0.009016762  0.0090717  0.009071761  0.009071761  0.009116392  0.009158285  0.009158285  0.009159144  0.009175571  0.009175968  0.00921916  0.009248338  0.009248338  0.009268723  0.00926946  0.009287766  0.009308307  0.009339587  0.009347973  0.009371414  0.009371414  0.009372097  0.009386335  0.009391446  0.00941275  0.009438461  0.009485821  0.009534939  0.00954307  0.009558377  0.009563023  0.009605827  0.009607692  0.009629718  0.009634628  0.009668637  0.009680712  0.009689479  0.009696563  0.009702849  0.009709443  0.009731033  0.009739296  0.009745956  0.009749683  0.009754804  0.009758584  0.009758584  0.009758584  0.009773439  0.009785465  0.009850522  0.009859224  0.009867279  0.009880061  0.009884379  0.009921695  0.00993665  0.009959272  0.0099728  0.009982307  0.009987942  0.009990922  0.009997395  0.010009247  0.010015021  0.010022475  0.010051315  0.01006244  0.010091413  0.01009772  0.010102926  0.010102926  0.010129198  0.010175661  0.010179494  0.010230586  0.010292306  0.010330694  0.010392828  0.010410021  0.010410021  0.01041855  0.010432773  0.010465029  0.010465029  0.010465029  0.010465029  0.010467831  0.010467847  0.010502135  0.010534571  0.010554729  0.010579604  0.010595221  0.010604162  0.010629984  0.01063125  0.010652221  0.010661719  0.010663005  0.010677035  0.010699026  0.010749179  0.010823569  0.010824391  0.010847226  0.010854547  0.010876754  0.010877531  0.010930879  0.010943569  0.010946693  0.010973927  0.011004578  0.011011025  0.011084357  0.011093771  0.011118328  0.011133769  0.01117405  0.011255685  0.011294984  0.011331122  0.011394792  0.011403741  0.011420729  0.011424319  0.011442497  0.011454361  0.011458461  0.011458461  0.011471705  0.011504951  0.011517266  0.01156325  0.011619186  0.011619186  0.011632856  0.011687685  0.011741342  0.011811872  0.011836519  0.011836519  0.011836519  0.011836519  0.011836519  0.011857339  0.011863752  0.011865118  0.011881893  0.011886708  0.011914747  0.011934777  0.01196785  0.012018047  0.012019302  0.012036044  0.012036044  0.012052653  0.012058984  0.012061919  0.012084174  0.012093594  0.012097411  0.012105049  0.012129574  0.012153188  0.012180646  0.012189508  0.012209011  0.012210064  0.012227871  0.012228406  0.012269219  0.012302888  0.012302888  0.012305178  0.012312378  0.012312378  0.012312378  0.012315756  0.012315756  0.012315756  0.012320627  0.012351917  0.012375507  0.012375507  0.012380455  0.012385343  0.012411132  0.012425851  0.012425851  0.012482758  0.012511395  0.012512833  0.012565246  0.012585972  0.012622788  0.012627158  0.012640141  0.012653231  0.012678044  0.012686601  0.012696558  0.012701663  0.01275238  0.01278592  0.012790908  0.012815377  0.012833896  0.01285467  0.012859881  0.012868119  0.012880455  0.012896339  0.012955303  0.012989272  0.012989272  0.012994342  0.013034688  0.013077072  0.013077441  0.013123948  0.013160083  0.01321424  0.013227516  0.013228819  0.01325142  0.013262953  0.013327143  0.013346975  0.013349724  0.013378738  0.013384016  0.013392871  0.013401298  0.013401298  0.013425693  0.013435439  0.013436494  0.013442844  0.013475287  0.013536433  0.0135486  0.013562879  0.013583177  0.013591601  0.013598564  0.013598564  0.013606362  0.013606362  0.01364078  0.013688005  0.01369975  0.013703033  0.013703033  0.01374824  0.01374824  0.013767303  0.013778668  0.013785341  0.013785799  0.01381721  0.01381721  0.01381721  0.013825578  0.013844516  0.013855642  0.013871689  0.013890937  0.013890937  0.013890937  0.01392046  0.013975829  0.014008153  0.014014909  0.014108677  0.014151736  0.014173403  0.01417694  0.014177014  0.014192107  0.014204785  0.014277954  0.014297336  0.014297336  0.014371349  0.01438016  0.01438016  0.01440072  0.014426244  0.014426244  0.01446347  0.014470311  0.0145315  0.014540024  0.014599017  0.014602  0.01460259  0.014620624  0.014663948  0.014676471  0.014679412  0.014732261  0.014745669  0.014749389  0.014783401  0.014794525  0.01482998  0.01484771  0.014851982  0.014987783  0.014987783  0.014996419  0.015003022  0.015003022  0.015003022  0.015035734  0.015051234  0.015054523  0.015059051  0.015137078  0.015138289  0.015138289  0.015212281  0.015282869  0.015310936  0.015322305  0.015322558  0.015332495  0.015332495  0.01534026  0.015381251  0.015393096  0.01543796  0.015439216  0.015439382  0.015454679  0.015481964  0.015490022  0.015490022  0.015499595  0.015499595  0.015515666  0.015515666  0.015534038  0.015577629  0.015601296  0.015601296  0.015614596  0.015620597  0.015639568  0.015661776  0.015679638  0.015687692  0.015695531  0.01570794  0.015714994  0.015726031  0.015757133  0.015765363  0.015765662  0.015905859  0.015905859  0.015919026  0.016009384  0.016009384  0.016009384  0.016032894  0.016034283  0.016083917  0.016090275  0.016096249  0.016148572  0.016160304  0.016260205  0.016260986  0.01631737  0.016323567  0.016366913  0.016387248  0.016387248  0.016399303  0.016399546  0.016404306  0.016439146  0.016504013  0.016510121  0.016524144  0.016524144  0.016532185  0.016595711  0.016601619  0.016601619  0.016601619  0.01662089  0.016716538  0.016770366  0.016785293  0.01679564  0.01680407  0.016820108  0.016821855  0.016834355  0.016881062  0.016881062  0.016927927  0.016937712  0.016952769  0.016976622  0.016985301  0.017035675  0.017035675  0.017036418  0.017046712  0.017046712  0.017046712  0.017046712  0.017046712  0.017089625  0.017098095  0.017104498  0.017104498  0.01711192  0.017113825  0.017170259  0.017321962  0.017372349  0.017441153  0.017472689  0.017486206  0.017549679  0.017628906  0.017770076  0.017813712  0.017835936  0.017927411  0.01794287  0.01794287  0.017949003  0.017960293  0.017972707  0.017976283  0.017994966  0.018085952  0.018165349  0.018184595  0.018194842  0.018221122  0.018248213  0.018255103  0.018263612  0.018264914  0.018267517  0.018276459  0.018303091  0.018303091  0.018314149  0.018331827  0.018371601  0.018411828  0.018465664  0.018500062  0.018652441  0.018652441  0.018660426  0.018724005  0.018731086  0.018731086  0.018735333  0.018840303  0.018840303  0.01886617  0.018907625  0.018913349  0.018933475  0.018941112  0.018957184  0.018981874  0.019001597  0.019021385  0.019021385  0.019042765  0.01909192  0.019097703  0.019109228  0.019109228  0.019222129  0.019247047  0.019283682  0.019328356  0.019334045  0.019369508  0.019447086  0.019447086  0.019447605  0.019493515  0.019504251  0.019518347  0.019518347  0.019526449  0.019530116  0.019540276  0.019541586  0.019573721  0.019573721  0.019574812  0.019650059  0.019656376  0.019666705  0.019678568  0.019772176  0.019805818  0.019866012  0.019866012  0.019867222  0.019867222  0.019886939  0.020008145  0.020035999  0.020049141  0.020058845  0.020059537  0.020063539  0.020117807  0.020117807  0.020215054  0.020257884  0.020351288  0.020428655  0.020428655  0.02043883  0.02059533  0.020595656  0.020611916  0.020620549  0.020628876  0.02069436  0.020702477  0.02070698  0.020807955  0.020849248  0.020849248  0.020885736  0.020885736  0.020885736  0.020885736  0.020906545  0.020962324  0.020969727  0.021010566  0.021015896  0.021017983  0.021030382  0.021030382  0.021053738  0.021074758  0.021077429  0.021151464  0.021164307  0.021169583  0.021185554  0.021234431  0.021245332  0.021260949  0.021269518  0.0213287  0.0213287  0.021338646  0.021338646  0.021372684  0.021413959  0.021436539  0.02144266  0.021551568  0.0215788  0.021578951  0.021661426  0.021668795  0.021694089  0.021721131  0.021733525  0.021808072  0.021808072  0.021808072  0.021808072  0.021831298  0.021865729  0.021897414  0.021949155  0.021975416  0.021980463  0.022000922  0.022000922  0.022014374  0.022020424  0.022020424  0.022084359  0.022143472  0.022209603  0.022271357  0.022297627  0.022315229  0.022329883  0.022346517  0.022384547  0.022452003  0.022521574  0.022543764  0.022568367  0.022582915  0.022591767  0.022591767  0.022606125  0.022676439  0.022676439  0.022719916  0.022748072  0.022769998  0.022813378  0.022813378  0.02281437  0.022827333  0.022879647  0.022928902  0.022950493  0.022967118  0.023047441  0.02308122  0.023181162  0.023217662  0.023217662  0.023237443  0.023303334  0.023303334  0.023322592  0.02333905  0.02333905  0.023339884  0.023356979  0.023374125  0.023431784  0.023447222  0.023447222  0.023472748  0.02361143  0.02361143  0.023628457  0.023704758  0.023732052  0.023735692  0.023769443  0.023769443  0.023769443  0.023769443  0.023769443  0.023840045  0.023840637  0.023882966  0.023889055  0.023912573  0.023979356  0.024021305  0.024060733  0.024084945  0.02408694  0.024092273  0.024134134  0.024149199  0.024149199  0.024223399  0.02422766  0.024363737  0.024373736  0.024442118  0.024452704  0.024526742  0.024583592  0.024614248  0.024614248  0.024636444  0.024679107  0.02474691  0.024749022  0.024776834  0.024809393  0.024813798  0.024813798  0.024844593  0.024855069  0.024860184  0.024864594  0.024864594  0.024912764  0.024922363  0.024945664  0.024951518  0.025076935  0.025078136  0.025079426  0.025101906  0.025109932  0.025121313  0.025135237  0.025158247  0.025275649  0.025275649  0.025275649  0.025381092  0.025407507  0.025440956  0.025442335  0.025464933  0.025478592  0.025478592  0.025478592  0.025522012  0.025571914  0.025571914  0.025579343  0.025625701  0.025629778  0.025646997  0.025646997  0.025735164  0.025806416  0.025806416  0.025839815  0.025916957  0.025972065  0.02599537  0.02599537  0.026008857  0.026079837  0.026102772  0.026108678  0.026151622  0.026183779  0.026209244  0.026221856  0.026246054  0.026264361  0.026291859  0.026323902  0.02633339  0.026344757  0.026368932  0.026432416  0.026476112  0.026476112  0.026476112  0.026476112  0.026480911  0.026492408  0.026508425  0.026510533  0.026550149  0.026550149  0.026707488  0.026810698  0.026821183  0.026833793  0.026857474  0.026888167  0.026897139  0.026909325  0.026983399  0.026983399  0.027032525  0.02704546  0.027084516  0.027092627  0.027093677  0.027093677  0.027122702  0.027122702  0.027173875  0.027173875  0.027177614  0.027177614  0.027241867  0.027250841  0.027258695  0.027261115  0.02737875  0.027504001  0.02754501  0.027562103  0.027597182  0.027682881  0.027682881  0.027694789  0.02769617  0.02769617  0.02769617  0.027784084  0.027784105  0.027793276  0.027821005  0.027831191  0.027849568  0.027868511  0.027947152  0.027962286  0.02798761  0.02798761  0.027990427  0.0279972  0.028065542  0.02807076  0.02807076  0.0281238  0.028168541  0.028209339  0.028223991  0.02825163  0.028275414  0.028297998  0.028299954  0.028427969  0.028490459  0.028502429  0.028612752  0.028656942  0.028711526  0.028711526  0.028730309  0.028730309  0.028778929  0.028797442  0.028819183  0.02882198  0.028828305  0.0288373  0.028884611  0.02889569  0.028907075  0.028960225  0.029083361  0.029083361  0.029124773  0.02914264  0.02914264  0.02922239  0.029278054  0.029409271  0.029416289  0.029496089  0.029635449  0.029650777  0.029652123  0.029697483  0.029705501  0.029743915  0.029767736  0.029859421  0.029933926  0.029985869  0.029989709  0.030007594  0.030071027  0.03009773  0.030107645  0.030117781  0.030119384  0.03015461  0.030158665  0.030197379  0.030198307  0.030234169  0.030246318  0.030331552  0.03035418  0.030428379  0.030428379  0.030506976  0.030508368  0.03051924  0.030639296  0.030672496  0.030678416  0.030736491  0.030736491  0.03085205  0.030874917  0.03088462  0.030891076  0.030918788  0.030920895  0.031005206  0.031011771  0.031049396  0.03107157  0.031094097  0.031166215  0.031178005  0.031178005  0.031195662  0.031195662  0.031195662  0.031197423  0.031237022  0.031237022  0.031251287  0.031265199  0.031345742  0.031408712  0.031427679  0.031480361  0.031480361  0.031491273  0.031563182  0.031581283  0.031581283  0.031596251  0.031599277  0.031599277  0.031599277  0.031667538  0.031667538  0.031718295  0.031794996  0.031828339  0.031896384  0.031937433  0.031937433  0.031962611  0.032022688  0.032028332  0.032028332  0.032121844  0.032125549  0.032151219  0.032287446  0.032310258  0.032310258  0.032310258  0.032310258  0.032310258  0.032317058  0.032381894  0.0323821  0.032391829  0.032471946  0.03249459  0.03249459  0.032505796  0.03252275  0.032575418  0.032593693  0.03263988  0.032672875  0.032680501  0.032705227  0.032705229  0.032724358  0.032761442  0.03277709  0.03277709  0.032796106  0.032818044  0.032818044  0.032818044  0.032830542  0.032830542  0.032880512  0.032888767  0.032888767  0.032888767  0.032890856  0.032989897  0.032993437  0.033030006  0.033102851  0.033109085  0.033161812  0.033223038  0.033317197  0.033351928  0.033459795  0.033464811  0.033465327  0.033552244  0.033616213  0.033698197  0.033730875  0.033730875  0.033768645  0.033811354  0.033831249  0.033848114  0.033874612  0.033874612  0.03388851  0.033951936  0.033993808  0.03409864  0.03409864  0.03409864  0.034143783  0.034143783  0.034143783  0.034219669  0.034250575  0.034253876  0.034309401  0.034463817  0.03448642  0.034510918  0.034525058  0.034547901  0.034584394  0.034622879  0.034622879  0.034646407  0.0346467  0.0346467  0.034688427  0.034725983  0.034725983  0.034725983  0.034725983  0.034739067  0.034739963  0.034819771  0.034827281  0.034827281  0.034914231  0.034924538  0.034924538  0.034937312  0.034942649  0.035077077  0.03509814  0.035114955  0.035114955  0.035268812  0.035273193  0.035301515  0.035301515  0.035301515  0.035396379  0.035462646  0.035477725  0.035481964  0.035502598  0.035502598  0.035534495  0.035546924  0.035577316  0.035591387  0.035613289  0.035681508  0.035714854  0.035829192  0.035833251  0.035887639  0.035942632  0.03598051  0.036092869  0.036202715  0.036228427  0.036252455  0.036252455  0.036259601  0.036278617  0.036287668  0.036295595  0.036476702  0.036490571  0.036537343  0.036537343  0.036673743  0.036673743  0.03671671  0.036718796  0.036718796  0.036759526  0.036789102  0.036799619  0.036800159  0.036851709  0.036910796  0.036964805  0.036971224  0.037041928  0.037101632  0.037177393  0.037194689  0.037194689  0.037256323  0.037268303  0.037279371  0.037301518  0.037314966  0.037331096  0.037357848  0.037430674  0.037499218  0.037616856  0.037675197  0.037927469  0.037946827  0.037946827  0.037985284  0.038022334  0.038044483  0.038071078  0.038101286  0.038110839  0.03817395  0.03817395  0.03818237  0.038189109  0.038274616  0.038302137  0.038305103  0.038388935  0.038388935  0.038393449  0.038431313  0.038431313  0.038431345  0.038502514  0.038542915  0.038543091  0.03867829  0.038755158  0.038854122  0.038891766  0.03890671  0.039011059  0.039030992  0.039035103  0.039175319  0.039180266  0.039292701  0.039462861  0.039490944  0.039490944  0.039563923  0.039569711  0.039569711  0.039796007  0.039834828  0.03992511  0.03996004  0.039982034  0.039982034  0.039993796  0.040010594  0.040034688  0.040052026  0.040084394  0.040084394  0.040232301  0.040238251  0.040325707  0.040325707  0.040359966  0.040359966  0.040429321  0.040569012  0.040576221  0.040701663  0.040747905  0.040782709  0.040790982  0.040792444  0.040796704  0.04080692  0.040824261  0.040829187  0.040874696  0.040954183  0.041006927  0.041006927  0.04104289  0.041143722  0.041170678  0.04123778  0.041243593  0.041285695  0.041323606  0.041339927  0.041345836  0.041368345  0.041480714  0.041486404  0.041551115  0.041573081  0.041632297  0.041695693  0.041718096  0.041718876  0.041718876  0.041718876  0.041739453  0.041951406  0.041964745  0.041964745  0.042030076  0.042043599  0.042084152  0.042143887  0.042366525  0.04238608  0.042461617  0.042483723  0.04249742  0.04250425  0.042707783  0.042901499  0.042931265  0.042996196  0.043019522  0.043136427  0.043164425  0.043190729  0.043254938  0.043254938  0.043278275  0.04332961  0.043368536  0.043472513  0.043477412  0.043484757  0.043493423  0.043579683  0.043664886  0.043673601  0.043726952  0.043794609  0.043836006  0.043979005  0.04398806  0.04398806  0.04398806  0.044168387  0.044168387  0.044172576  0.044183302  0.044183302  0.044183302  0.044353576  0.044353576  0.044386694  0.04439217  0.044452115  0.044506507  0.044536824  0.044543012  0.044583265  0.044583798  0.044583798  0.04466836  0.04466836  0.04467309  0.044777709  0.044867186  0.044881754  0.044891237  0.045195192  0.045201559  0.045258462  0.045376343  0.04539847  0.045422713  0.045505513  0.045551538  0.045589089  0.045823632  0.045887366  0.045935214  0.046019836  0.04602306  0.046048449  0.046089965  0.046268168  0.046332903  0.046403849  0.046403849  0.046403849  0.046403849  0.046494396  0.046615991  0.046749985  0.046764212  0.046764212  0.046863301  0.046878717  0.046904965  0.046951703  0.047011237  0.047097564  0.047118031  0.047141679  0.047212096  0.04722691  0.04724811  0.047253987  0.047253987  0.047280217  0.047305131  0.047326203  0.047598216  0.047699205  0.047713376  0.047796452  0.047796452  0.047836739  0.047836739  0.047839219  0.047839732  0.047897259  0.047937839  0.04804676  0.048058506  0.048058506  0.048068204  0.048082171  0.048294643  0.048324389  0.048371029  0.048414645  0.048449187  0.04884276  0.048878282  0.048878282  0.048917485  0.048945852  0.048945852  0.048950486  0.048971825  0.049003082  0.049014449  0.049018568  0.04905017  0.04909537  0.049107233  0.049124193  0.049188649  0.049236057  0.049305221  0.049436134  0.049439158  0.049447425  0.04951311  0.049550759  0.049558686  0.049566267  0.049583509  0.049596664  0.04970162  0.04970162  0.049736849  0.04992523 | 1.937144125  -1.832090848  2.299160309  1.979951404  2.688219765  1.943061488  -1.492693132  1.881111572  1.146511335  1.766245092  1.471145647  1.342585377  1.519995527  1.265718634  1.267406642  1.758876619  -1.445689005  1.868451839  0.942743044  1.082869538  1.041879277  1.530190054  1.192016973  1.103609853  1.411216601  1.306343609  1.381319544  1.86475755  1.329016902  1.735494838  1.294429689  1.45977094  -1.128843984  0.874562121  1.012867819  0.993718213  0.818826358  0.980857975  -1.279576024  1.051475299  1.104556523  -0.928962652  0.904499812  -0.84278168  1.005609392  0.865907363  2.303099879  1.098224292  1.117241654  0.905759211  1.354480831  1.76946375  1.557309715  1.780084115  1.439631686  1.688687784  1.146627167  -1.113173325  1.165940913  0.789876176  1.041522384  0.986314555  0.709983122  -1.051942853  1.26452944  0.773379101  1.072783904  -1.081651918  0.969910263  1.171146034  -0.711131854  -1.056058081  0.746673509  1.114883302  1.035663323  1.13623377  0.586218694  0.661928271  0.838953787  -1.466241679  0.914927827  1.130566538  0.976223358  1.057602661  -1.004557084  0.856735292  -1.397798426  1.059806116  1.094668564  -1.508100743  -0.9637576  1.168269013  0.843511397  1.13578507  1.517836804  0.71118995  1.331080501  1.639570632  0.795895256  1.135488231  0.807771474  0.985796997  1.047927183  1.199078603  0.997906841  1.555861806  -0.836448922  0.895239603  1.06239074  0.904915794  0.949223964  0.840143439  0.855240497  0.863128229  0.825389305  1.163201351  -1.29666023  1.175475737  0.783869034  -0.585180344  0.928185076  0.970542268  0.837874516  0.87357714  0.662478162  -0.704740367  0.751182945  1.177392192  -1.510777931  1.51045869  1.183161484  1.325746958  -0.687486877  1.609216317  0.835168312  0.916900042  0.935400983  -0.870564428  1.961769548  -0.637463025  0.558306135  0.953859317  1.020590818  0.807854931  1.241019007  -0.878354314  1.231699263  -0.673508796  -1.063092609  0.717419935  0.728183728  0.897559343  -0.671771088  -0.734565935  -0.594623675  1.439944797  0.841473233  0.673521569  0.91123685  1.33184658  1.329331033  1.307483181  0.72008683  -1.006054165  -1.262222208  0.973361132  0.820102537  0.677278757  0.759996208  1.043946434  -0.904724795  -0.884820998  -0.890130294  0.830953815  0.794930029  0.866669676  0.641359016  0.83960201  1.051857787  1.67880698  -0.602688884  0.663099229  1.814618585  1.819579932  0.50753895  1.053537651  0.770198746  0.713837435  0.763550744  0.727165705  0.65710851  0.656150695  -1.559379386  -1.195014117  -0.757977145  -1.145454807  0.847156016  0.551382808  0.824382557  0.66963385  1.048512362  1.000286368  -1.311090044  1.769313089  0.860830923  0.985042478  0.669777118  0.703963752  0.839084962  0.75920435  0.815776576  -0.973865531  -0.618600452  0.752941931  0.668760879  -0.55665372  0.959577589  0.854234798  -0.71194867  0.981332633  1.303265257  0.694084515  1.509243697  -0.870995794  -0.69367999  0.652147804  -0.770986736  1.041275895  0.466569694  1.115649959  -1.072306371  -0.732435769  -0.666251887  -0.918557647  -0.897670994  0.577579641  0.997465216  0.861099817  0.878475809  -0.902770071  0.922001847  0.971972149  0.877819888  0.895142519  -0.77821495  0.572562037  0.994545193  0.425999456  -0.97272047  0.65433251  2.205163179  -0.579968197  -0.806984022  -0.5166265  0.67922602  -0.809396284  0.664312561  0.660270837  0.736785568  -0.622357792  0.930940085  0.574869469  -0.538593901  1.092380648  0.700956777  -0.741959653  1.113780513  0.635738761  1.980419662  0.760826266  -0.692189754  -0.694103185  -1.147362214  0.608512207  0.755825675  -0.601985226  0.800683541  0.837999503  0.855359651  -0.572178931  0.45423846  -0.6009178  -0.498582885  0.734408733  0.568024016  0.74049157  0.820140071  1.296051031  -0.61388722  -0.807345909  0.593000847  -0.876282198  1.162893737  0.504726688  0.953741664  0.658801085  -0.572544089  0.446112376  0.665581049  0.849849153  0.598459033  -0.80028176  1.107008793  0.672542802  -0.973987107  -0.837187616  -0.732725134  1.25164352  0.460406839  0.560801193  0.914308243  -0.817349072  0.464061817  0.96955744  0.789036082  0.595025305  0.6409209  -0.67334565  0.64780316  -0.659688539  1.849332483  0.66412073  -0.830409782  0.706740427  1.226868709  0.69430365  0.619385127  0.480708815  0.952535887  0.415076136  1.006090142  -0.603936382  0.600567982  1.010729277  0.878043862  -0.95185304  1.010308498  -0.678326559  1.035016631  -0.83433896  0.601748168  1.125003452  0.672737874  1.211540211  0.842798434  1.002031934  -0.710102503  1.048691555  0.82926135  1.474973131  0.61633226  1.056299722  0.465242216  0.946635312  -1.241917682  0.895661589  0.846247024  0.550299282  0.663882105  0.700822505  0.628169241  0.522820915  0.680149308  0.66059729  -0.402156077  -0.730840657  -0.601708917  0.93147713  -0.44417994  0.83678774  0.882291432  0.659358863  0.460334647  -0.82635935  0.666284713  -0.59435801  0.765652652  0.730099872  0.513883236  -0.995361667  0.510876966  0.928699657  1.715222876  0.489190773  0.591983235  0.621767612  -0.48563167  0.674522983  -0.462626042  0.471126979  0.545776815  -0.493539899  0.570372566  0.46341398  -0.87608125  -1.195300271  -0.718723393  1.049665459  0.633142999  0.835713598  -0.888869314  0.578467468  0.963586133  0.47701369  -0.820543794  0.912520431  -0.616879372  0.694497042  0.602456336  0.685840707  0.422982618  0.658496914  0.679886648  0.433985814  -0.824676032  0.520500888  0.721571524  -0.487433442  0.548660532  0.550900937  -0.692890588  -0.777933154  0.639706185  1.480057684  -0.575248373  -0.496812523  0.644745011  -0.488557412  0.6880759  0.491220514  0.627878366  0.917024026  0.548501352  0.798714275  1.147213993  -0.47153968  0.896641172  1.864926423  1.245982888  0.922841617  -0.476694158  -1.075988267  0.7295206  0.637530819  1.05046193  0.683292637  0.577205566  -0.696287862  0.43512909  -0.573147629  0.561466035  0.688777988  0.919945952  0.854792699  -1.09688585  -0.399124132  1.764582927  0.502971152  0.764299021  0.530896723  1.124445473  0.807569758  -0.65806214  -0.438682748  0.409900453  -1.156689569  1.019675549  0.853352712  2.627869316  -0.926850264  0.523393268  -0.559219263  0.622155862  -0.763783564  -0.585772352  0.810171925  0.844970213  0.57761306  0.531276659  0.768136081  0.849869205  -0.547916737  0.512299973  0.500403064  -0.350752802  0.889261479  -0.665499253  -0.621185026  -1.160435807  0.602430514  0.506270363  -0.492070806  0.607519847  0.738092117  -1.171874608  0.457830505  -0.443297962  0.67981744  1.027333164  -0.967970019  -0.797180907  -0.677629725  -0.785359072  1.229753515  -0.780920219  -0.476234274  -0.61452817  0.449408568  0.519723201  0.686508659  0.771683498  0.749064019  -0.645469583  0.831822534  0.573203299  -0.723175818  1.24903859  0.49146107  0.728933114  0.650707593  2.036872477  0.40356065  0.817479239  0.519785725  0.373474385  0.615961099  -0.699729429  -0.745844731  0.55384894  0.534350624  0.786250935  -0.603903522  1.016655495  0.980782641  0.407814692  0.483721735  -0.63713281  1.590841707  0.446109227  0.818619801  0.953204148  -0.843446064  1.024669049  0.979103921  1.17384448  -1.219532026  0.319869392  -1.655340044  -0.482153517  -0.70169298  -0.696987237  -0.598189339  -0.493162843  0.770168496  0.894161961  0.549211164  -0.402363215  -0.625886797  0.643408788  0.40637844  -0.545441772  0.979046512  -0.571773595  -0.733390772  0.572403894  -0.723974092  0.626822979  0.438950306  0.448370755  -0.798288863  0.555679175  0.341733817  0.530496383  -0.4731932  -0.604813113  0.609031325  0.642445211  1.636313844  0.451973681  0.668487518  0.588435488  0.4945823  0.406235768  -0.500035741  0.725745827  0.633789206  -1.00710486  -0.849004045  0.453250382  -0.461893606  -0.754587078  -0.683133821  1.992104166  -0.825870753  0.470127222  0.644951979  0.710617855  1.174900694  0.760681672  1.200400959  0.744954956  0.338208691  1.73924513  0.657656344  -1.126959158  -0.512654284  0.558571276  0.493384593  0.699248804  -0.735665235  -0.77206037  0.493621295  0.529920098  0.543840213  -0.40857299  0.624003037  -0.456774148  -0.435624039  0.333405859  -0.375792424  -0.503093859  0.428939299  0.366153167  0.540292737  0.552818654  -0.495173639  0.380838597  -1.036524365  -0.673485305  -0.747604567  1.204160962  -0.79783963  -0.742819908  0.421557348  0.803990453  -0.494936759  0.320776047  0.546444247  0.382770182  -0.663979534  0.568229173  -1.040341722  -0.489353759  0.547321399  0.384263316  0.961154262  -0.537056811  -0.583616844  -0.57845274  0.467540732  -0.408846938  -1.090227248  0.65004431  -0.55555957  0.739982497  0.601217117  0.606504401  -0.823952625  0.502203919  0.359567703  0.655358482  0.996584646  0.392330195  0.498190136  0.704062467  0.409726026  0.72111839  0.687390605  0.486649393  -0.476909144  0.641831483  0.557006422  -0.746647004  0.410713897  -0.898462197  -0.567905036  -0.416511791  0.692148115  0.597382491  0.404214869  -1.133750616  0.587927723  -0.718073535  0.699544995  0.605408323  0.404900392  -0.6343496  0.497331461  0.882053987  1.121135683  -0.529175055  -0.975565204  0.428601977  -0.664796623  -0.716329571  0.788735163  0.659556575  0.821987678  0.475029471  -0.509465915  1.029117801  -0.895669628  -0.752346933  -0.617090649  0.451659821  1.150106967  1.071535168  0.403238016  0.361660619  -0.580536893  -0.465737711  0.39778368  -0.635496361  1.123223445  0.62307653  0.40410998  0.728596261  0.688668356  -0.448525825  1.794688966  -0.722477503  0.652190805  0.800844823  -0.439069539  -0.467324986  -0.606184649  0.521702765  0.468846536  0.556590855  -0.798863157  -0.584342548  0.482010903  -0.45336847  0.456244418  -1.185862959  0.433892921  0.64424061  0.396385128  0.366660127  0.390329025  0.412706946  -0.717060581  0.826367576  1.021522894  -0.467175942  -1.086365641  -0.376199644  -0.398222185  -0.55304456  0.545188057  0.610599556  0.910666589  1.05069636  -0.295790851  0.607775583  -0.564825546  -0.374641907  -0.492656639  -0.448722456  0.597568289  -0.585232545  -0.439685019  -0.8238356  -0.625074177  -1.070377074  0.936531097  0.427551542  0.756422745  -0.773797952  0.422098289  0.944908982  0.384663729  0.728356881  -0.65896364  0.920090833  0.522756181  0.71605812  -0.5038612  -0.675023571  0.450237844  0.775081846  0.282411516  0.420734733  -0.650905372  -0.469236406  0.561587067  0.581639879  0.396851285  0.459662392  0.293703187  -0.58776571  0.46722116  -0.779944763  1.123773868  0.470474339  0.538328062  0.801327403  -0.684759552  1.107396329  -0.699082492  0.383492015  0.603237504  0.432592747  -0.404100983  0.450923708  1.02996664  0.669227584  -0.656115619  0.539695415  -0.435948515  0.832551803  -0.465268474  0.386735061  0.534187884  0.453398828  -0.415782197  -0.749792108  0.352431925  -0.29987932  0.896581398  0.45773861  0.520719108  0.511803463  0.763490237  -0.626207398  0.439501212  0.59112922  1.009768778  1.254242292  0.419610049  0.525818351  0.780161208  0.558850159  -0.49185586  0.729102076  0.299064806  -0.542634587  0.4188935  -0.441819302  0.716803957  -0.815556373  -0.477874015  1.082430035  0.669864778  1.191994054  0.757971955  0.532752825  0.578700126  -0.392538812  -0.369389994  0.448888593  -0.62486914  -0.814678062  0.556759283  0.789878247  -0.361288729  -0.592043697  0.436204696  -0.730245027  -0.557661644  0.429131166  0.982279685  0.414524623  0.599082091  0.613463552  0.969772772  0.4736936  0.830851183  0.914434676  0.429216159  0.286304804  -0.443080433  0.496199043  -0.790845677  -0.438088213  0.396492803  0.257123001  -0.95923102  -0.666549281  -0.397848446  0.438916515  0.415681047  -0.47209879  0.86382778  -0.358018671  -1.817158993  0.415132019  -0.350388247  -0.400168015  0.347904269  -0.542854941  -0.79318127  0.295868946  0.541933788  0.659028401  0.55205208  0.36918546  0.609459237  0.618622911  -0.781755694  0.509040483  -0.342386709  0.718116664  -0.497663593  0.532947696  -1.18513689  0.505121607  1.284475054  -0.354794425  0.333603358  0.68278347  -0.573933221  0.343814798  1.260392781  -0.594741334  0.362748931  -0.399114281  0.302289499  0.441135828  -0.520598285  0.452697704  0.328236889  -0.449576191  0.428315357  1.073529761  0.392147189  0.362363517  0.903359527  0.707138025  0.458417258  0.976521153  -0.974901166  -0.398482107  0.423035381  0.331552301  -0.339504338  -0.489790739  0.364780497  0.675116434  0.325970486  0.456221548  -0.802356194  -0.455502969  -0.444545845  0.430270937  -0.989561498  -0.470874367  3.606587067  -0.484340718  0.387715439  0.389129671  0.70994361  0.500418868  -0.785493968  0.452401516  0.645523953  -0.544415779  -0.672515222  -0.410129152  -0.442128265  -0.692692388  0.532411391  -0.588539352  0.597032449  0.296535134  -0.576889616  0.786010005  0.62500714  0.402357513  0.548187769  -0.53312114  -0.960129462  0.449309159  0.294439608  0.503448066  -0.574398902  0.463413099  0.351463715  0.331510768  0.457730592  -0.725009397  -0.511748772  0.623597244  0.389087225  -0.906092449  0.608105102  0.744003601  -0.463030056  -0.347248058  0.372077096  0.430183262  0.665755109  -0.371622036  -0.556802066  0.336905198  0.426625361  0.381986494  1.330298052  -0.375918429  0.449407349  0.374807311  -0.664098988  0.505424705  -0.395005462  -1.688872598  -0.686539829  -0.531233059  -0.343315807  -0.515756375  0.443518398  0.721383908  -0.510269402  0.582371285  0.794582819  0.380287005  0.670644628  -0.657066556  -0.618241599  -0.406883047  -0.656081101  0.548606861  0.521461226  -0.629976844  2.013059159  -0.760350002  -0.709258579  0.546274229  0.434148479  -0.472010028  0.939512588  -1.038362535  -0.639082288  -0.60999889  -0.531839465  0.443537283  -0.464975065  -0.392267018  -0.611002508  0.457650357  1.40135654  0.430583748  0.981345912  0.295687069  0.378844621  -0.5511116  -0.742152642  0.323054564  -0.424860114  0.402108628  0.479658624  0.567241802  0.352623783  0.424655943  0.49565381  1.135241632  0.400353149  0.280507333  -0.555653828  0.84225767  -0.355135826  0.33246286  -0.416565209  0.771047068  -0.3921063  -0.491323449  0.76630154  0.322477606  0.345708585  -0.524437875  -0.412142703  0.539938148  0.550146284  0.550564555  0.485209275  0.4268006  0.805685895  0.512086369  0.785150757  -0.992185569  0.640930715  -0.380917432  -0.714261352  0.529049523  -0.487721546  0.436094428  0.337789984  -0.616796645  -0.47178549  -0.522470453  1.370133599  0.489942368  -0.552835914  -0.539030087  -1.44619016  -0.358748121  -0.65232917  -0.296697112  0.298473504  -0.667276743  -0.487306605  -0.402812039  -0.492777035  0.646762711  0.598556483  0.414863921  0.556923034  0.407389602  0.296951042  0.517726549  0.444388282  0.514461525  0.882825642  -0.344766619  -0.396091257  0.424863979  0.455275575  -0.270691171  1.122717934  -0.682260811  -0.548050219  -0.501543345  0.48487454  -0.362583691  0.930898086  0.738108309  -0.373862071  0.46090569  -0.347780349  0.646595364  -0.567929244  -0.403609653  0.738832505  -0.423444686  -0.481112139  -0.481990089  -0.778858347  0.363284487  0.260936918  0.675497402  0.498673771  -0.464314515  0.346627012  0.304584387  -0.374666573  0.579507761  -0.500090133  0.618924328  -0.462432869  0.41603818  0.392295445  -0.481670398  0.33936144  0.539249586  -0.598370763  -0.306763291  -0.415193294  -0.554754389  -0.332414604  2.516381176  -0.370769138  -0.486308621  0.29303084  -0.557855286  -0.330789018  0.322695496  0.784655337  0.306290721  0.336947563  -0.46123935  -0.278124353  -1.376410847  0.368754162  -0.378324483  -0.383237504  0.376416702  0.33187693  0.379564539  0.523944916  0.896718228  -0.466138757  0.397800811  0.601286943  0.855558567  0.386534174  -0.268470174  -0.986070165  0.729065806  0.382193397  0.411361824  0.446968579  -0.367864505  -0.332915425  0.373363053  0.36407939  0.31132916  -1.130627272  0.347498646  0.573866585  0.368755482  0.40785664  0.407530895  0.299303288  -0.330139153  0.520625312  -0.362526906  0.45104558  -0.446783927  -0.344684502  0.438137102  0.36604199  0.60694962  -0.558743545  0.519984457  -0.296185708  -0.515433106  0.420065801  0.295277798  2.245709804  0.436737015  -0.479630597  0.31096694  0.744651978  -0.237250882  -0.61478052  0.319222506  -0.837475204  -0.376619321  -0.878423665  -0.77451504  0.5139123  -0.452525467  -0.449935114  0.431109558  0.575492291  -0.782762575  0.533505451  -0.520137049  0.456590915  -1.599050123  0.300370068  0.522973497  -0.5362444  -1.041809904  0.438750436  -0.57732477  0.357993879  -0.478281659  -0.354326048  -0.664834081  -0.549151825  -0.60662324  0.642096625  -0.564964455  -0.732935507  1.143373261  0.347404748  -0.484888638  -0.566917918  0.367814957  -0.546946584  0.744261914  -0.626521153  0.274576757  -0.490893609  0.271799608  -0.407280472  0.440397006  0.425440628  0.539816645  -0.911070274  0.290019606  0.370917839  -0.772250136  0.478768946  -0.411680483  0.264827593  -0.356992627  0.29327934  -1.53115274  0.483130905  -0.411137804  0.692218135  0.672640323  -0.360523393  -0.33532413  -0.270511871  0.4138812  -0.744693227  -0.288052392  -0.541351823  0.393400459  -0.562811568  -0.587063371  -0.407213734  0.264618521  -0.62283377  -0.433055638  -0.364528265  -1.501412459  -0.391933084  0.279756746  0.499868  0.485141843  -0.414545485  0.274006572  -1.19793462  -0.402656867  -0.610782531  -0.458950915  -0.369590086  -0.724696233  0.575479914  -0.747577923  0.231883573  -0.332182025  0.414466759  -0.398891762  0.416895579  0.436706097  0.375563856  -0.468558259  -0.487856719  0.517185531  0.450563585  -1.099510232  0.290851866  0.361179212  0.994474187  1.208933633  1.161320375  -0.480736556  -0.54838385  0.414564764  1.245131866  -0.557080458  0.46542453  0.905307682  0.500963439  0.628726662  0.441039572  0.444657682  0.414122098  0.356714631  0.451011204  -0.628006055  -0.451749601  0.6226784  -0.338220578  -0.283353334  0.61531562  0.587414524  0.424762153  -0.475453987  0.533368154  0.444485364  -0.844680226  -0.358417847  0.401235703  -0.831667955  -0.439565427  -0.699355833  -0.321549526  -0.416264331  0.59935776  -0.870526282  0.604337539  -0.795702435  -0.794508204  0.402914441  -0.800226282  -0.89043682  0.410096033  0.575070131  0.243760817  0.407074846  0.603005371  -0.488727716  -0.524851528  -0.361858058  0.322669218  0.572993303  1.163886433  0.288120887  0.736032995  0.612883729  -0.489797569  -0.983791786  0.284936176  -0.386930084  0.338198261  0.391378418  0.589782856  -0.5660109  0.304435036  0.28755706  0.449931273  0.510082899  0.778624499  -0.713425044  0.288958917  0.345875025  0.329272707  0.475392661  0.510605271  0.391097313  -0.382546123  0.512740693  -0.703703523  0.58839136  0.37643152  0.399632733  2.055584962  0.65208252  0.560932917  0.439923322  0.270045818  0.403988062  -0.363773954  0.385307725  0.341276623  0.412563527  -0.410755885  0.75370524  -0.531167051  0.87118544  -0.245452564  0.511713909  -0.386497803  0.574044583  0.429204371  -0.328911165  -0.274221774  0.83203874  0.31653696  0.647799583  -0.640299436  1.89974879  -0.460508858  0.529201716  -0.672103153  0.480826863  -0.370134554  0.451773524  -0.5015197  -0.371012165  0.373506747  -0.404365126  0.692213387  -0.577599266  0.318128801  0.409035959  -0.487275416  -0.57133567  -0.933680887  0.337129107  0.286130427  -0.361815401  -0.382337849  0.352139911  0.505906685  0.389022084  0.968698463  -0.79489752  0.431794365  -0.417059154  -0.342677692  -0.268716176  -0.846703119  -0.733892525  0.518645121  0.210024144  -0.617256646  -0.420479847  -0.351400062  1.984235274  0.37015234  -0.657310622  2.842884712  -0.360629786  0.594179042  -0.672835223  -0.300646779  -0.493580994  0.43784565  -0.820614307  0.398292587  0.342568236  0.297354441  0.34203972  0.336464439  0.365862313  0.389400376  -0.5004609  -0.335622786  -0.68374034  0.454701413  -0.438750449  0.337018851  0.458577026  -0.383105866  -0.560367332  -1.305871347  0.291285315  1.006452902  0.253754132  0.301833801  -0.389742346  -0.30552211  -0.916114892  0.361878231  0.606133642  0.379777801  -0.414603252  -0.548978881  0.603770089  0.293673811  -0.741944392  -0.45990137  0.38495445  0.5128719  -0.565096951  -0.341433624  -0.599212671  0.42082732  0.514960398  0.290307209  -0.420936733  -0.472348011  -0.255459446  0.413489649  -0.402515868  -0.342888958  -1.02560672  -0.476752533  0.313991222  0.533451221  -0.571904911  0.42923103  -0.492153471  -0.40336267  0.440883855  0.323522994  0.34613892  -0.449921478  -0.327148441  0.262163942  0.575683802  0.328711147  0.711927645  -0.560022125  0.273520542  -0.32115709  -0.473210341  -0.339021325  0.720889498  -0.539154523  -0.51393334  -0.492904533  0.427335043  -0.291944018  -0.36568017  0.315865363  0.291186145  0.453483677  0.421815442  0.233454969  0.387304576  -0.548513955  -0.789995993  0.489435688  0.322184687  -0.844894718  0.577061019  -0.858011222  -0.401810349  0.305656706  1.374934265  -0.380277585  0.500677238  0.435260451  -0.53000965  0.273197909  0.945782418  -0.299069006  -0.234111782  -0.401363249  -0.508821156  -0.304542404  0.489483519  -0.557646828  0.440167231  -0.53691343  0.430419963  -0.334518269  0.298508852  0.878876522  -0.311410222  -0.462029113  -0.797360092  0.329410263  0.430316664  -0.576592163  0.433738818  0.412440367  -1.285959348  -0.239431751  0.343342631  -0.830182668  0.311571403  0.383643598  -0.356986666  -0.411668004  0.365128463  -0.526614185  0.355850802  0.284451859  -0.550154023  -0.265923049  0.676727261  0.38509524  0.377415992  0.522881495  0.508245352  0.365193231  0.873960193  0.551684083  -0.497354351  -0.629538431  -0.383007197  -0.432229767  -0.686808717  -0.652965125  0.411362794  0.396337997  0.397350103  -0.741698863  0.441633476  0.480342601  -0.684474505  -0.343783981  0.597068306  0.825970072  -0.442076062  0.866282526  -0.512664411  1.076658372  -0.485862222  -0.514196326  1.366567994  -0.369235804  -0.692931223  -1.022064319  0.372566982  0.40314922  1.081053259  -0.271869368  0.401471567  0.508546121  0.377850192  0.888326849  0.350509789  0.503749254  0.681005077  0.640923435  -0.46860798  0.973542763  0.444543539  -0.329011845  0.261579298  -0.27396331  -0.736371039  0.274383035  0.316334072  0.676584601  -0.350786453  -0.553451178  -0.653692331  -0.459501737  0.298307775  -0.647302889  0.382353852  -0.349057357  0.30846152  0.456498079  -0.245723974  0.5973233  -0.706343415  0.465744283  -0.639158382  0.295215192  0.320456199  0.462832003  -0.251159301  -0.376715573  -0.274449431  -0.37623926  1.188146964  0.47371127  -0.494212905  -0.592913786  -0.30275497  -0.359887477  -0.327619088  -0.964695432  0.324041543  0.306186959  -0.291446263  0.566463937  -0.823053011  -0.455378824  0.329833517  -0.331356311  -0.413987973  0.428208326  0.375338401  -4.41844791  -0.254894581  0.294877396  0.41211202  0.325592598  0.514698258  -0.275481612  -1.043561178  0.35015501  0.761551958  0.589111358  0.924996749  -0.63328741  0.372123945  0.363699212  -0.828495735  0.368530551  0.757783606  0.331322451  -0.238647924  1.370203803  0.369408994  0.320509715  0.347459473  0.388932337  -0.45989271  -0.325783554  -0.357724205  -0.363901385  0.68588817  -0.207006683  0.484279045  -0.728191067  -0.409327645  0.312924023  -0.380357294  -0.615680798  -0.381002072  -0.390673713  -0.408010881  0.47736917  0.366104162  -0.73459602  -0.428630246  0.379748823  0.701490995  -0.340037213  0.775096245  -0.355320657  0.610345918  0.437916171  0.326238721  0.76190755  -0.292162932  0.369207208  -0.265964037  -0.231736313  0.292253973  -0.466040459  0.444040761  -0.345191701  -0.552891719  0.348957938  -0.338721319  -0.621275693  0.463491769  -0.2749065  -0.471143044  -0.65042174  -0.423801229  0.420637702  -0.383867272  0.244901975  0.300314806  -0.273281688  0.324532259  -1.055233909  -0.240626924  -0.220836274  -0.286174805  -0.282688573  0.373908879  0.619317115  0.374153189  0.260113273  -0.223893825  0.361803814  -0.375075743  -0.363615664  -0.52617744  0.260838366  0.240222235  0.44196122  -0.548912492  1.111354033  0.523400977  -0.52977325  -0.607564199  -1.158515407  0.350630721  0.330664222  0.39956396  -0.657440627  0.250874842  -0.768205598  0.247833188  1.229369942  0.241402721  -0.42928174  -0.327167079  0.327278553  -0.248334826  0.255833398  -0.501642361  -0.359155851  0.605197107  -0.246950591  0.349380399  -0.30117357  0.386855055  2.105481283  -0.353106716  -0.312444172  -0.485846486  -0.417693362  0.614940639  0.354674663  -0.31581195  -0.498307932  0.410273033  0.321808043  0.676012394  0.344505838  -0.636854251  -0.311110451  0.669661378  0.507955633  -0.290257496  -0.850009468  0.473767782  -0.413878544  -0.686073379  0.276926994  0.31583257  0.507503514  1.823201066  0.371977815  0.351250239  0.272029711  -0.385334248  -0.353031487  -0.549115221  0.52409647  0.543183479  -0.369134493  0.410817942  -1.072748825  0.347976642  0.405666127  -0.430009824  0.378093122  0.353962578  -0.397338629  0.53150083  0.324911712  0.327645217  -0.733942035  -0.715820948  0.359669837  -0.324944912  0.426044281  -0.443585366  0.407154776  -0.720939126  -0.324862695  0.343496969  0.354613243  0.409508401  -0.772162301  -0.439189947  0.456672357  -0.32132385  -0.333694698  0.437465188  -0.261682209  0.224421391  -0.302748142  -0.233475503  -0.297640778  0.375163013  0.265103977  0.94705928  -1.268798849  0.30691771  0.378061696  -0.37674303  0.668335415  0.261116595  -0.345960407  -0.753355574  1.378008468  1.363865842  0.386843499  0.36928152  0.532393451  0.516052311  -0.342364549  -0.966352108  -0.295975399  0.21875054  0.570995272  -0.497079773  0.385080009  -0.326056866  0.391085763  0.402126189  0.325000065  -1.100867399  0.344431161  0.279965482  0.307835636  0.707221423  -0.452346502  -0.415795914  0.397117844  0.351863449  0.28450058  0.639945211  0.270015524  -0.473912662  0.358238152  0.248916884  0.370709001  0.20457906  -0.2919253  -0.48470309  0.536611466  0.409175463  0.360401607  -0.251103246  0.353739273  -0.449587125  -0.647890774  0.520085603  0.762459722  1.455634009  0.335509034  -0.275139016  0.576577142  -0.391260867  -0.346334831  0.252047824  0.30040476  0.365356237  -0.271635692  -0.388314298  0.20710888  0.280318946  -0.691155202  -0.418587312  -0.272718574  -0.206728079  0.366708496  -0.24566134  -0.436799766  -0.382558638  0.413376667  -0.343825104  -0.267747893  -0.312888986  0.385703326  0.403142657  -0.604357833  0.303157613  -0.752959619  0.183584969  -0.214719234  -0.611304025  -1.439070409  0.186694839  0.94268698  2.438802295  -0.366637884  -0.658875914  0.232659612  0.207452  0.446101124  0.204369117  -0.555977982  -0.351963591  0.258413175  -0.330742207  0.477424037  1.76975073  -0.264376881  -0.792155571  0.229322088  -0.245852927  -0.676664132  2.256628514  0.37471817  1.916677042  0.27335113  -0.473291953  0.507710742  0.933211036  -0.524259792  1.077045517  -0.31613601  -0.988629192  -0.619776126  -0.294362379  -0.370876702  -0.321154867  -0.915230874  -0.676592716  0.253079123  0.33543834  0.563086508  -0.555188455  0.241844502  0.887387354  0.317003642  0.324167418  0.780345822  0.248379576  -0.546413835  0.211545357  0.694785183  -1.18188929  -0.26075628  0.388665558  0.827138029  0.472725894  0.547623876  -0.27556737  -0.819958258  0.331397093  -0.360502987  -0.279183188  0.189422546  -0.436512863  -0.320190383  0.27173246  0.291844754  -0.515045179  0.295391258  -0.669436317  -0.841462129  -0.418204848  1.25876308  -0.809273989  -0.363578237  0.38388282  -0.273252974  0.367460875  0.397032883  0.468217272  -0.539465766  0.545268476  -1.372705965  1.484325682  -0.582226327  0.47727507  -0.533868662  -0.57779471  -0.485870177  0.697079443  -0.248669294  0.303681956  0.461126646  -0.49695075  0.30306894  0.311801146  0.746552963  -0.457215881  -0.288163075  -0.216663493  -0.397827613  0.380311629  -0.293992549  -0.23801832  0.337005306  -0.28915597  -0.281936385  -0.251824729  0.288064688  0.253785434  0.282891229  -0.408496929  -0.795246378  -0.477771758  -0.335524145  -0.558893393  1.644333139  -0.394080411  -0.464570008  0.813028741  -0.315738429  -0.476078688  -0.525954001  0.313360544  -0.393426737  0.700718589  -0.56649635  0.236118996  0.218244204  0.232109057  0.411476379  0.195944791  -0.334410926  0.258856947  -0.197510351  0.369607344  0.248700873  -0.361959765  0.597189143  -0.351830236  -0.316064697  -0.734429118  -0.313760463  0.372385089  0.333000354  0.472393129  -0.355250571  -0.241399814  0.572992581  -0.559781347  -0.269121408  0.25012353  -0.220018575  0.511482056  -0.832924474  0.460581426  -0.321409535  -0.313646788  0.474764812  0.261768357  -0.301544274  0.67760598  -0.488646254  0.287071435  0.363509667  0.410033874  -0.356250063  -0.785286312  -0.513936393  -0.243457962  -0.281990044  -0.402816581  0.211232713  -1.417434783  0.325855464  0.530573703  -0.474421361  0.303047349  -0.814069554  0.396521759  0.336472463  -0.416575569  -0.367630921  -0.38825974  -0.254148626  -0.289798618  -0.591504505  -0.447091507  -0.150282185  0.316939779  -1.249923679  -0.581147056  -0.203254325  -0.313213503  0.29744616  -0.34006399  -0.291447074  0.411471626  0.495545146  0.317215922  0.69673725  -0.515439897  -0.766184168  -0.191126577  -0.303347897  -0.40462769  -0.397846486  -0.317993957  -0.493540618  -0.308338146  -0.323738038  0.279514757  0.301720386  0.310696444  0.344423711  0.177559299  0.297127016  -0.556754077  0.414670722  -0.372732243  -0.299509999  0.311186993  -0.521624122  0.363508878  -0.412031564  0.448015795  0.514636006  -0.314907743  0.495150026  -0.339287277  0.352387708  0.314951251  -0.256485244  0.36718857  -0.765708867  -0.407744081  1.526181144  0.649611916  0.341301352  1.509720725  1.160411009  -0.188301214  -0.287064462  0.451910341  -0.564416973  0.29009864  -0.405444774  1.142298774  0.318964616  -0.501318595  -0.333900385  0.238498641  0.306461899  -0.392992718  0.351512651  0.313510606  0.634655048  0.757154594  0.365246117  1.02077204  0.283278643  -0.377736573  0.372765031  -0.52988158  0.272262729  0.339790179  0.207333636  -0.466661145  -0.407215345  -1.459631781  -0.206082135  0.257375015  -0.604000097  0.411369136  0.280356504  -0.319653721  0.394897428  -0.421843886  0.37173976  0.26876656  0.633374771  0.350083459  0.194403499  0.385725204  -0.253341301  -0.715263473  -0.272624698  -1.14654498  -0.37027822  0.464979203  0.377744452  0.269631212  0.1765248  0.455132671  0.350541742  -0.423101271  -0.568961711  -0.492708407  0.271926755  -0.255673562  0.330451666  -0.321842736  0.678728606  -0.69056666  -0.301926305  -0.340033308  0.352826611  0.197095419  -0.479707988  -0.349434154  -0.32109308  -0.317336339  0.33869782  0.303193993  0.758538236  0.516662841  -0.389441393  0.180430256  -0.325500989  -0.366729667  0.177947296  0.306675313  -0.369741431  1.213265378  0.315685403  -0.487217938  0.284843739  0.243939871  0.43483392  -0.8192857  0.365508243  -0.436749698  -0.271538916  0.290023251  0.296976853  -0.359654015  0.252656622  0.427642287  -0.346969395  0.319873401  -0.400086642  0.441477804  0.38015478  0.209716304  -1.036650716  1.245496088  0.321006628  -0.517773742  -0.203363283  -0.299586602  -1.052157091  -0.316154578  0.25774777  0.238911907  0.278403248  1.889016409  0.329750025  1.774749685  -0.656435592  0.212347837  0.341203909  0.418199964  0.203939489  0.314122523  -0.468248603  -0.214174832  -2.310363865  -0.782419116  -0.277816526  0.261009297  0.303970137  -0.605582816  0.188945116  0.274654456  -0.687087815  -0.314433601  -0.300691649  0.561503256  -0.755601598  -0.281939964  0.330787913  0.201950133  0.293667302  -0.207126192  0.454619115  0.321120156  -0.74859508  -0.190535171  0.244181846  -0.308151138  0.258092397  -0.246880152  -0.326693579  0.219428268  0.310073966  0.523106971  0.247997265  -0.424757674  -0.302560205  0.519789557  0.25823651  -0.452440389  -0.232073636  -0.412599101  0.434073692  0.236083812  -0.291008882  0.369668756  -0.81570019  0.474839278  -0.222913152  0.440472376  0.214043601  -0.531081164  -0.218203251  0.441529715  -0.22104083  -0.197579045  -0.260787001  0.194798312  0.286098147  0.320352355  0.428046987  0.359840754  0.520960405  0.347081934  -0.260818048  -0.41278797  0.309095086  -0.385466607  -0.261029272  -0.932293232  -1.058683269  -0.547773988  -0.413330201  -0.235304537  -0.177381891  0.311245108  -1.134666243  -0.779849565  0.188065974  -0.338897682  0.300021377  -0.310405191  -0.71501393  0.325307861  0.577826293  0.499198753  0.371612878  0.271566432  0.275439374  0.430748621  -0.206495166  0.91301892  -0.738440944  -0.331089518  -0.895918401  -0.305641254  0.214700549  -0.368499935  -0.458534137  0.58723288  -0.36643337  0.243432907  -0.305724722  -0.404350724  0.426877207  0.179854514  0.408878852  -0.27491971  -0.467860438  -0.302816709  0.267919859  -1.250418138  0.914553717  0.468410957  0.371359233  -0.29113765  -0.360227571  -0.208882273  -0.359945876  0.487137608  -0.445498583  -0.374362548  0.364539762  0.282608816  -0.364299288  -0.289736121  0.204240463  -0.338566184  -0.268333799  0.248725971  0.361747995  -0.480628511  0.342240573  0.762779982  -0.541021268  -0.169846476  -0.273686253  -0.403073211  -0.437842592  0.242902321  0.230842299  0.595189373  -0.343092554  -0.335389483  -0.684310344  -0.260673855  0.261902089  -0.250429938  -0.504128654  -0.449494416  -0.341192412  0.324621404  0.287111551  -0.400280569  -0.227014503  0.273397731  0.238203156  -0.596434707  0.231982033  0.353471655  -0.358365826  0.254413515  -0.336222196  -0.277805819  0.573692517  -0.289400441  0.241058531  -0.446430734  0.422870993  -0.494362639  -0.328148145  -0.368804363  0.250781886  -0.456139022  0.210831934  -0.999813883  0.286190476  -0.303791441  0.554813517  0.619456213  0.493422915  0.873156146  -0.631151482  0.579822431  0.862876756  0.265031471  0.482833911  -0.35220515  -0.346510186  -0.458068592  -0.395908269  -0.293017774  -0.31356583  -0.370965584  1.214218769  0.272369164  -0.2975985  -0.229985628  0.406072067  -0.297084225  -0.277697986  0.273532781  -0.331628084  0.617693189  -0.578158822  0.356604976  0.35084524  -1.44664091  -0.244716269  -0.352849888  0.249455073  -0.562179368  -0.818129384  -0.626656117  0.345010652  0.320861567  -1.497537942  -0.321094087  -0.45899339  -2.950768357  0.210784902  -0.366762928  -0.259364529  0.241167121  -1.013228839  -0.754192218  0.343171066  -0.994882937  0.243487947  -0.253581474  -0.29283675  0.303190305  0.315931539  0.220010104  -0.252412836  0.291818022  0.244496866  0.450938561  -0.237839327  -0.220095081  -0.694272362  -0.629382344  -0.211030712  0.302899603  -0.214297482  0.258254144  0.850964435  -1.080420926  0.247445  0.43073383  0.373411719  -0.422409523  0.349238477  -0.299977782  -1.318580388  0.320548553  -0.313063974  0.26866143  -0.252012339  -0.289422906  0.271780251  0.25587824  -0.378779807  -0.426663187  -0.287774195  0.32429412  0.28269588  0.337484598  0.329483411  -0.198107935  0.223446623  -0.720490451  -0.246055616  -0.440156742  0.227193146  -0.279606481  -0.284987783  0.360541226  -0.398097697  0.283078966  1.012671447  0.5768186  -0.554231992  0.215243568  0.277202061  -0.481955089  -0.549245041  0.687638438  -0.312224966  0.267494788  0.307265602  0.281576516  0.22004372  0.302921542  0.213649545  -0.333745279  0.257740513  0.416046389  -0.565154464  -0.436969841  0.33607367  -0.703164082  0.373890378  -0.222648077  0.398046773  -0.632895523  -0.246409519  0.325504506  0.358831588  -0.818590637  -0.515835296  0.912252378  -0.442439624  -0.331211698  0.460735772  0.436366947  -0.677193438  -0.38869661  0.265083772  0.275500792  -0.455505316  0.359733609  -0.354282287  -0.446373912  0.287674528  0.29820595  -0.302624655  0.311120902  -0.585699969  0.309412495  -0.250751243  -0.362897681  0.354241866  0.285179991  -0.426021562  0.303370048  -0.329187599  -0.390850369  2.191472635  0.297475647  -0.536839485  0.860514072  -0.258386079  -0.405431818  -0.870582556  -0.344058028  0.222595519  0.625167677  0.258033925  -0.296184727  -0.369944117  -0.752371673  0.276452268  -0.3194926  -0.391247842  -0.28416411  -0.419201639  0.545846974  0.236157619  -0.197000231  0.264192043  0.412854347  -0.499897711  0.536477302  -0.318678292  -0.232887765  0.299704357  0.43983979  0.402264929  -0.298120199  -0.424201031  -0.177857354  0.192412766  -0.233582817  0.266380068  -0.455420449  0.232215677  -0.268281334  0.413714204  0.336744896  -0.814998942  0.46280297  -0.357888903  -0.244244444  0.421878178  -0.247911387  -0.34147586  -0.40014731  1.358900956  -0.20561017  -0.24886155  -0.197502336  -0.546347209  -0.374735306  0.256004365  -0.298863135  0.195467914  0.273848627  0.629233792  0.188763747  1.169920048  -0.299189424  -0.220165554  -0.659369326  -0.422298911  0.451562386  1.30302085  -0.357440709  0.257172545  0.282064495  0.309127791  0.310595056  -0.456601505  -0.425088038  -0.20834066  0.283829509  -0.332737436  0.333402197  -0.354621588  -0.238249023  -0.350611889  0.395504665  0.379997219  -0.285506236  -0.313358074  0.406118684  -0.332053645  0.305721326  -2.227630779  -1.451338251  0.206627873  -0.371039453  0.261794931  -0.195185323  0.300307138  0.311757035  -0.29347689  0.569163491  0.339355788  -0.596958066  -0.31707978  -0.767519091  -0.273068091  0.318908624  -0.573880498  -0.230535974  -0.434213303  1.701191987  1.092865582  0.265801506  -0.350474079  0.248672504  0.378052275  -0.268108583  1.529637832  0.581388421  0.526698421  0.409194379  0.162183114  0.326148635  0.423659354  -0.59119519  0.509431787  0.219191622  2.30923266  -0.250996887  0.23351962  0.445707847  0.289318827  -0.335631776  0.273251467  0.211938536  0.230254418  -0.680876944  0.277766774  0.320009283  1.668617686  -0.293509987  0.744448592  -0.162508642  -0.858713261  -0.362724441  0.436494174  0.299005372  -0.434344736  0.226963878  0.713924489  0.246496163  -0.542843757  -0.391638961  0.670486218  0.192707353  -0.965030892  -0.250819507  0.507647703  -0.316633658  -0.386452683  0.234387195  -0.251530642  -0.417056458  1.421095672  -1.174364527  -0.475040658  -0.397129527  0.550340235  -0.347220408  0.755151779  1.389118092  -0.227096426  -0.427474137  -0.579457263  -0.660516576  -0.744202617  -0.997015817  0.195034196  0.282599584  0.347099164  -0.383633403  0.224931207  -0.309027382  0.507564019  -0.287489142  -0.473156347  0.588382969  0.437992726  0.47452817  0.505636903  0.298918238  0.259958927  0.219398358  -0.520802796  -0.288648761  -0.223199678  0.594927413  -0.480559566  -0.296989358  0.526697303  -0.339241121  0.22552286  0.385939329  0.259175043  0.357792805  -1.325965289  -0.335962424  0.195925651  -0.59039825  -0.391992064  -0.640091145  0.944268024  0.196294042  -0.219853664  0.194748124  -0.469569585  -0.387054946  0.308785264  -0.44335504  0.205343242  -0.173129153  -0.656624118  0.335025055  -0.319809342  -0.205220729  0.233405051  -0.941950735  -0.361433834  0.466940555  0.199895845  -0.220933704  -1.221933121  -1.081928956  0.424524642  0.381142145  0.250550999  0.283840383  0.200466754  -0.339392989  -0.220293139  -0.198574827  -0.318888233  -0.27108211  -0.709765145  0.853815233  0.49713859  -1.607623068  -0.273110349  0.218618023  -0.302871942  -0.396503448  0.197445669  0.206970405  0.263275415  0.216162809  0.719571628  0.389506509  -0.943373225  0.269707692  0.148932561  -0.230079086  -0.449641647  -0.340315574  -0.192609595  -0.24461954  1.603741  -0.288069014  0.15337924  0.802141275  0.330481058  -0.212549692  0.303762823  0.506412431  0.242963387  -2.917255694  0.231695468  0.648771867  0.277629832  -0.183857288  -0.285507795  0.326928369  0.22084708  -0.420653866  0.31868539  -0.215246049  0.894491976  -0.735671316  -0.498196971  0.224715081  -0.293865734  -0.271312689  -0.304005478  0.175209251  -0.528656459  -0.319832049  -0.55335082  -0.311179352  -0.235011272  0.396813026  0.205976329  -0.91050579  0.260008574  0.455771951  -0.299272599  0.203292565  0.365761479  -0.326553535  0.426349923  0.4251662  -0.209347759  0.289082203  -0.457566203  0.18376337  -0.925303293  0.250785198  -0.461833999  -0.288578531  -0.305813196  0.305744826  -0.329267114  -0.299354541  -0.276653692  -0.188604971  0.307662028  -0.266750447  1.456326447  -0.446718556  -0.422291592  0.250513538  -0.797018502  -0.380923711  0.228749163  -0.215869808  -0.417601416  -0.214433812  0.610031347  -1.30143963  0.19454746  0.320912338  0.28431932  0.713115541  -0.259136767  -0.244863685  -0.778036576  2.330106021  0.295453904  1.102410403  1.704066153  0.364682649  0.465074613  0.288361681  -0.420961672  0.67012464  0.301941323  -0.461689532  0.917333468  -0.640414946  -0.166106485  0.178046474  -0.411580185  -0.667256488  0.244655303  0.22713238  -0.37677492  0.263037747  -0.196498124  -0.21325099  0.197133754  -0.356289699  -0.376086803  0.239049444  -0.205055267  0.176419809  0.497798151  0.498857785  -0.479406685  -2.181682978  -0.41977955  0.186296185  -0.343839707  -0.404242753  -0.236578583  0.21898783  -0.416275213  0.608528385  0.429410606  1.123590804  0.335633268  -0.300458088  -0.737512365  0.15044544  0.26354037  0.563570891  -0.283442261  -0.956108708  1.585649941  -0.374041564  -0.222679209  -0.270911744  0.241931669  0.243211097  -0.873031551  0.717937819  -0.271196517  -0.188547393  0.259242049  -1.708999048  -0.4040203  -0.239818951  -0.360452602  -0.244319695  1.618677082  0.464257346  0.312455421  0.312906967  -1.858690401  -0.536147242  -0.195820769  -1.382330667  -0.27929323  0.408478834  0.388786959  0.271012722  -1.075683576  0.723105414  -0.158595269  0.415634038  -0.452868493  -0.41294771  -0.451829482  0.310998561  -0.219590179  0.379843171  -0.288868613  -0.300285834  0.971827112  -0.324622553  0.262718252  -0.781645401  0.248066064  -0.643294893  -0.357909281  0.502430931  -0.347882147  -0.816876871  -0.957924413  -0.328380174  -0.205414304  -0.892727699  -0.197502837  -0.198456479  -0.796478552  -0.232044239  0.308103367  0.547151687  0.660986667  0.257138421  0.324893465  0.532155041  -0.572744218  0.269515092  0.314018209  0.19579484  0.175214961  0.318962712  0.252613616  -0.218245478  -0.213100677  0.263897116  0.349195007  0.164373925  -0.447120693  0.256738528  -0.586652415  0.264902315  0.162526397  -0.200815716  -1.057760842  -0.447663276  -0.48388928  0.419114207  -0.33969963  0.463852607  -0.238793035  -0.381715462  0.938703827  -0.487689836  -0.79027054  0.283504918  0.514288761  -0.25316429  -0.360136258  0.778723876  0.299975315  0.247120722  0.600932135  0.292645215  -0.48695951  -0.739182638  0.274421143  0.318014362  0.257304433  1.674246598  0.279163408  -0.261906676  0.25335266  -0.586947301  0.217752049  -0.247658515  -1.255597936  0.187275981  -0.235869304  -0.209905405  0.143106654  1.230194267  -0.754261488  -0.156530797  -0.186454872  -0.356396181  -0.189441052  -0.313413551  0.616801946  0.324963427  0.480103345  0.258077521  0.319673417  0.369053866  0.283628359  0.254939076  0.275324219  -0.207041254  0.272774745  -0.447176277  0.223008218  -0.658898839  -0.711271796  0.273258252  -0.404392404  0.725600368  0.265338763  -0.260386358  -0.436790634  0.277475253  -0.483654342  0.206998536  0.135430552  0.33871546  -0.477254895  -0.283123564  -0.44478658  0.783898221  0.466035861  -0.210273793  0.340268337  1.238225656  1.235145536  -0.723841062  -0.211861269  0.415849829  0.212029736  0.343226156  1.530239288  0.336072512  -0.389373768  -1.042773866  -0.646015989  0.415019553  -0.444812686  0.202872147  0.168726886  0.221058506  1.120640001  -0.403440306  0.168370832  -0.346576097  0.170384605  -0.41707775  0.354250546  0.255882108  -0.231921792  -0.50593903  0.380265278  0.185558772  0.299797765  -0.229027175  0.214892272  -0.308636678  1.721897014  -0.32234629  -0.216483006  -0.517724131  -0.379334556  0.362624252  0.248639739  0.639804969  -0.227128994  0.466060048  -0.387511368  1.332682136  -0.248882625  -0.624788216  -0.523832486  0.502422021  -0.523504582  0.785018134  0.4214015  -0.632128705  -0.602389901  0.222634439  -0.265658924  -0.806011086  0.295043223  0.538615228  0.152919694  -0.351815106  0.192137678  0.195752004  -0.262379892  -1.035025996  -0.740261045  -0.401908641  0.448394954  -0.822780579  -0.250148134  0.190632729  -0.520451057  0.262076927  0.799664988  -0.295403222  0.180519804  0.259391342  0.222398193  0.422324792  0.475529332  0.20874633  0.263680486  0.197877498  0.228811816  0.552853779  -0.502074727  0.613356942  -0.604491614  0.216079244  0.352409034  -0.622640681  0.418320134  -0.310934789  0.17273369  -0.449019374  0.185284459  0.187556803  -0.260006077  0.223030684  0.322612569  -0.185765766  0.165780051  0.324468213  -0.333270847  -0.456942647  -1.034308968  -0.352863682  -0.765219964  0.218763055  -0.450718787  0.237998419  0.199593602  0.244540324  0.24174031  0.292675533  -0.525025009  0.895004576  -0.814962895  -0.221078247  -0.919868456  0.250821211  1.671571881  -0.635178953  0.190205309  0.202082358  -0.386657776  -0.374886329  0.351021132  -0.245799603  0.269916085  0.316991698  -0.320157209  0.30138002  -0.905629492  0.181142922  0.165268592  1.095957546  -0.285077901  -0.388945767  0.247507919  -0.294744101  -0.30914348  0.191238982  -1.569854079  -0.532619421  -0.279439787  -0.547562095  0.993703986  0.314405066  -0.355838123  -0.32539793  -0.33811342  0.210921999  -0.692474764  0.199894896  -0.169574907  0.169884875  -0.368946205  0.265892074  -0.749084683  -0.195106474  -0.266880868  1.050029357  0.404336113  -2.023146655  0.331880861  -0.336120452  0.401066987  -0.229613204  0.285827328  -0.349553751  0.197608343  -0.287669036  -3.163746646  -0.286480298  -0.705751126  -1.453212424  -0.452904466  0.283776497  -0.212356537  1.113368017  1.214783674  -0.328767745  -0.68201739  0.196347346  0.333270155  -0.221514095  0.762866882  -0.767656812  0.197328639  -0.253565322  0.287165677  0.312720367  -0.331173979  -0.390900418  -0.371831179  -0.191916385  0.223024444  0.279965702  -0.449943326  0.314022244  0.282451187  0.486549704  0.513823523  -0.298791036  -0.460077585  -0.23370053  0.160767459  -0.263351188  0.271933824  0.331466937  0.400686892  -0.261021697  -0.412646408  0.253737565  0.1597546  0.256350385  -0.487232961  -0.256121001  -0.296111063  0.384066693  -0.134499714  -0.285442565  -0.500406271  0.384531578  -0.497133808  0.258691804  0.710256468  0.27556761  0.298012741  -0.179269252  -1.253071125  0.256405485  0.213155019  -0.548810245  -0.292918984  -0.677604186  -0.210847815  -0.453017502  -0.501110761  0.964577429  0.158763176  -0.577369822  0.889176229  0.153459662  0.297679777  0.209523153  0.238605876  0.275198226  0.810929755  -1.131359166  0.172605876  -0.356321085  -0.969862722  -0.461141217  0.411079553  0.807589818  -0.27709042  -0.445935246  -0.334285502  0.190027906  -0.422270835  -0.468064964  -2.022650531  0.4843161  -0.216484222  -0.275391768  0.277626388  -0.494155649  0.301961467  0.290489842  0.316059441  0.280777753  0.248599691  0.342435033  0.208744688  -0.390427133  0.387232289  -0.250065438  0.176600334  -0.237554499  -0.369195636  -0.349516135  -0.394253813  -0.236036737  0.317407364  0.16293767  -0.389413312  -0.324194719  -0.182143514  0.333610118  0.222283539  -3.256686561  -0.780435454  -0.241211751  0.345390653  -0.422039378  -0.51761568  0.176616362  -0.595689174  -0.276243121  0.182783912  -0.279526539  -0.163232555  -0.20876  0.723111096  0.545120803  -0.653330105  -0.405140928  1.062886685  -0.26937083  0.168125321  -0.193027973  -0.201792266  -0.2096447  0.81501929  -0.422164814  -0.296790188  0.316580571  0.320232302  -1.339370697  0.253240188  0.283804421  0.182791656  0.308822926  -0.252121738  0.203023958  -0.369676641  -0.22590482  0.922409217  0.414272282  -0.222176304  -0.132368092  -0.174723011  0.537985246  -0.271741718  -1.001599  0.266949553  0.293454888  -0.453907996  -0.214100671  0.292111542  -0.189121626  0.361618831  0.875564393  -0.258982819  -0.544010976  1.269612269  0.18591421  0.193981584  -0.507100382  -0.176533176  -0.284366079  -0.442510249  0.160360695  0.208029738  0.182571177  0.472216038  -1.219004854  0.262050458  0.595780604  0.857980746  0.358968179  -0.402449608  -0.263484791  -0.664083572  -0.479157146  0.505967898  -0.287404606  0.842385133  -0.481717599  0.248771115  -0.731141392  -0.446205087  -0.172453343  0.29837427  0.523226257  0.326869046  0.255575923  0.212583023  -0.405595369  -0.691284773  -0.215603464  0.409670808  -0.433748648  -0.22844968  -0.31066205  0.254840275  -0.20029684  -0.320079651  0.387534785  0.211466863  -0.237182286  0.205177848  -0.453583959  0.467578749  1.462220411  -1.79012197  -0.666180219  -0.386047484  0.387201468  1.691258208  -0.425792683  0.574176318  0.295036167  -0.229301405  -0.701875467  0.502057336  -0.171421479  0.162986039  0.562818323  0.22656198  -1.69079698  -0.293793966  0.177013455  -0.252143052  0.188597507  -0.230136599  1.113998021  0.177115049  0.420653348  0.325173464  -0.39464488  -0.483966401  0.203410474  0.406165396  0.405772926  0.303428857  -0.2135038  -0.169635384  -0.682444849  0.26487897  -0.247524432  0.402862805  -0.483549803  -0.343116843  0.177708487  0.273955119  -0.570651581  -0.239979692  -0.308332181  0.25362731  0.621735983  -1.600381039  0.174728658  -0.247311893  -0.203560188  0.224200877  -0.738489528  -0.516707689  -0.175467625  0.221199917  -0.168581373  0.313599799  -1.398402343  -1.262242608  0.210847168  -0.263954167  0.179342117  -0.237999173  -0.161000278  -0.237083569  1.293333824  -0.168630551  0.294071252  0.262995572  0.456090696  -0.185997077  0.261924684  0.586132165  0.294028479  0.252828358  -0.216455788  0.324345294  0.187403056  -0.291295192  0.339041818  0.377622979  -0.220414414  0.234975941  0.241519914  0.387786947  0.169239589  0.162001987  -0.280988728  0.464700376  -0.479938082  -0.193990346  -0.728673482  -0.384662919  -0.503146608  -0.792814044  -0.310373859  -0.70069555  -0.266969322  -0.196792321  -0.277960321  -2.505270065  -0.293810012  -0.262558786  -0.311006527  -0.253676101  -0.663619632  -0.268968403  0.625417355  0.194148583  0.29201725  0.254548272  -0.163696266  0.202127497  -0.646226035  0.784544797  0.29886811  0.184908959  -0.777813956  -0.401288768  -0.619848341  -0.295543908  -0.242170014  0.294415364  -0.247489162  0.706184612  -0.518913411  -0.17263812  0.223730374  0.161797916  -0.740681801  -0.247216558  0.271177037  0.620893011  -0.146983523  -3.136759624  -0.224514751  0.188290287  0.266872053  -0.319445367  -0.257954226  0.835623171  0.295774685  0.81875919  0.415680705  -0.180698888  0.456389781  -0.246759585  0.322140261  0.285505134  0.427666292  0.377808382  -0.231227821  -0.693669068  0.190609254  0.822978987  0.243610357  -0.21506133  -0.386242744  0.186482252  0.242235635  -0.549936858  0.154114603  -0.211119862  0.600503566  -0.390207753  -1.642990247  -0.19862728  0.226846366  0.25202007  -0.244564333  0.268100239  0.270757007  0.324368166  0.391279393  -0.159010594  -0.202662215  -0.60054626  2.000389691  -0.171494849  0.148458048  -0.22785863  -0.476241163  0.457235884  -0.2613529  0.192290455  -0.638697051  -0.266576305  0.367484346  0.204840651  0.780753495  -0.436857263  -0.688135372  0.183494024  -0.258600937  0.192255899  0.2090265  0.194193101  0.201579937  0.563255972  0.629335286  0.167747506  0.326308665  -0.563781615  -0.22170909  -0.962928968  0.186233789  0.234423454  -0.404929095  -0.26308947  0.615096619  0.255987459  0.209679702  -0.865832407  0.267518141  -2.735590941  -0.319674358  0.317717938  -0.171214439  0.325514785  -0.42356537  -0.338278  -0.256096837  -0.697256271  -0.197421749  1.18592629  0.160381401  -0.267134052  0.202653431  0.285428184  0.155323416  1.272124669  0.517614783  -0.296093148  -0.539203318  -0.232580953  1.634538526  -0.406402763  -0.234046685  -0.519469304  -0.509396244  0.808417124  -0.38602857  0.222421596  0.278968098  0.3730254  -0.761573597  -0.217373175  -0.220366234  0.493613429  0.496591386  0.386192574  -0.478786887  -0.257798515  -1.528865149  0.370992272  -0.214424869  -0.18306907  0.194821383  -0.383245501  -0.471571249  0.442207678  -0.249320993  0.330525338  0.226712411  0.22897629  -0.599448484  0.270866815  -0.334162667  -1.018349422  0.307241602  0.369646614  0.293383163  -0.221282145  -0.301055266  0.305536399  1.408859251  -0.75453564  -0.34214341  0.412632076  -0.188431133  0.34555212  0.313466934  -0.724914064  -1.086396755  0.45323855  -0.627775471  -0.426546956  -0.378780435  0.179978774  -0.79100059  0.160881105  -0.207059349  -2.956170941  -0.186606552  0.246852342  -0.280074638  0.311830708  1.020134854  0.66864342  0.335885442  1.412017731  -0.230613044  -0.523401509  0.281367073  0.66644918  -0.465026636  0.178003814  -0.660298518  -1.009057706  -0.585703075  -0.205658495  -0.614547242  0.971297763  0.34437527  -0.270366578  -0.14647281  -0.440365948  -0.616792961  0.168392711  -0.690967241  -0.159513334  -0.278364756  -0.184308439  -0.622291008  -0.296551121  -0.231683393  -0.283801582  -0.494568598  0.214933746  -0.266842132  0.389866135  0.163859685  -0.889066134  -0.334046401  -0.225141259  -0.326312367  0.365016935  0.210594322  1.2799897  0.155050888  -0.460294628  -0.229711867  0.367856297  -0.189620001  -0.286904265  -0.566855352  -0.591439382  -0.191210297  0.296154476  -0.2189974  0.309287777  0.25861705  0.178855058  -0.268958016  0.249852985  -0.372419362  0.29136616  -0.342148381  0.269619393  -0.512074761  -0.132023657  -0.274438166  -0.404845348  0.227511742  0.857238704  0.274737807  0.311235891  0.368119313  -0.348974733  0.20070761  -0.211055577  -0.229930773  -0.184630091  -0.226359213  -0.205338427  -0.133339896  -0.195415541  0.452638006  0.17460794  0.468966631  -0.294479193  -0.406590987  -1.428743179  0.184810253  0.352782036  0.175115219  -0.216877374  -0.212307844  0.313160355  0.292854386  -0.311200937  -0.738992491  0.380708398  -0.205105254  0.317828196  -0.22376105  -0.151050181  -0.275971175  0.925130335  -0.339010896  0.70461801  1.12561973  0.493307876  -1.068105842  0.307881489  -0.120956569  1.483374923  -0.226929212  -0.751394649  0.219091912  -0.31167436  -0.482159371  -0.200553594  -0.341905397  -0.298036905  -0.218158512  0.546764836  -0.401214365  0.255119877  0.225618012  0.168151193  -0.366721479  -0.313412001  0.159903274  -0.30570182  0.438997517  -1.142305335  -0.175812205  -0.707605402  -0.231945417  -0.239023247  0.46142759  -0.195073566  -0.25783258  0.204862547  -1.150671651  0.60868961  0.596727229  0.169886888  -0.260259204  0.19687715  0.152448096  0.351618679  0.385289888  -0.23536147  -0.36716144  -0.319433656  0.659489911  -0.243592481  0.205458892  -0.31712697  0.14697434  0.447943371  -0.214755013  -0.414908488  -0.196279246  -0.233281239  -0.251624213  1.050696154  -0.206520751  0.197454726  0.210199648  -0.40820819  0.275785285  0.533701736  0.200502691  0.320261224  -0.382848516  0.984909002  -0.20693143  -0.284314816  -0.209071731  -0.259854032  -0.131169554  -0.192559348  0.224932367  0.211187239  0.202172446  0.255741124  -0.385998776  -0.256281103  -1.000989399  -0.691606818  0.231219026  0.613213285  -0.348830581  -0.653342743  0.21373623  0.500287462  -0.395473821  -0.289171837  -1.126494435  -0.455785631  1.056119056  -0.170401854  0.123074197  -1.736291315  -0.445493393  0.378289484  -0.353144248  -1.192429289  0.182291186  -0.226825856  0.465717551  0.31685289  1.276940165  0.204757119  -0.358393482  -0.611331768  -2.61977045  0.142697953  0.244620995  0.278107897  -0.184999059  -0.200852471  0.190535304  0.305620762  0.245014862  -0.174593793  -0.488961644  -0.290103147  0.182685392  1.226695326  -0.766116049  0.259567738  0.266538191  0.204905101  -0.22287958  -0.365141494  -0.330007423  0.290681713  -0.24857009  -0.215901332  0.235378326  0.130868381  0.361481455  -0.243935541  -0.524478606  -0.267249492  -0.202702724  -0.581374649  0.362841457  0.335539007  -1.102822229  -0.247436925  0.193400809  0.6792683  0.156169849  0.2396094  0.114934995  -0.390148983  -0.305965212  -0.307568424  0.192186316  0.628655319  0.44547975  -0.418579191  0.847489867  -0.397399064  -0.156742855  0.248009288  0.216300824  -0.140893542  -0.642733578  -0.231361528  -0.422933117  0.238831722  -0.173152224  0.688145789  1.099401473  -0.409039523  -0.147476788  -0.228858198  -0.333368936  -0.421706674  -0.156475238  0.203469366  0.327257889  0.22627101  0.127475209  -0.495869907  0.982816141  -0.288616392  -0.122260037  0.408478762  -0.662844206  0.267320113  0.324379106  -0.282402869  -0.976726702  -0.766740846  0.238634924  -1.392504808  0.138118493  0.434666895  -0.282082809  0.224511366  0.463296162  0.355800283  0.331011234  -0.32185352  -0.655203711  0.208587389  -0.442097709  -0.259728714  0.360957419  -1.215649759  -0.256629331  -0.257105037  -0.172557916  0.583591186  0.271095475  0.415554185  -0.258044241  -0.223702871  0.301193304  -0.145405154  0.133624887  0.700065569  0.178869677  0.295411863  -0.240605084  -0.151943927  -0.298070916  0.241060374  0.333182595  0.342108702  -0.771834355  -0.352165644  -0.243630214  0.359354232  0.142901523  -0.396516665  -0.165967195  -0.294708387  0.434065627  -0.475768537  -0.164133687  0.216583331  -0.173429658  -0.210451909  -0.270201416  -0.326847919  -0.192596121  -0.573647688  -0.168364543  -0.243795812  0.220704992  0.216423924  0.399781187  -0.381012381  -0.246480164  -0.57560314  -0.824982055  -0.560654363  0.11937404  -0.201498395  0.393775264  -1.068390203  1.109702841  -0.198088072  0.256280691  0.140555794  0.339369674  0.931665913  -0.31183553  -0.833373895  0.652424186  -0.204508615  0.259774084  -0.92097995  -1.293578656  1.036247984  -1.488117973  -0.462157703  0.194708191  -0.205979953  -0.227104189  1.262780298  -0.350512663  0.208426828  1.350318907  0.138281167  0.977187504  0.780746252  0.541866693  0.160969219  0.176434339  0.144652237  0.81313687  0.442796255  0.302240607  -0.335738524  0.160283718  -0.13628161  0.182299594  -0.467869032  -0.972175462  0.40820919  0.526049664  0.137051729  -0.386797894  -0.189687064  0.171452819  -0.234487498  0.146813879  -0.393947364  -0.158850213  -0.497450235  0.216386908  0.236641509  -0.19512979  -0.151974196  -0.207771113  -0.547124605  0.402217584  0.250813831  0.292695944  -0.181624646  0.33176894  -0.425144924  -0.218906262  0.238183768  -0.105635186  0.304108248  -0.245158633  0.146525417  -0.1444922  0.272956551  -0.181143307  -0.174604225  -0.282823625  -0.7298534  -0.571291554  -0.167277041  0.200359017  1.076007852  -0.153670233  0.160087926  -0.390718744  0.194970541  -0.662700521  0.242760003  -1.660279057  -0.168792777  -0.447106093  0.298180512  -0.447771689  -0.865437232  -0.192902244  0.258201859  -0.288120322  -0.202824256  0.148246199  -0.233134476  0.205850706  0.360209066  0.458292428  0.418234572  0.282794329  0.509795921  0.176121568  -0.206007987  -0.255303982  -0.531964135  0.166732575  0.26836625  0.598956732  -0.204293548  -0.153919966  0.220095006  -0.28960762  0.262497179  -0.149384071  0.15348719  0.141238928  0.148834088  0.298137051  -0.142572654  0.231472528  -0.170614521  -0.438008939  -0.254977439  -0.212123471  -0.13692556  0.308456874  0.36283685  0.485948611  -0.376742522  -0.192280117  0.37862503  -0.189080764  -0.227541993  -0.45024869  -0.262620926  -0.67269892  -0.426786605  0.193370001  -0.192584433  0.202086827  0.80110486  0.263593896  -0.232907772  -0.33983921  0.320474426  -0.171453946  -0.242869829  0.391491573  0.137719137  -0.303828987  -0.504504208  0.133918145  -0.316035242  0.297659441  -0.281169479  0.315470592  -0.364323352  -0.18525305  0.587602366  -0.882093822  -0.279606149  -0.157092006  0.217567646  0.247332348  -0.23637321  0.224878562  0.326770143  0.445826763  0.215660556  -0.608766196  0.224031147  -0.238134322  0.147276084  -0.264410791  0.373291773  -0.230932982  -0.162691292  -0.91056757  0.306437055  -0.397453399  -0.166342643  0.333545126  -0.152519322  -0.183790973  0.471384157  -0.233613461  0.429641016  0.220327863  0.346217052  -0.587378086  0.141832122  -0.25377832  0.336266199  0.292021292  -0.167738036  -0.176097637  0.217294619  0.261869392  0.301339389  0.916606918  -0.54228926  -0.318563283  -1.537511801  -0.339062242  -0.364560165  -0.584722784  -0.352856477  0.145975102  -0.122452439  -0.250730491  0.26696408  0.928502771  -0.256817328  0.281589619  -0.259841982  0.237759637  -0.335052229  0.156225275  -0.330620503  0.352465824  -0.722301498  -0.193172587  0.297566639  0.34195113  -0.657858569  0.191074026  0.248519319  -0.739120585  -0.169641445  -0.21039247  0.982479781  0.796634782  -0.192937082  -0.158060182  -0.133093933  -1.380779736  -0.332502344  -0.991401948  1.581167964  -0.481715801  1.22853168  -0.427201916  0.197784744  -0.259170052  -0.263224454  -0.193480733  -1.614420837  -0.258046979  -0.21380949  0.245453  0.466087216  -0.281977583  -0.152656124  -1.190952791  0.216119492  0.190240514  0.23506306  0.785066517  -0.139379086  -0.421482854  0.633857056  -1.008128119  0.167471614  -0.151291331  -1.499517675  -0.128388338  -0.150694853  -0.382427945  -0.390768949  -0.284310737  0.212213039  -0.329308862  0.328566339  0.191130583  0.331392645  -0.175860967  -0.247990748  -1.602885259  0.667567754  -1.247761812  0.7733818  0.159467472  0.476030074  -0.331262721  0.145566324  0.233032733  1.346396602  -0.136939643  0.207188922  -0.649520663  0.309728051  0.208679429  0.339226013  -0.132380517  -0.478956502  0.257685179  0.134484607  -0.182312422  0.426802456  0.226582612  0.135435876  -0.111177823  0.143546943  -2.711413154  0.150741105  -0.130818424  0.207819112  0.697399398  1.072339274  0.695325261  -0.843864418  -0.508778307  -0.806992991  -0.281859988  0.267671967  0.361532261  -0.129677904  -1.112352214  -0.412532446  0.256587574  0.354755705  -0.160798809  -0.349024113  0.182918161  -0.163100763  -1.099124402  -0.460980176  -0.182229657  0.236080953  0.130023085  -0.527558094  -0.628583938  0.17611356  -0.191317196  0.145200077  0.197028512  -0.545012133  -0.346981606  -0.570346665  0.111520267  -0.325097975  0.248618005  -0.339877434  0.732313672  -0.79597634  -0.175657606  0.12939171  0.336684631  0.666633769  0.164037105  0.537554014  -0.212199849  0.341600503  0.159608977  -0.179771565  -0.843959055  -1.259770061  0.269678409  -0.345161892  -0.17989017  -0.258166298  1.535778793  0.11508414  0.171524175  0.18679544  0.25293335  0.146865495  0.219666754  0.228277049  -0.354988564  -0.153988477  0.107534422  -0.161138933  -0.255230823  0.972957085  -0.308174533  -0.129539775  0.243398788  0.537149964  -0.187391012  -1.530207705  0.212966462  0.298616781  -0.120487221  0.386185189  0.188701279  -0.376976131  0.189334916  0.847971628  0.16002563  -0.477698402  0.282516364  -0.227042866  0.180289439  0.349030408  0.303479873  0.181068365  -0.187883492  -0.156072467  0.135561518  -0.477665729  0.149548318  0.179065214  0.288024583  1.49547174  0.295917856  -0.161705277  -1.085182554  -0.888593533  0.562865021  0.920194852  1.561559038  0.481555855  -0.239182904  0.325527164  0.149981264  0.670090977  0.271865777  0.175639838  -1.725486657  -0.195395937  0.295962457  0.259094137  -2.574463547  0.3457506  0.588849759  -1.151601942  0.127338478  0.134365022  -0.154466148  -0.300334509  0.226490243  -0.227441466  0.538960818  -0.175717931  -0.183675884  -0.455240895  0.274959288  0.13053486  -0.144450118  0.324922688  -0.472919965  -1.341254549  -0.36556016  -0.332918906  -0.777243942  -0.357273916  -0.196695641  0.235270261  0.1945017  -0.196532334  -0.552591136  -1.728116889  -0.570684182  0.794078075  -0.472053299  -0.359865655  0.297407688  -0.484216717  -0.287601695  0.549164258  -1.388662362  0.516159004  0.466616531  0.240353655  -0.145754804  0.17463114  0.135613588  -0.502882032  -0.723967215  -0.146161923  -0.16794395  0.192706178  -0.833061037  -0.657690243  0.270783848  -0.666091658  -0.242747429  -0.308841949  -0.2287463  0.153420067  0.421862766  0.187309806  1.554393715  -0.285329001  -0.159881309  0.140573703  1.117623071  0.315298355  0.245923591  0.313155537  0.198097565  0.783180529  0.191605336  -0.734008564  0.667650266  0.305050044  -1.310009293  0.328668934  -1.023839102  0.190196884  -0.276244465  -0.166975865  -0.162026337  0.170911837  0.355164057  0.440245168  -0.232456541  0.474281389  -0.472422281  -0.19034509  -0.901894791  -0.213489688  0.361474465  1.574300471  0.19452551  -0.195509655  0.281785043  -0.346956529  0.317923724  -0.484691325  -0.203643648  0.590972828  0.393465554  -0.471076257  -0.878540694  -0.317974871  0.141114412  -0.247226205  -0.180673473  -0.243167293  0.148129685  -0.327763449  -0.25068262  0.312826911  0.254302884  -1.872788269  -0.215165979  -0.336888454  -0.251390121  -0.951723371  -0.650887333  -0.295269172  -0.308801021  -0.281162727  0.128980321  0.122308089  0.547964468  -0.323074876  -0.262581855  0.464406226  0.201307713  0.3508885  -1.216365447  -0.438128258  -0.332729873  -0.451929393  0.164269628  -0.959498815  -0.428658544  -0.281362506  -0.155815019  -0.138932004  0.227773756  -0.22007578  -0.403425517  -0.218059873  0.150022739  -0.140390072  -0.205273428  0.149374062  -0.192720057  0.281180458  1.410508404  -0.168167238  -0.313965494  0.170555895  0.266997989  0.202458907  1.743613424  -0.365763843  -0.536037896  -0.227109709  0.339359303  0.119964966  0.251887579  -0.720959335  -0.319158366  -0.216556497  0.273154164  0.290950154  0.181709497  -0.710007619  -0.266727807  0.136557953  0.580531221  -0.170448736  -0.12239372  -0.481732646  -0.726959298  -0.403374614  0.425980382  -0.464118843  0.191767701  -0.195439649  -0.884535407  0.409902157  0.254675644  -0.129417258  -0.408825812  0.20677693  -1.117243527  -0.243488847  0.125181097  -0.14742041  0.147641494  -0.282277017  -0.47138798  -0.282516184  -0.510477661  -0.275289221  0.24115574  0.399474006  -0.205905439  -0.230057832  -0.303554871  0.167484488  0.140807371  0.445632591  0.220328571  -0.925530699  0.392251581  -0.167361038  -0.946056513  0.423409384  -0.545562059  -0.268128099  0.131767928  0.258714068  0.109412582  0.15075845  -0.945397419  0.25344639  -0.18973199  0.163545793  -0.481714176  0.136302061  -0.722576086  -1.245489851  -0.337880983  0.139587645  -0.42636345  0.297091472  -0.507977117  0.347732687  0.184948649  0.420150537  0.209630215  -0.137313261  -0.305229962  0.148987474  -0.1804254  1.446443652  -0.368449264  -0.216860324  -0.163969163  -0.121776759  -0.222061819  0.309011036  -0.298212085  0.123445932  -0.761748325  -0.162671073  -0.118369352  -0.329915782  0.358988627  0.271210277  -0.230044144  -0.597835282  -0.15757209  -0.467213116  -1.473411027  0.186861324  -0.308782368  1.090120603  0.191671829  0.121884354  -0.254106527  -0.200031954  -0.423217004  0.515503281  -0.10266242  0.187826876  0.211450311  -0.26664251  -0.222009333  1.023592476  -0.457261984  -0.208703555  -0.502543489  -0.28455145  -0.440799791  -0.164583074  -0.189960846  0.148071911  0.360458637  0.471041489  -0.349849976  -0.30839325  0.22781278  0.163596253  -0.312611368  -1.064960967  0.1106281  -0.196472091  -0.773553791  -0.864004558  -0.483064145  -0.574565186  0.384124187  0.17924959  -0.348704726  -0.649693024  -0.952152796  0.157458369  0.276989567  -0.197241855  0.179931002  0.226528765  0.748106634  -0.74856503  -0.735407327  -0.267755265  -0.127839648  0.145312685  -0.198175773  0.160129742  0.142812112  0.276696472  0.199974991  -0.48669791  1.046064744  -0.279995498  -0.462194053  -0.669558907  -0.224228537  -0.469802748  -0.771100138  0.127234239  -0.22529241  -0.172421436  -0.359579514  -0.345206336  -0.160238106  -0.232271104  -0.516763027  -0.307137646  0.283389209  -0.330344311  1.430334572  -0.184302349  0.393382054  -0.690031106  -1.081751051  0.621718731  -0.81648185  0.23943997  0.834043983  -0.171868674  -0.133363989  0.147491115  -0.26194967  0.117775293  -0.384981926  -0.168721025  -0.127874652  -0.342247684  0.156552345  0.168908496  -0.156526174  0.209270776  -0.176024948  -0.259181211  0.214085033  -0.136445339  0.308129444  -0.155218532  0.324285113  0.182751388  -0.283498666  0.492773236  0.113015262  0.185336647  -0.529779532  -1.309277048  -0.306482844  0.552830264  -0.738864717  0.141772181  0.190271566  -0.238195333  -0.924149636  0.505997015  -0.1967454  0.164350701  1.102790167  0.238656395  -0.183218828  -0.126571484  0.25649955  0.106374781  0.141084203  -0.190882879  -0.413574758  0.345659765  0.145948047  -0.914772083  0.248581575  -0.307908866  1.408750704  -0.312624939  -1.309638896  -0.472244274  0.343989884  -0.410411497  0.291953715  -0.344109882  0.292803312  -1.119698986  -0.538335596  0.185536428  -0.210037717  -0.663793693  -0.466764232  -0.386800571  -0.214903979  -0.791141101  -0.172846395  -0.159176364  -1.285035444  0.198661609  -0.143507188  0.18115736  0.160679903  -0.932135867  -0.262096602  -0.515653474  0.155263898  -0.217074345  0.150168041  0.212024187  -0.23062219  0.701428937  0.345107767  -0.35109932  0.164941109  -0.129977899  0.502937413  0.171499915  -1.047833364  -0.092276202  0.200765249  -0.166361099  0.12357405  0.155734208  -0.131967455  0.181115642  -0.190962831  -0.670210701  0.179107697  -0.132507641  -0.403883931  0.136707974  -0.153836317  -0.715364236  -0.237734103  -0.274372775  -0.213160423  -0.606607326  0.460290362  0.261329576  0.272562506  0.747198257  -0.236889826  -0.212337885  -0.240228648  0.165004196  0.1873049  -0.651745243  -0.210313747  -0.248837798  0.149379533  -0.304772877  0.203822604  0.216948128  0.230424474  0.139009386  -0.292358282  0.169761361  0.522173379  0.184214008  0.802444461  0.204687993  0.134891377  -0.583691205  1.477408295  -0.157472521  -0.145749211  -0.247045096  -1.269294124  -0.196980171  -0.304696637  -0.371780295  -0.244026824  -0.300107905  0.126974716  0.826795129  0.228431149  -0.292438713  -0.704758767  0.163746561  -0.306519511  0.646886865  -0.143349385  -0.734388492  -0.258912777  -0.286545924  0.129116404  -0.129736658  -0.17862313  -0.152661808  -0.691154766  -0.993261268  0.954908905  0.802863518  -0.290385086  0.183982791  -0.166638324  0.184292167  0.144337913  -0.220995652  0.118823159  0.188479535  0.122845978  0.137864596  0.125412605  0.418356376  0.133923233  0.16356373  -0.421233775  -0.283689576  0.365837979  -0.3084097  0.604665609  -0.31262869  -0.149910829  -0.341072457  0.190650163  -0.182504345  0.515944119  -1.449165356  0.143901803  0.201122903  0.561668776  -1.146311269  0.435754564  0.322614586  -0.365710877  -0.301429906  0.177141166  0.174818281  -0.145568975  -0.445174911  -0.365499806  -0.154724855  -2.378026583  0.258256689  -0.166075067  0.642329907  -0.247649386  1.110631486  -0.194731097  0.14941407  0.712641269  -1.000996743  -1.083907615  1.000608938  -0.350524561  0.154886823  -0.491545849  0.121290697  0.201516366  -0.525693776 |
